# Zwitterionic DNA: enzymatic synthesis of hypermodified DNA bearing four different cationic substituents at all four nucleobases

**DOI:** 10.1093/nar/gkaf155

**Published:** 2025-03-08

**Authors:** Natalia Kuprikova, Marek Ondruš, Lucie Bednárová, Tomáš Kraus, Lenka Poštová Slavětínská, Veronika Sýkorová, Michal Hocek

**Affiliations:** Institute of Organic Chemistry and Biochemistry, Czech Academy of Sciences, Flemingovo nam. 2, CZ-16000, Prague 6, Czech Republic; Department of Organic Chemistry, Faculty of Science, Charles University, Hlavova 8, CZ-12843, Prague 2, Czech Republic; Institute of Organic Chemistry and Biochemistry, Czech Academy of Sciences, Flemingovo nam. 2, CZ-16000, Prague 6, Czech Republic; Institute of Organic Chemistry and Biochemistry, Czech Academy of Sciences, Flemingovo nam. 2, CZ-16000, Prague 6, Czech Republic; Institute of Organic Chemistry and Biochemistry, Czech Academy of Sciences, Flemingovo nam. 2, CZ-16000, Prague 6, Czech Republic; Institute of Organic Chemistry and Biochemistry, Czech Academy of Sciences, Flemingovo nam. 2, CZ-16000, Prague 6, Czech Republic; Institute of Organic Chemistry and Biochemistry, Czech Academy of Sciences, Flemingovo nam. 2, CZ-16000, Prague 6, Czech Republic; Institute of Organic Chemistry and Biochemistry, Czech Academy of Sciences, Flemingovo nam. 2, CZ-16000, Prague 6, Czech Republic; Department of Organic Chemistry, Faculty of Science, Charles University, Hlavova 8, CZ-12843, Prague 2, Czech Republic

## Abstract

We designed and synthesized a set of four 2′-deoxyribonucleoside 5′-*O*-triphosphates (dNTPs) bearing cationic substituents (protonated amino, methylamino, dimethylamino and trimethylammonium groups) attached to position 5 of pyrimidines or position 7 of 7-deazapurines through hex-1-ynyl or propargyl linker. These cationic dNTPs were studied as substrates in enzymatic synthesis of modified and hypermodified DNA using KOD XL DNA polymerase. In primer extension (PEX), we successfully obtained DNA containing one, two, three, or (all) four modified nucleotides, each bearing a different cationic modification. The cationic dNTPs were somewhat worse substrates compared to previously studied dNTPs bearing hydrophobic or anionic modifications, but the polymerase was still able to synthesize sequences up to 73 modified nucleotides. We also successfully combined one cationic modification with one anionic and two hydrophobic modifications in PEX. In polymerase chain reaction (PCR), we observed exponential amplification only in the case of one cationic modification, while the combination of more cationic nucleotides gave either very low amplification or no PCR product. The hypermodified oligonucleotides prepared by PEX were successfully re-PCRed and sequenced by Sanger sequencing. Biophysical studies of hybridization, denaturation, and circular dichroism spectroscopy showed that the presence of cationic modifications increases the stability of duplexes.

## Graphical Abstract

**Graphical Abstract:**
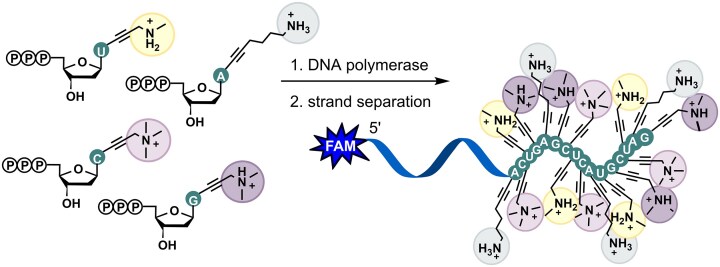

## Introduction

DNA containing modified nucleobases [1] are used in selections of DNAzymes [2–4] or aptamers[5–8], or in constructions of other functional nucleic acids and nanomaterials [9–11]. Their enzymatic synthesis from base-modified 2′-deoxyribonucleoside 5′-*O*-triphosphates (dNTPs) [12, 13] is of crucial importance, in particular for the *in vitro* selection approaches (i.e. SELEX). Most of the previous works have used one [14–16] or two modified dNTPs [17] in combination with the other three or two non-modified nucleotides and very few of them reported on hypermodified DNA [18–20] where each and every nucleotide is bearing a different modification. We have systematically studied approaches to hypermodified nucleic acids and developed methodologies for enzymatic synthesis of hypermodified DNA bearing four different hydrophobic arylalkynyl or alkylalkynyl substituents, obtained either through primer extension (PEX), asymmetric polymerase chain reaction (aPCR) [21] or reverse-transcription [22] using DNA polymerases. Later on, we used the PEX and aPCR to synthesize superanionic DNA bearing four different anionic groups, as well as combinations of two anionic and two hydrophobic substituents [23]. Very recently, we also reported synthesis of hypermodified DNA displaying four sugars [24] and an analogous synthesis of hypermodified RNA using PEX with engineered TGK DNA polymerase [25]. Interestingly, the hypermodified DNA containing hydrophobic alkynyl groups showed increased thermal stability, whereas the duplexes of superanionic DNA (containing double number of anions and thus facing stronger coulombic repulsion of the strands) showed significant decrease of melting temperatures but still sufficient for hybridization. To investigate the other extreme case of charge distribution, we report here the synthesis and study of hypermodified zwitterionic DNA containing a combination of four different cationic groups.

There have been many literature examples of base-modified dNTPs bearing cationic substituents (mostly amino or guanidine groups) and their use in polymerase synthesis of modified DNA. 5-Substituted aminopropargyl- [19, 26], aminoallyl- [26, 27], and 3-aminopropyluracil [26] or -cytosine dNTPs or the corresponding 7-substituted 7-deazaadenine dNTPs [19, 28, 29] were reported as rather poor but still useful substrates for DNA polymerases in PEX (but less so in PCR) and were used for selection of DNAzymes or for further modification of the nucleotides or DNA [30, 31]. Interestingly, in 5-aminopentynyl derivatives of dNTPs, the 7-substituted 7-deazaadenine and -7-deazaguanine nucleotides were significantly better substrates compared to the corresponding 5-substituted pyrimidine dNTPs [32]. Additionally, other works showed [33–35] that a longer linker between the protonated amino group and the nucleobase is beneficial for good substrate activity of the dNTPs. Several works also report on guanidine-linked dNTPs [4, 36] (as analogues of arginine side chain) and on a combination of amino-, guanidine-, and/or imidazole-linked nucleotides in DNA [28, 29, 4, 36], and one of the above mentioned seminal works reported aminopropargyl modification in combination with other non-cationic groups [19]. So far, there was no report on enzymatic synthesis and properties of hypermodified DNA containing all four different cationic modifications. Hence, we report here on the design and synthesis of four nucleotides, each modified with a different cationic group, and their application in polymerase synthesis of zwitterionic hypermodified DNA.

## Materials and methods

### Synthesis of modified dN^R^TPs (N = A, U, G) bearing primary, secondary, and tertiary amino groups

**Scheme 1.**
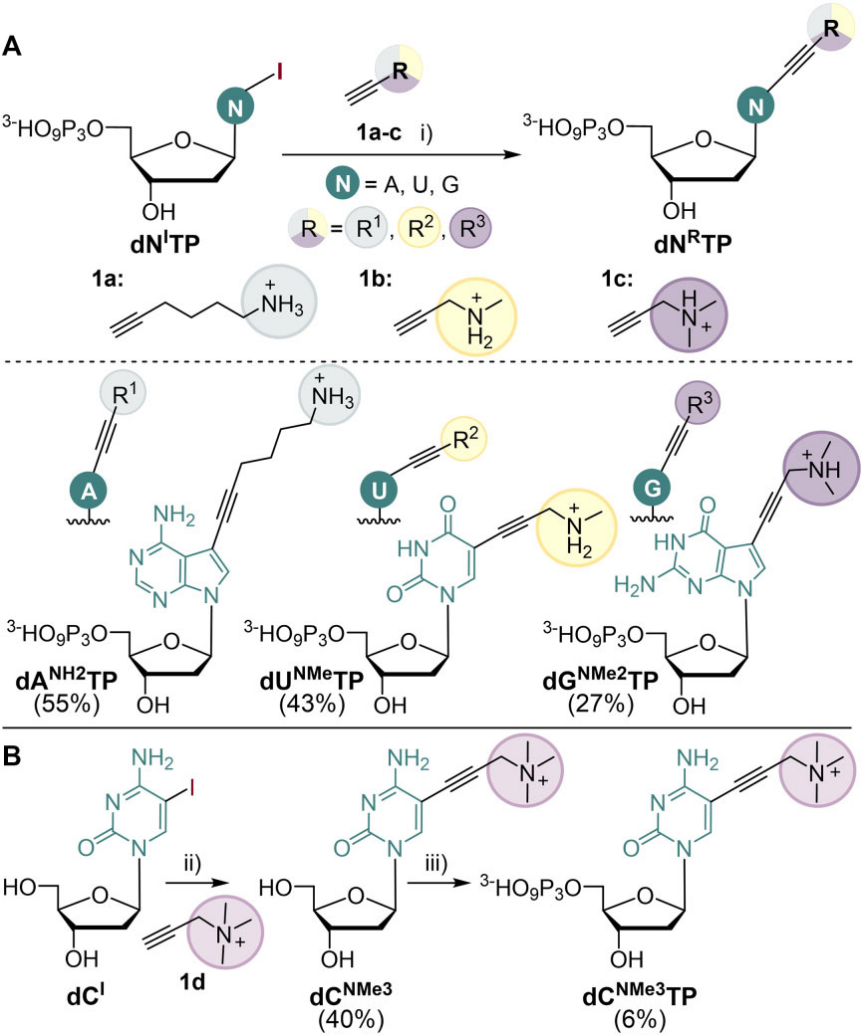
(**A**) Design and synthesis of **dA^NH2^TP**, **dU^NMe^TP**, and **dG^NMe2^TP**. Reagents and conditions: (i) R-C≡CH (7.5 equiv.), Pd(PPh_3_)_4_ (0.6 equiv.), CuI (1.2 equiv.), Et_3_N (4.5 equiv.), DMF, rt, 5 h, and under Ar, 27–55%. (**B**) Design and synthesis of **dC^NMe3^TP**. Reagents and conditions: (ii) R-C≡CH (7 equiv.), Pd(OAc)_2_ (0.4 equiv.), CuI (0.8 equiv.), TPPTS (0.6 equiv.), Et_3_N (10 equiv.), DMF, 60°C, 2.5 h, and under Ar, 40%; (iii) 1. POCl_3_ (1.2 equiv.), PO(OMe)_3_, 0°C, 45 min, under Ar; 2. (NHBu_3_)_2_H_2_P_2_O_7_ (5 equiv.), Bu_3_N (4 equiv.), MeCN, 0°C, 1 h, under Ar; 3. 2M TEAB.

Anhydrous DMF followed by Et_3_N (4.5 equiv.) were added through a septum to an argon-purged flask charged with a halogenated nucleoside triphosphate **dN^I^TP** (N = A, U, G) [37, 38] (1 equiv.), a corresponding alkyne **1a–c** (Scheme 1; Supplementary Scheme S1 in Supporting Information (SI)) (7.5 equiv.), CuI (1.2 equiv.), and Pd(PPh_3_)_4_ (0.6 equiv.). The mixture was stirred at room temperature for 5 h under argon atmosphere. The solvent was evaporated under vacuum. The product was purified by reverse-phase HPLC with a linear gradient of MeOH (0–100%) in 0.1 M triethylammonium bicarbonate (TEAB) buffer (pH 7.6) (for details see sections 7.1–7.3 in SI) followed by lyophilization to obtain a solid product (more details in section 1.1 in SI; information about the source of used substances can be found in General remarks in section 1 in SI).

### Synthesis of modified dC^NMe3^TPs bearing quaternary ammonium group

Anhydrous DMF followed by Et_3_N (0.4 mL, 2.83 mmol) was added through a septum to an argon-purged flask charged with 5-iodo-2′-deoxycytidine **dC^I^** (100 mg, 0.28 mmol), propargyltrimethylammonium iodide [39] (446.2 mg, 1.98 mmol) (Scheme 1; Supplementary Scheme S2 in SI), CuI (43.2 mg, 0.23 mmol), TPPTS (96.61 mg, 0.17 mmol), and Pd(OAc)_2_ (25.4 mg, 0.11 mmol). The mixture was stirred for 2.5 h at 60°C under argon atmosphere. The solvent was evaporated under vacuum. The product was purified by reverse-phase HPLC with a linear gradient of MeOH (0–100%; for details see section 7.4 in SI). Obtained modified nucleotide **dC^NMe3^** was further subjected to phosphorylation reaction following the standard phosphorylation procedure [40]. The purification of **dC^NMe3^TP** was done by reverse-phase HPLC with a linear gradient 0 to 100% of buffer B in buffer A (buffers’ composition is specified in General remarks in section 1 in SI) followed by an additional purification step using a Sepharose FF column with a linear gradient of 800 mM TEAB solution in H_2_O (0–100%) (more details in section 1.2 and section 7.5 in SI; information about the source of used substances can be found in General remarks in section 1 in SI).

### Multiple incorporation of four modified dN^R^TPs by PEX

**Figure 1.**
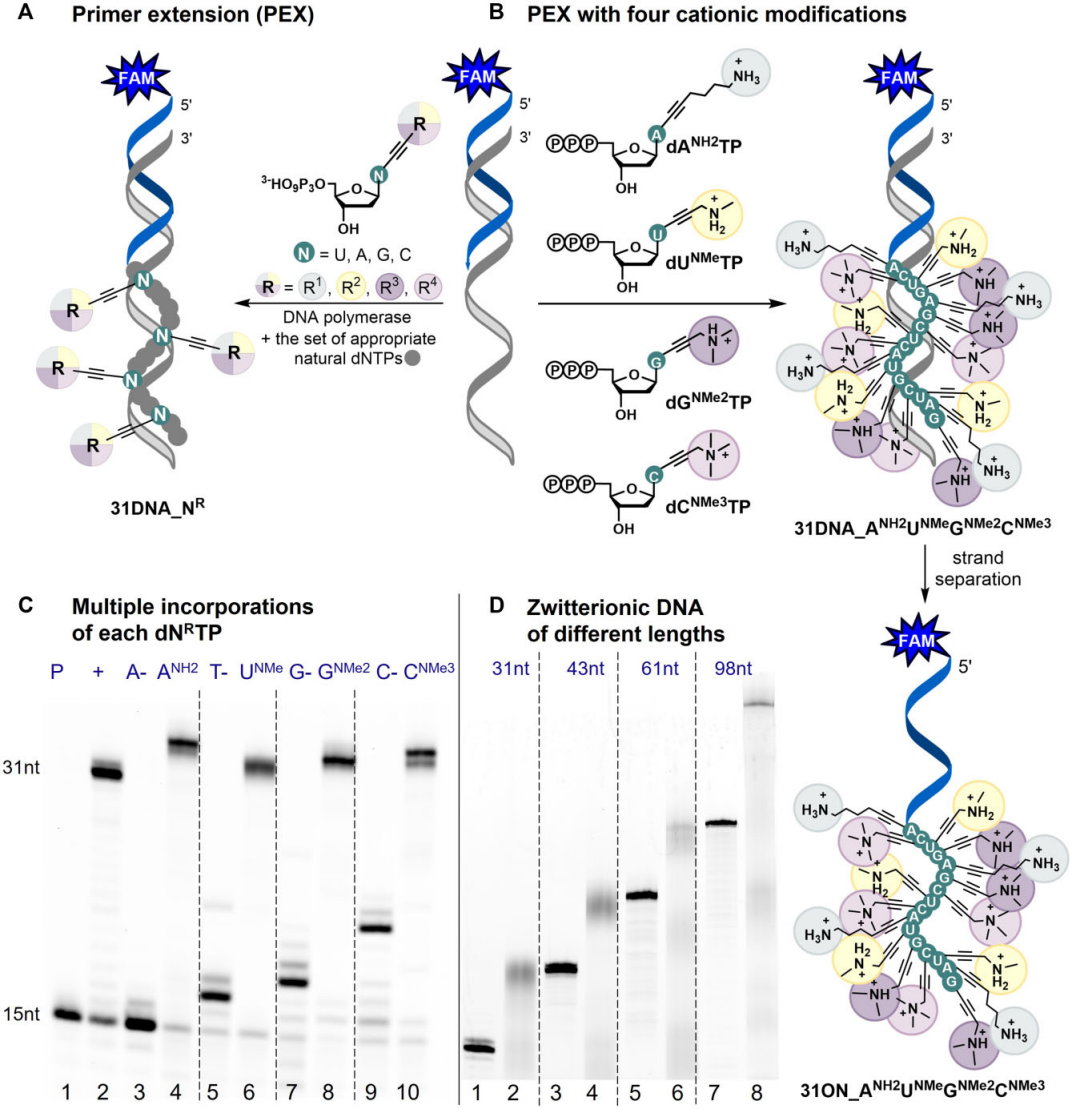
(**A**) PEX reaction using one modified **dN^R^TP** (R = NH2, NMe, NMe2, NMe3; N = A, U, G, C). (**B**) PEX reaction using all four modified **dN^R^TP**s followed by strand separation; (**C**) Denaturing PAGE analysis of PEX using KOD XL DNA polymerase, 5′-(6-FAM)-labeled 15-mer primer, and 31-mer template [lanes: (1) primer; (2) positive control; (4), (6), (8), (10) reactions containing either **dA^NH2^TP**, **dU^NMe^TP**, **dG^NMe2^TP** or **dC^NMe3^TP**; (3), (5), (7), (9) negative controls, in absence of either dATP, dTTP, dGTP or dCTP]. (**D**) Denaturing PAGE analysis of PEX using KOD XL DNA polymerase, 5′-(6-FAM)-labeled primer, set of four modified **dN^R^TP**s, and 31-mer (lane 2), 43-mer (lane 4), 61-mer (lane 6), and 98-mer (lane 8) templates; lanes 1, 3, 5, and 7: positive controls with natural dNTPs..

The reaction mixture (10 μL) contained 31-mer template Temp^Prb4basII^ (3 μM, 0.75 μL), 15-mer primer Prim^248short^-FAM (3 μM, 0.5 μL) (for sequences see Supplementary Table S1 in SI), **dA^NH2^TP** (0.25 mM, 1 μL), **dU^NMe^TP** (2 mM, 1 μL), **dG^NMe2^TP** (2 mM, 1 μL), **dC^NMe3^TP** (2 mM, 1 μL) (see structures in Scheme 1), KOD XL DNA polymerase (0.6 U), and the enzyme reaction buffer (10X, 1 μL). The reaction mixture was incubated for 30 min at 72°C, stopped by addition of PAGE stop solution (10 μL), and denatured for 5 min at 95°C. Sample was analyzed on 20% PAGE and visualized using fluorescence imaging (Fig. 1; Supplementary Fig. S3 in SI).

### Multiple incorporation of modified dN^R^TPs by PCR

The reaction mixture (10 μL) contained 98-mer template Temp^NK98^ (0.5 μM, 0.5 μL), reverse primer Prim^NK98pr1^-FAM and forward primer Prim^NK98pr2^-Cy5 (10 μM, 1 μL each) (for sequences see Supplementary Table S1 in SI), appropriate set of natural dNTPs (2 mM, 1 μL), one of the modified **dN^R^TP**s (R = NH2, NMe, NMe2, NMe3; N = A, U, G, C) (1 μL, conditions specified in Supplementary Table S9 in SI), suitable additives, KOD XL DNA polymerase, and a corresponding reaction buffer (10X, 1 μL). The positive control contained 0.1 U of KOD XL DNA polymerase and natural dNTPs (2 mM, 2 μL). All reaction mixtures were under cycling protocol: 94°C for 5 min, followed by 30 cycles at 94°C for 1 min, 53°C for 30 sec, and 72°C for 2 min, followed by a final elongation step at 72°C for 5 min. Samples were analyzed on 12.5% PAGE and agarose gel electrophoresis and visualized using fluorescence imaging (Fig. 3; Supplementary Fig. S8 in SI).

### Re-PCR of a fully modified template to natural DNA

**Scheme 2.**
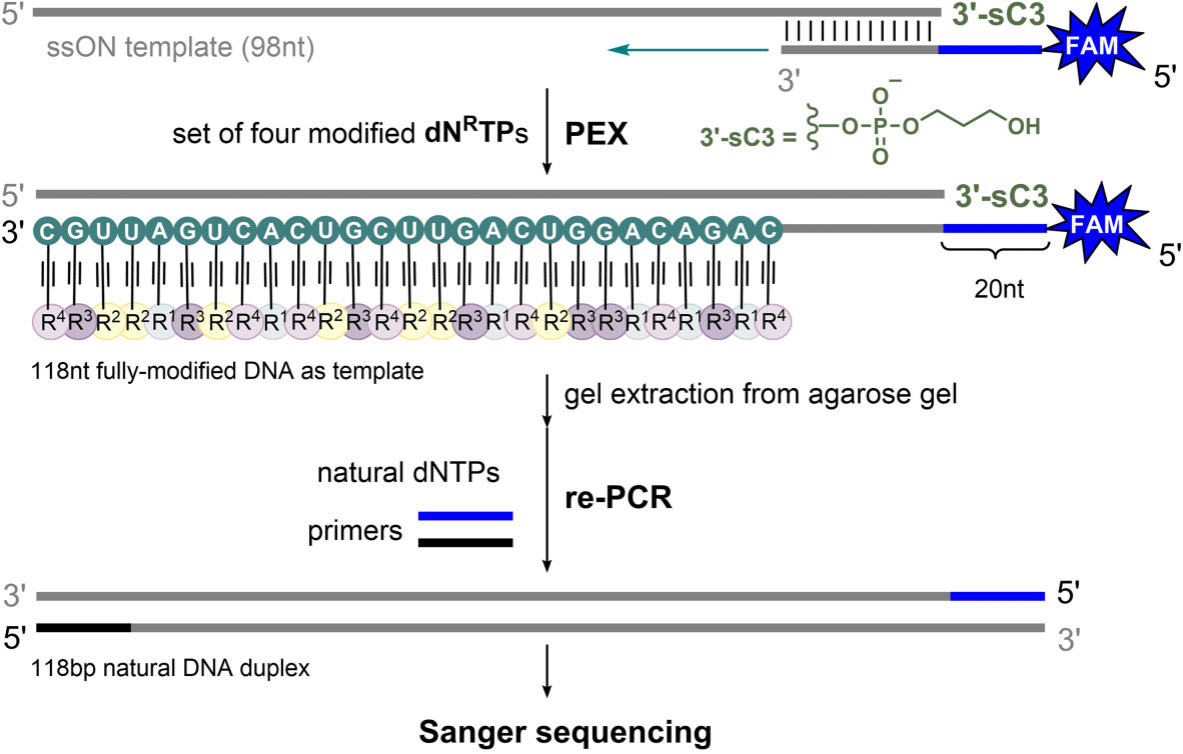
PEX reaction with an extended primer and a set of four modified **dN^R^TP**s followed by re-PCR with natural dNTPs to prepare dsDNA for Sanger sequencing.

To be able to sequence fully-modified ssONs after PEX, the following approach has been used (Scheme 2; Supplementary Scheme S3 in SI): modified **118DNA_A^NH2^U^NMe^G^NMe2^C^NMe3^** and **118DNA_A^NH2^U^EPh^G^PA^C^EAlk^** (for sequences see Supplementary Table S2 in SI) were obtained by PEX using 45nt 5′-(6-FAM)-labeled extended primer Prim^Flank-NK98pr1^-FAM and 98-mer template Temp^NK98^-sC3 modified at 3′-end with three carbon spacer (sC3) preventing any non-templated extension during PEX (see further in sections 2.15.2 and 2.15.3 in SI). Then the samples were loaded on 2% agarose gel (60 min, 125 V) (Supplementary Fig. S15 in SI) followed by gel extraction of the product-containing area using a plastic pipette tip. Tips containing gel fragments were soaked overnight in water, and then the extracted modified templates were used for re-PCR with primers Prim^Flank^-FAM and Prim^NK98pr2^-Cy5 and natural dNTPs (see section 2.15.4 and Supplementary Fig. S16 in SI).

### Preparation of 98DNA_dsA^NH2^U^EPh^G^PA^C^EAlk^ containing both strands fully modified with cationic, anionic, and hydrophobic modifications

Non-labeled oligonucleotides (ONs) **98ON_A^NH2^U^EPh^G^PA^C^EAlk^** and **98cON_A^NH2^U^EPh^G^PA^C^EAlk^** (preparation described below) were annealed together in Tris-HCl buffer (10 mM, 1 mM EDTA, 65 mM NaCl, pH 7.5) under following protocol: 95°C for 5 min, followed by gradual cooling to 25°C for 90 min. To prove the suitability of the conditions, annealing was additionally performed with 5′-(6-FAM)-labeled **98ON_A^NH2^U^EPh^G^PA^C^EAlk^** and 5′-Cy5-labeled **98cON_A^NH2^U^EPh^G^PA^C^EAlk^**. The labeled double-stranded product was further analyzed by 2% agarose gel electrophoresis and visualized using fluorescence imaging (Supplementary Fig. S17 in SI).

Preparation of **98ON_A^NH2^U^EPh^G^PA^C^EAlk^** and **98cON_A^NH2^U^EPh^G^PA^C^EAlk^**:

To obtain double-stranded **98DNA_A^NH2^U^EPh^G^PA^C^EAlk^** and **98cDNA_A^NH2^U^EPh^G^PA^C^EAlk^**, we prepared reaction mixtures (50 μL) that included dual-biotinylated templates and primers (100 μM, 3 μL each). For **98DNA_A^NH2^U^EPh^G^PA^C^EAlk^**, dual-biotinylated template Temp^NK98^-bio and primer Prim^NK98pr1^ were used, for **98cDNA_A^NH2^U^EPh^G^PA^C^EAlk^** – dual-biotinylated template Temp^NK98_comp^-bio and primer Prim^NK98pr2^ (for sequences, see Supplementary Tables S1 and S2 in SI). The mixtures also contained a set of four modified **dN^R^TP**s (R = NH2, EPh, PA, EAlk; N = A, U, G, C) (4 mM, 2.5 μL), KOD XL DNA polymerase (2.5 U), and the enzyme reaction buffer (10X, 5 μL). The reactions were incubated for 1 h at 60°C and then stopped by cooling to 8°C. The reactions were repeated eight times each to obtain sufficient DNA concentration. After that, the products were purified using Agencourt AMPure XP magnetic particles to remove shorter side-product ONs, and then modified strands were separated from the templates using Streptavidin magnetic particles (Roche) according to DBStv magnetoseparation procedure (section 2.7 in SI).

## Results and discussion

To explore the features of base-modified nucleotides bearing cationic functions, we designed and synthesized all four 2′-deoxyribonucleoside triphosphate derivatives (**dN^R^TP**s) each displaying a different form of amino function: primary, secondary and tertiary amino, as well as quaternary ammonium groups. The modifications were attached to position 5 of pyrimidines or 7 of 7-deazapurines through an alkyne linker, that is superior in dNTPs to be good substrates for DNA polymerases [41, 42]. The primary amino group was attached as 6-aminohex-1-ynyl group, which resembles a lysine side-chain, while the other cationic groups were introduced as methylamino-, dimethylamino-, or trimethylammonium-propargyl groups. Previous works on chemical synthesis of DNA containing 5-aminopropyl- or 5-aminopropargyluracil reported small bending of the B-DNA structure [43–46] and significant stabilization of DNA duplex indicating that the amino group is protonated in DNA and the pKa is increased due to electrostatic interactions with negative DNA backbone [47, 48] even in relatively weakly basic aminopropargyl group (propargylamine pKa 8.15)[48].

In the cases of **dA^NH2^TP**, **dU^NMe^TP**, and **dG^NMe2^TP**, synthesis was performed by Sonogashira coupling of functionalized alkynes **1a-c** with the corresponding iodinated 2′-deoxyribonucleoside triphosphates (**dN^I^TP**s) [37, 49, 38] (Scheme 1A). The reactions were carried out under argon in presence of Pd(PPh_3_)_4_ catalyst, CuI, and Et_3_N in anhydrous DMF for 5 h at room temperature. The products were purified by HPLC and isolated in moderate yields (27–55%) (for details see section 1.1 in SI; for copies of NMR spectra see section 6 in SI). However, we did not succeed in obtaining **dC^NMe3^TP** by direct coupling of propargyltrimethylammonium iodide with **dC^I^TP**. The reaction with such an electron-poor alkyne did not proceed under conditions suitable for triphosphates and would presumably require harsher conditions, potentially endangering the triphosphate group. In this case, we had to perform the Sonogashira coupling of the alkyne **1d** with 5-iodo-2′-deoxycytidine **dC^I^** (Scheme 1B). The coupling was performed under argon atmosphere using Pd(OAc)_2_ catalyst, TPPTS, CuI, and Et_3_N in anhydrous DMF for 2.5 h at 60°C (Scheme 1B). The resulting modified nucleoside **dC^NMe3^** was purified by HPLC and isolated as an orange-colored solid with 40% yield. In the next step, **dC^NMe3^** was converted into modified triphosphate **dC^NMe3^TP** using a standard triphosphorylation procedure [40]. The product was purified by HPLC and isolated with 6% yield (for details see section 1.2 in SI; for copies of NMR spectra see section 6 in SI).

Once synthesized, the modified triphosphates **dA^NH2^TP**, **dU^NMe^TP**, **dG^NMe2^TP**, **dC^NMe3^TP** were studied as substrates for two DNA polymerases: Vent(exo–) and KOD XL. The DNA polymerases were selected based on their previously reported ability to incorporate high density of modified nucleotides [21,23]. First, we have performed PEX experiments using 19-mer template and 15-mer primer encoding for one modified nucleotide (for detailed procedure see Supplementary Table S3, Supplementary Fig. S1, and section 2.1 in SI; for sequences see Supplementary Table S1 in SI; for UPLC-MS analysis results see Supplementary Table S8 and Supplementary Figs S21–S28 in SI). Then, we proceeded with multiple incorporation of each modified nucleotide using a 31-mer template encoding for four modified nucleotides (Fig. 1A; Supplementary Table S4 and section 2.2 in SI; for sequences see Supplementary Table S1 in SI). In all cases, full-length PEX products were formed. Figure 1C shows PAGE analysis of multiple incorporation PEX experiments using KOD XL DNA polymerase (also Supplementary Fig. S2A in SI). Similar results were observed with Vent(exo–) DNA polymerase, see Supplementary Fig. S2B in SI. The identity of the products was confirmed by UPLC-MS analysis of ONs obtained by a larger scale PEX with dual-biotinylated templates (with two biotins attached to the 5′-end for achieving stronger biotin-streptavidin interaction) followed by magnetoseparation purification with streptavidin-coated magnetic beads (for samples preparation details see section 2.7 and Supplementary Table S7 in SI; for UPLC-MS results see Supplementary Table S8 and Supplementary Figs S29–S36 in SI).

Next, we aimed to test different combinations of **dA^NH2^TP**, **dU^NMe^TP**, **dG^NMe2^TP**, and **dC^NMe3^TP** in PEX. For this, we used the previously mentioned 31-mer template, a 15-mer primer, KOD XL DNA polymerase, and various sets of two or three modified **dN^R^TP**s (always complemented with the remaining natural dNTPs) (for sequences, see Supplementary Table S1 in SI). In all cases, the formation of full-length products was observed (see section 2.3, Supplementary Table S5, and Supplementary Fig. S3 in SI). Further, we performed PEX with the same 31-mer template and all four modified **dN^R^TP**s (Fig. 1B). To characterize the product, the PEX reaction was repeated on a larger scale with a dual-biotinylated template followed by the template separation and the characterization of the fully-modified strand by MALDI-TOF analysis (for results see Supplementary Table S8 and Supplementary Fig. S37 in SI). The PAGE analysis (Fig. 1D; for the uncropped gel, see Supplementary Fig. S4 in SI) confirmed that the reaction proceeds with templates of different lengths (31, 43, 61, 98-mer) giving zwitterionic fully-modified PEX products containing 16–73 modified nucleotides in a row. Interestingly, while studying the template length limits, we observed that PEX with cationically modified **dN^R^TP**s is sequence-dependent. It appears that PEX is terminated when a template codes for two consecutive **dC^NMe3^MP** (for details see section 2.5, Supplementary Table S6, and Supplementary Fig. S6 in SI). Based on these observations, we designed the templates to ensure that the resulting modified ON contains at least one nucleotide between **dC^NMe3^MP**s (for sequences see Supplementary Table S1 and Supplementary Table S2 in SI). As seen in Fig. 1D, the zwitterionic hypermodified PEX products migrate significantly slower in the electric field than the natural analogs of the same size. It is understandable given that zwitterionic DNA is overall less charged and has a higher mass. The smearing of the bands can be explained by secondary structures or higher aggregate formation that cannot be resolved under the conditions applied in standard denaturing PAGE electrophoretic analysis, even using buffer with a higher pH (for details see Supplementary Fig. S5 in SI).

**Figure 2.**
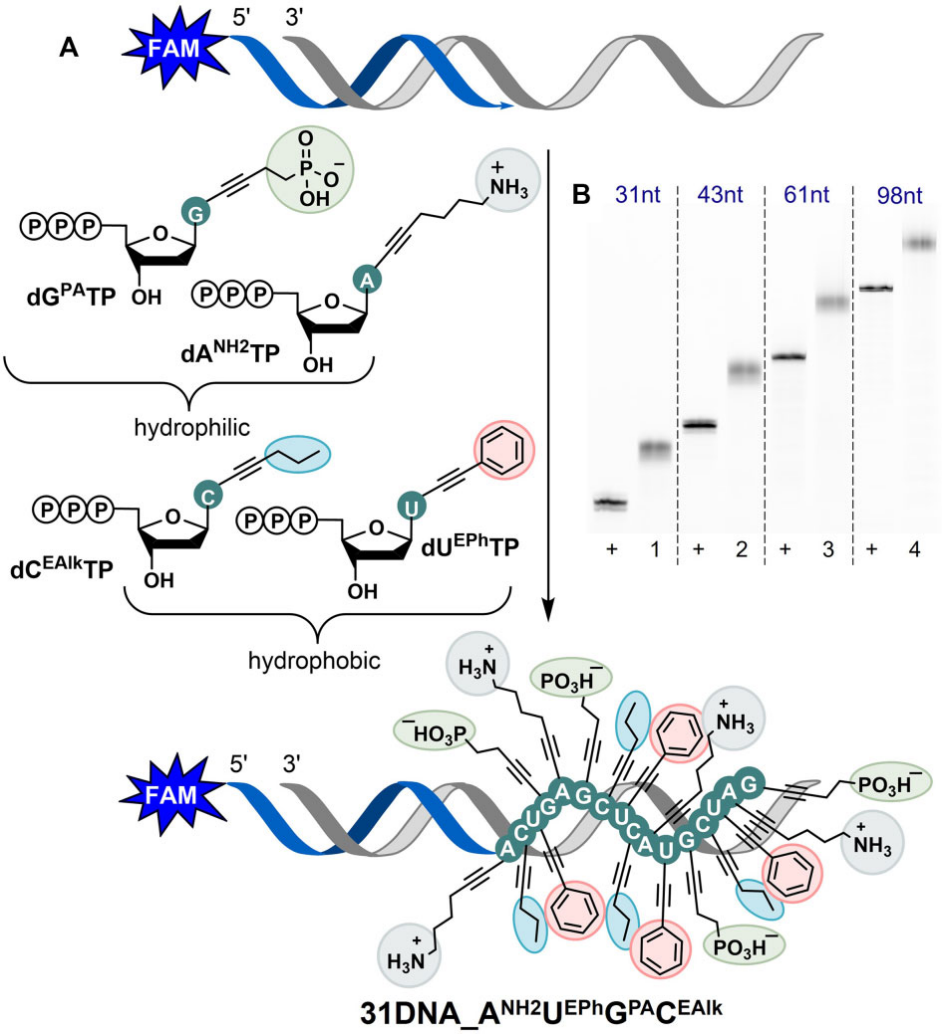
(**A**) PEX reaction using a set of four modified **dN^R^TP**s – **dA^NH2^TP**, **dU^EPh^TP**, **dG^PA^TP**, and **dC^EAlk^TP**. (**B**) Denaturing PAGE analysis of the PEX with KOD XL DNA polymerase, 5′-(6-FAM)-labeled primer, and 31-mer (lane 1), 43-mer (lane 2), 61-mer (lane 3), and 98-mer (lane 4) templates. (+): positive controls with natural dNTPs.

For potential applications, it is advantageous to be able to combine nucleotides bearing different types of modifications in PEX. In these studies, we tested whether the cationically modified nucleotides can be incorporated into DNA in combination with hydrophobic and anionic nucleotides. Thus, we synthesized previously reported anionic **dG^PA^TP** [23], hydrophobic **dU^EPh^TP**, and **dC^EAlk^TP** [21] and performed PEX reactions in combination with **dA^NH2^TP** (Fig. 2A). The reactions were carried out using templates of different lengths (31, 43, 61, and 98-mer) and KOD XL DNA polymerase (for sequences, see Supplementary Table S1 in SI; for the uncropped gel see Supplementary Fig. S7 in SI). The formation of full-length hypermodified ‘mixed’ PEX products was detected using PAGE gel electrophoresis (Fig. 2B). As in the case of zwitterionic DNA (Fig. 1D), the electrophoretic mobility of the hypermodified ‘mixed’ PEX products differed from the natural analogs, but the bands were much less smeared. Using a 5′-dual-biotinylated 31-mer template in a larger scale PEX, followed by magnetoseparation of the template with streptavidin-coated magnetic beads (for details see section 2.7 in SI), we successfully characterized the hypermodified 31-mer ON with a combination of cationic, anionic, and hydrophobic modifications by UPLC-MS analysis (Supplementary Table S8 and Supplementary Fig. S38, Supplementary Fig. S39 in SI).

**Figure 3.**
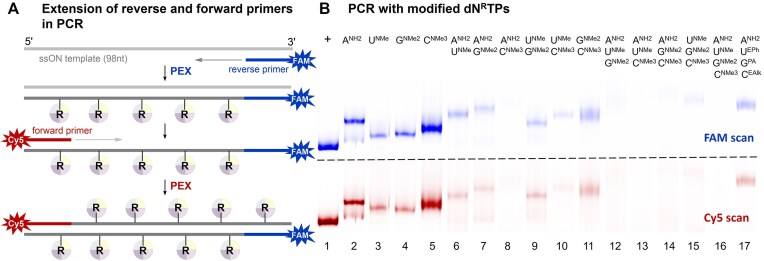
(**A**) Scheme of reverse and forward primers extension in PCR. (**B**) Denaturing PAGE analysis of PCR experiments with all **dN^R^TP** combinations using 5′-(6-FAM)-labeled reverse primer, 5′-Cy5-labeled forward primer, 98-mer template, and KOD XL DNA polymerase: lane (1) positive control; lanes (2–5) reactions using **dA^NH2^TP**, **dU^NMe^TP**, **dG^NMe2^TP** or **dC^NMe3^TP** in combination with the other three native dNTPs; lanes (6–16) reactions using various combinations of **dA^NH2^TP**, **dU^NMe^TP**, **dG^NMe2^TP**, and **dC^NMe3^TP** with an appropriate set of natural dNTPs (lane 16—no natural dNTPs); lane (17) PCR using **dA^NH2^TP**, **dG^PA^TP**, **dU^EPh^TP**, and **dC^EAlk^TP**.

After demonstrating that the modified cationic nucleotides could be incorporated into DNA by PEX, we moved forward with their application in the PCR. This task is particularly challenging because, in addition to incorporating a modified nucleotides into a DNA strand, a DNA polymerase must also effectively read through the modified template to synthesize another modified strand. Multiple cationic modifications attached to DNA can cause significant difficulties, such as unwanted interactions with DNA polymerase or facilitation of secondary structure formation, which may reduce the accessibility of the modified template. Relative difficulties with the use of aminopropargyl-dNTPs in PCR have been reported by others [35] previously. To determine if the modified nucleotides serve as proficient substrates in PCR, the experiments were designed in a particular way – with the reverse primer labeled with 6-FAM and the forward primer labeled with Cy5 (Fig. 3A). Such a design allows the extension of each primer to be followed independently. First, each of the cationic **dN^R^TP**s was studied in PCR reactions using KOD XL DNA polymerase, a single-stranded 98-mer template (for sequences see Supplementary Table S1 in SI), a set of remaining natural dNTPs, and various additives (for details, see section 2.9 and Supplementary Table S9 in SI). After 30 cycles, PCR products were analyzed on native agarose gel and denaturing PAGE using either FAM or Cy5 scan (Supplementary Fig. S8 in SI). Consequently, PCR proceeded with each cationic **dN^R^TP** giving full-length amplicon products with extension of both primers (Fig. 3B; for the unmodified and uncropped gel, see Supplementary Fig. S11 in SI).

Further, we conducted a systematic study of PCR reactions using various combinations of two, three, and all four **dN^R^TP**s with a single-stranded 98-mer template and KOD XL DNA polymerase (for sequences see Supplementary Table S1 and Supplementary Table S2 in SI; for details see Supplementary Table S10, Supplementary Table S11 and Supplementary Fig. S9, Supplementary Fig. S10 in SI). As demonstrated in Fig. 3B (lanes 6–11), full-length PCR products can be obtained for most combinations of two **dN^R^TP**s, although the yields are significantly lower compared to the cases with one modified **dN^R^TP**. Combinations of three **dN^R^TP**s in PCR (Fig. 3B, lanes 12–15) gave only trace amounts of the products, while the attempt to use all four cationic **dN^R^TP**s (Fig. 3B, lane 16) resulted in no product formation. In the latter case, we managed to obtain the product of reverse PEX through aPCR, which involves using an increased amount of template in the absence of the forward primer and provides only linear amplification (for conditions see Supplementary Table S12 and the PAGE analysis in Supplementary Fig. S12 in SI). Interestingly, the use of a combination of cationic **dA^NH2^TP**, anionic **dG^PA^TP**, and hydrophobic **dU^EPh^TP** and **dC^EAlk^TP** as substrates in PCR facilitated the extension of both primers and produced a full-length PCR product (Fig. 3B, lane 17).

**Figure 4.**
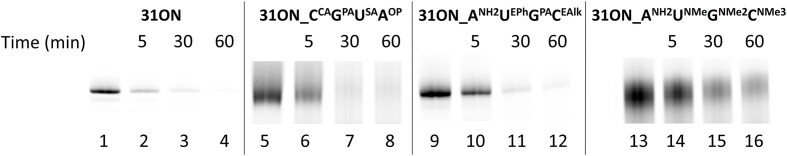
PAGE analysis of the stability of natural **31ON** (lanes 2–4), anionic **31ON_C^CA^G^PA^U^SA^A^OP^** (lanes 6–8), mixed **31ON_A^NH2^U^EPh^G^PA^C^EAlk^** (lanes 10–12), and zwitterionic **31ON_A^NH2^U^NMe^G^NMe2^C^NMe3^** (lanes 14–16) in the presence of 0.01U of DNase I for 5 min (lanes 2, 6, 10, 14), 30 min (lanes 3, 7, 11, 15), and 60 min (lanes 4, 8, 12, 16). Lanes 1, 5, 9, and 13 were not incubated with DNase I.

To investigate how the high density of cationic functional groups affects DNA stability toward nuclease degradation and compare with the effect of anionic groups [23], we prepared four 5′-(6-FAM)-labeled 31-mer ONs: natural **31ON**, fully modified zwitterionic **31ON_A^NH2^U^NMe^G^NMe2^C^NMe3^**, fully-modified mixed **31ON_A^NH2^U^EPh^G^PA^C^EAlk^** bearing cationic, anionic, and hydrophobic modifications (sample preparation is described in section 2.7 in SI; for sequences see Supplementary Table S2 in SI), and previously reported superanionic **31ON_C^CA^G^PA^U^SA^A^OP^** [23]. Although the results of nuclease degradation experiments with natural **31ON** and superanionic **31ON_C^CA^G^PA^U^SA^A^OP^** have been already described [23], we repeated the experiment with a new DNase I batch to be able to compare the results with those from **31ON_A^NH2^U^NMe^G^NMe2^C^NMe3^** and **31ON_A^NH2^U^EPh^G^PA^C^EAlk^**. The obtained ONs were incubated with DNase I for 5, 30, and 60 min at 37°C (for details, see section 2.13, Supplementary Table S13, and Supplementary Fig. S13 in SI). The experiments were performed in triplicate and the results were analyzed on PAGE and visualized using fluorescence imaging (Fig. 4). The average values of DNA recovery after incubation are given in Table 1. The results show that the cationic modifications improve ON stability toward DNase I. Furthermore, zwitterionic **31ON_A^NH2^U^NMe^G^NMe2^C^NMe3^** exhibits higher stability compared to superanionic **31ON_C^CA^G^PA^U^SA^A^OP^**, suggesting that cationic modifications enhance ON resistance more effectively than the anionic ones. The mixed **31ON_A^NH2^U^EPh^G^PA^C^EAlk^** containing cationic, anionic, and hydrophobic modifications was still significantly more stable towards degradation than natural **31ON**, but less stable than superanionic **31ON_C^CA^G^PA^U^SA^A^OP^**. To further demonstrate enhanced stability of the modified ONs in biological medium, the samples were incubated in human plasma. Zwitterionic **31ON_A^NH2^U^NMe^G^NMe2^C^NMe3^** and mixed **31ON_A^NH2^U^EPh^G^PA^C^EAlk^** have demonstrated enhanced stability in comparison to natural **31ON**, while superanionic **31ON_C^CA^G^PA^U^SA^A^OP^** supposedly formed aggregates with plasma content (for details see section 2.14 and Supplementary Fig. S14 in SI).

**Table 1.** Recovery (expressed in percent) of natural and hypermodified DNA after incubation with 0.01 U of DNase I

DNA title	Incubation time with DNase I (0.01 U)		
	5 min	30 min	60 min
**31ON**	25 ± 3%	6 ± 2%	1%
**31ON_C^CA^G^PA^U^SA^A^OP^**	60 ± 5%	22 ± 2%	15 ± 4%
**31ON_A^NH2^U^EPh^G^PA^C^EAlk^**	56 ± 7%	13 ± 4%	6 ± 2%
**31ON_A^NH2^U^NMe^G^NMe2^C^NMe3^**	79 ± 1%	41 ± 3%	24 ± 6%

While short ONs can be identified by mass spectrometry, longer fully-modified strands require a different method of characterization. Previously, we developed an approach for sequencing long hypermodified ONs obtained by aPCR, ensuring that the modified strand is sequenced rather than the unmodified template (Scheme 2) [21]. Here, we adapt this method for fully-modified zwitterionic ON obtained by PEX. The 5′-end of the FAM-labeled primer was extended with a 20-nucleotide flanking sequence, serving as a new primer region for re-PCR. Additionally, to prevent unwanted extension during PEX, the 98-mer template was modified at 3′-end with a three-carbon spacer (sC3) (for sequences see Supplementary Table S1 in SI). Then, the extended primer and the protected template were introduced into PEX reaction using KOD XL DNA polymerase and a set of four modified **dN^R^TP**s—either cationic **dA^NH2^TP**, **dU^NMe^TP**, **dG^NMe2^TP**, **dC^NMe3^TP**, or mixed **dA^NH2^TP**, **dU^EPh^TP**, **dG^PA^TP**, **dC^EAlk^TP**. Subsequently, the PEX products were loaded on 2% agarose gel followed by gel extraction of the product-containing area using a plastic pipette tip (for agarose gel see Supplementary Fig. S15 in SI). Tips containing gel fragments were soaked overnight in water, and then the extracted modified template was applied for re-PCR reaction using Pwo DNA polymerase (see section 2.15, Supplementary Scheme S3, and Supplementary Fig. S16 in SI). The re-PCR product was subjected to Sanger sequencing, which confirmed the fidelity of the hypermodified ON replication and the feasibility of accurately sequencing fully modified ONs (for samples preparation, see section 2.16 in SI; for results of Sanger sequencing see section 2.17 in SI).

Next, we decided to study the influence of the cationic modifications on properties and stability of DNA. For this purpose, we conducted temperature-dependent measurements on several hypermodified 98bp DNA duplexes. The first two samples **98DNA_A^NH2^U^NMe^G^NMe2^C^NMe3^** (**dA^NH2^TP**, **dU^NMe^TP**, **dG^NMe2^TP**, **dC^NMe3^TP** used as building blocks) and **98DNA_A^NH2^U^EPh^G^PA^C^EAlk^** (**dA^NH2^TP**, **dU^EPh^TP**, **dG^PA^TP**, **dC^EAlk^TP** used as building blocks) each consisted of one strand with 73 modified bases hybridized with a complementary non-modified strand. These samples were prepared using semipreparative PEX reactions, purified using Agencourt AMPure XP magnetic particles, concentrated, and dissolved in a buffer (10 mM Tris, 65 mM NaCl, 1 mM EDTA, pH 7.5). To prepare samples with both fully-modified strands, two complementary hypermodified ssONs should be obtained through PEX followed by template separation and, subsequently, annealed together. Two hypermodified ssONs **98ON_A^NH2^U^EPh^G^PA^C^EAlk^** and **98cON_A^NH2^U^EPh^G^PA^C^EAlk^** bearing cationic, anionic, and hydrophobic modifications were obtained and successfully annealed together in a buffer (10 mM Tris, 65 mM NaCl, 1 mM EDTA, pH 7.5) giving modified on both strands **98DNA_dsA^NH2^U^EPh^G^PA^C^EAlk^** (the procedure is described in Materials and Methods). However, attempts to anneal two fully-modified zwitterionic ssONs, **98ON_A^NH2^U^NMe^G^NMe2^C^NMe3^** and **98cON_A^NH2^U^NMe^G^NMe2^C^NMe3^**, were unsuccessful. It may be explained by the tendency of both ssONs to partially form higher-order aggregates. This assumption is supported by their unusual mobility on agarose gel, where a significant amount of the ssONs is retained in the well (Supplementary Fig. S18 in SI). To further verify the presence of aggregates, we performed confocal microscopy imaging of ssONs and dsDNA bearing different types of modifications. The results show that hypermodified ssONs (cationic, anionic and mixed) tend to form aggregates whereas the dsDNA do not (for details see section 4 and Supplementary Fig. S20 in SI). The last sample, an unmodified 98-bp **98DNA**, was obtained by PCR as a reference (for all procedures see section 3.1 in SI; for sequences see Supplementary Table S2 in SI).

Previous studies on the stability of base-modified DNA showed that the presence of protonated basic aminopropyl or aminopropargyl groups stabilizes DNA duplexes [47, 48]. We have also shown stabilization of duplexes by high density of alkynyl-linked nucleobases [50–52]. On the other hand, the presence of negatively charged modifications overbalance the stabilizing effect of ethynyl moieties and lower DNA duplex melting temperature (*T*_m_) [23]. It is reasonable to expect that introducing of high density of alkyne-linked cationic modifications into DNA should lead to an increase in *T*_m_. Indeed, for zwitterionic **98DNA_A^NH2^U^NMe^G^NMe2^C^NMe3^**, the *T*_m_ values increased from 82.9°C (as observed for natural **98DNA**) to 88.5°C (Table 2; Supplementary Fig. S19 in SI). Interestingly, significant duplex stabilization was also observed in the case of mixed **98DNA_A^NH2^U^EPh^G^PA^C^EAlk^** (*T*_m_ = 86.2°C), despite containing anionic modifications in this duplex. The most plausible explanation is that the π–π-stacking effect of alkynyl linkers, nucleobases, and phenyl modifications complemented by the effect of cationic groups, outweighed the destabilizing impact of the anionic modifications. The both-strand-hypermodified **98DNA_dsA^NH2^U^EPh^G^PA^C^EAlk^** containing cationic, anionic, and hydrophobic modifications was destabilized by 2.2°C compared to natural **98DNA**—an insignificant destabilization given the hypermodified nature of the duplex.

**Table 2.** Melting temperatures (*T*_m_) and hysteresis of natural and modified DNA determined by UV spectroscopy

DNA title	*T*_m_ (°C)	Hysteresis = *T*_m_–*T*_a_ (°C)	Δ*T*_m_ / modification
**98DNA**	82.9	2.7	–
**98DNA_A^NH2^U^NMe^G^NMe2^C^NMe3^**	88.5	3.1	+0.077
**98DNA_A^NH2^U^EPh^G^PA^C^EAlk^**	86.2	2.0	+0.049
**98DNA_dsA^NH2^U^EPh^G^PA^C^EAlk^**	80.7	7.5	–0.015

**Figure 5.**
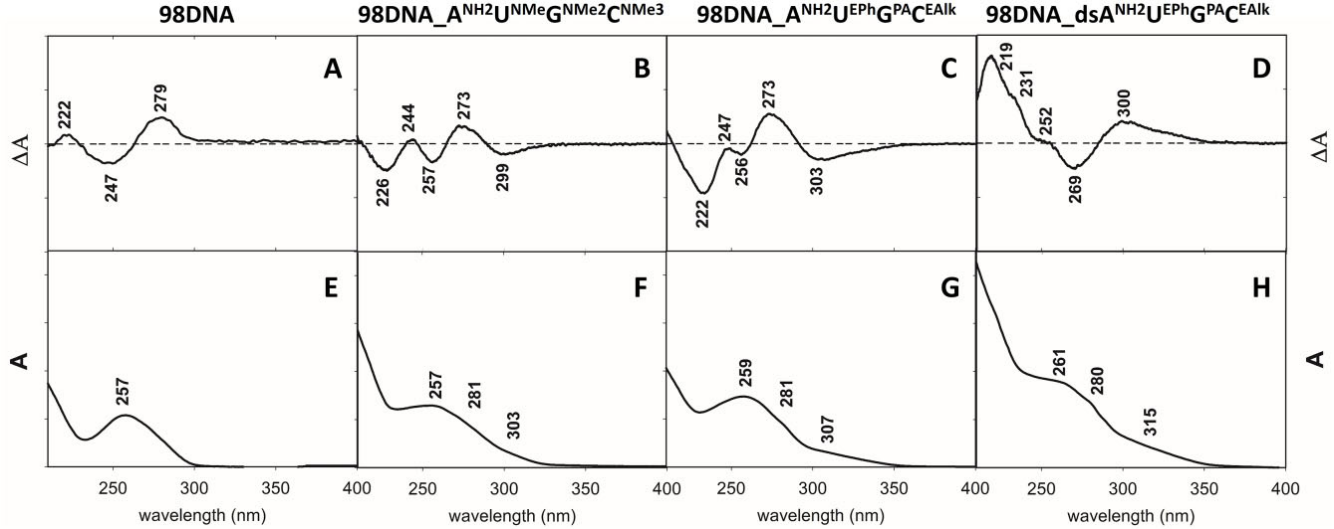
CD spectra (**A–D**) and UV absorption spectra (**E–H**) of **98DNA**, **98DNA_A^NH2^U^NMe^G^NMe2^C^NMe3^**, **98DNA_A^NH2^U^EPh^G^PA^C^EAlk^**, and **98DNA_dsA^NH2^U^EPh^G^PA^C^EAlk^**, respectively.

Although the incorporation of cationic residues into DNA was previously reported to only slightly bend the B-DNA structure [43–46], a high density of modifications can induce bigger structural changes. To gain some information about the structure and properties of the hypermodified DNA, we performed UV-vis absorption and circular dichroism (CD) spectroscopy studies. The natural **98DNA** was characterized by the absorption spectrum with maximum at ∼257 nm and by conservative CD spectrum with maxima at ∼247 nm (–) and ∼279 nm (+) typical for B-form structure of DNA (Fig. 5A and E). Introduction of the modifications into DNA duplexes caused spectral changes in both the absorption and CD spectra. The absorption spectra of the samples containing one hypermodified strand were characterized by a broader spectral band with a maximum at ∼260 nm (258 nm for **98DNA_A^NH2^U^NMe^G^NMe2^C^NMe3^** and 259 nm for **98DNA_A^NH2^U^EPh^G^PA^C^EAlk^**) and a shoulder at ∼305 nm (303 nm for **98DNA_A^NH2^U^NMe^G^NMe2^C^NMe3^** and 309 nm for **98DNA_A^NH2^U^EPh^G^PA^C^EAlk^**) (Fig. 5F and G), which is chracteristic for DNA bearing ethynyl-linked modifications [21, 23]. CD spectra of DNA containing one hypermodified strand hybridized with natural complementary strand, **98DNA_A^NH2^U^NMe^G^NMe2^C^NMe3^** and **98DNA_A^NH2^U^EPh^G^PA^C^EAlk^**, showed a positive CD spectral band at ∼273 nm (+) (Fig. 5B and C) For **98DNA_A^NH2^U^NMe^G^NMe2^C^NMe3^**, this band is accompanied by negative spectral bands at ∼299 nm (–), ∼257 nm (–), ∼226 nm (–), as well as a positive spectral band at ∼244 nm (+). A similar spectral pattern was observed for **98DNA_A^NH2^U^EPh^G^PA^C^EAlk^** with a positive band at ∼273 nm (+) and negative bands at ∼303 nm (–), ∼256 nm (–), and ∼222 nm (–). These results suggest that they may still adopt B-DNA (or similar) conformation. In the both-strands-hypermodified **98DNA_dsA^NH2^U^EPh^G^PA^C^EAlk^** with cationic, anionic, and hydrophobic groups, the UV-vis spectrum was quite similar to those of one-strand-hypermodified (Fig. 5H), but on the other hand, the CD spectrum of **98DNA_dsA^NH2^U^EPh^G^PA^C^EAlk^** displayed significant differences. It was characterized by following spectral bands: 300 nm (+), 269 nm (–), 252 nm (+), 231 nm (+), and 219 nm (+). The CD spectral pattern of **98DNA_dsA^NH2^U^EPh^G^PA^C^EAlk^** also differed significantly from the spectra of both-strand-superanionic DNA described previously [23]. This may indicate some conformational changes but we cannot make any reliable conclusion about the possible structure solely based on the changes in CD spectra.

## Conclusions

We designed and synthesized a full set of four **dN^R^TP**s modified with various cationic groups attached through an alkyne tether to the C5 position of pyrimidines or to the C7 position of 7-deazapurines. All of them were shown to be efficient substrates for Vent(exo–) and KOD XL DNA polymerases in PEX experiments. Using KOD XL DNA polymerase it was possible to incorporate into DNA all combinations of two, three, and even four modified nucleotides with up to 73 cationic modifications in a row. Additionally, a combination of one cationic (**dA^NH2^TP**), previously reported anionic [23] (**dG^PA^TP**) and two hydrophobic [21] (**dU^EPh^TP** and **dC^EAlk^TP**) modified **dN^R^TP**s was successfully applied in PEX resulting in mixed hypermodified DNA bearing a wide range of functional groups. In PCR, the exponential amplification through extension of both forward and reverse primers proceeded with each modified nucleotide separately (in combination with three natural dNTPs), while combinations of two, three, and four cationic modified **dN^R^TP**s gave either very low amplification or no PCR product. Interestingly, the combination of cationic (**dA^NH2^TP**), anionic (**dG^PA^TP**), and hydrophobic (**dU^EPh^TP** and **dC^EAlk^TP**) modified **dN^R^TP**s yielded a full-length PCR product with extension of both primers. In the case of fully-modified zwitterionic DNA, only linear amplification (aPCR) was observed, indicating that the zwitterionic DNA strand is not a good template for the enzymatic synthesis of another hypermodified strand. Yet, the zwitterionic DNA strand is an efficient enough template to be replicated with natural dNTPs with high fidelity, allowing for re-PCR and further sequencing. Although the alkyne-linked cationic modified dNTPs worked as substrates for polymerases, they were significantly less efficient compared to alkyne-linked hydrophobic or anionic dNTPs, which might be caused by some repulsive interactions with cationic arginine residues that occur in the active site of DNA polymerases [32,53].

The results of the UV, CD, and hybridization studies showed that incorporation of cationic modifications into one DNA strand increases duplex stability. Even in the case of mixed hypermodified DNA, containing only one cationic modification among others, the stabilizing effect was significant. The DNA duplex with both strands fully-modified with cationic, anionic, and hydrophobic modifications exhibited a slight decrease in *T*_m_ values; nevertheless, it still posesses impressively high stability for such hypermodified DNA. The CD spectra of the duplexes with one fully-modified strand show resembling patterns to natural B-DNA, while mixed DNA with both strands hypermodified shows significant differences.

These results widen the knowledge about the scope and limitations of the enzymatic synthesis and hybridization properties of hypermodified ONs. The cationic amino- or ammonium-linked dNTPs are somewhat worse substrates for polymerases compared to their neutral or anionic analogues, but still they can be used for polymerase syntheses of modified or hypermodified ONs bearing a combination of four different cationic modifications. These hypermodified ONs have zwitterionic character where the anionic phosphate backbone is compensated by the cationic substituents. The unique properties of such zwitterionic ONs and increased hybridization should encourage further research leading into diverse applications including development or delivery of therapeutic ONs, assembly of functionalized DNA nanostructures, development of functional polymers and materials etc. The dNTPs bearing the cationic modifications can be combined with other modified nucleotides, bearing hydrophobic, anionic, sugar-linked or other modifications for selection of functional nucleic acids including aptamers or DNAzymes.

## Supplementary Material

gkaf155_Supplemental_File

## Data Availability

The data underlying this article are available in the article and in its online supplementary material. The supplementary information includes additional figures and schemes, additional gels and uncropped gels from Figs. 1–3, complete experimental part and copies of NMR and MALDI spectra.
