# Supplementary material for: Zwitterionic DNA: enzymatic synthesis of hypermodified DNA bearing four different cationic substituents at all four nucleobases
Source: Nucleic Acids Res. 2025 Mar 8;53(5):gkaf155. doi: 10.1093/nar/gkaf155 (PMC11890062; doi:10.1093/nar/gkaf155)
Supplement: gkaf155_Supplemental_File [file gkaf155_supplemental_file.pdf]

Supporting Information

Zwitterionic DNA. Enzymatic Synthesis of Hypermodified DNA Bearing Four Different Cationic Substituents at all Four Nucleobases

Natalia Kuprikova,[a,b] Marek Ondruš,[a] Lucie Bednárová,[a] Tomáš Kraus,[a] Lenka Poštová Slavětínská,[a] Veronika Sýkorová,[a] Michal Hocek* [a,b]

a) Institute of Organic Chemistry and Biochemistry, Czech Academy of Sciences, Flemingovo nam. 2, CZ-16000 Prague 6, Czech Republic; hocek@uochb.cas.cz

b) Department of Organic Chemistry, Faculty of Science, Charles University, Hlavova 8, CZ-12843 Prague 2, Czech Republic

Table of Contents

1. Experimental section – organic chemistry 4

1.1. Synthesis of modified triphosphates **dN^R^TPs** (N = U, A, G) 5

1.2. Synthesis of modified 2'-deoxycytidine 5'-*O*-triphosphate **dC^NMe3^TP** 8

2. Experimental section – biochemistry 11

2.1. PEX – Single incorporation (one modified **dN^R^TP**) 20

2.2. PEX – Multiple incorporation (one modified **dN^R^TP**) 22

2.3. PEX – Multiple incorporation (two, three, and four modified **dN^R^TPs**) 23

2.4. PEX – Multiple incorporation (**dA^NH2^TP**, **dU^NMe^TP**, **dG^NMe2^TP**, **dC^NMe3^TP** in various template length) 25

2.5. PEX with 98-mer template containing nucleotide repetitions 28

2.6. PEX – Multiple incorporation (**dA^NH2^TP**, **dU^EPh^TP**, **dG^PA^TP**, **dC^EAlk^TP** in various template length) 29

2.7. Sample preparation for mass spectrometry analysis 31

2.8. UHPLC-MS and MALDI-TOF measurements 33

2.9. PCR – Multiple incorporation (one modified **dN^R^TP**) 33

2.10. PCR – Multiple incorporation (two modified **dN^R^TPs**) 36

2.11. PCR – Multiple incorporation (three and four modified **dN^R^TPs**) 37

2.12. aPCR – Multiple incorporation (four modified **dN^R^TPs**) 40

2.13. Nuclease degradation experiments 42

2.14. ONs stability in human plasma 44

2.15. Application of fully-modified ssONs for sequencing 45

2.15.1. Re-PCR of fully-modified ssONs obtained by PEX 45

2.15.2. PEX – synthesis of **118DNA_A^NH2^U^NMe^G^NMe2^C^NMe3^** 46

2.15.3. PEX – synthesis of **118DNA_A^NH2^U^EPh^G^PA^C^EAlk^** 47

2.15.4. Re-PCR – synthesis of **118PCR_natural** (for further sequencing) 47

2.16. Sanger sequencing 49

2.17. Results of Sanger sequencing 50

2.17.1. **118PCR_natural_1** 50

2.17.2. **118PCR_natural_2** 51

3. Experimental section – CD spectroscopy and melting temperatures determination 52

3.1. Preparation of the samples 52

3.1.1. Preparation of **98DNA** 52

3.1.2. Preparation of **98DNA_A^{NH2}U^{NMe}G^{NMe2}C^{NMe3}** 52
3.1.3. Preparation of **98DNA_A^{NH2}U^{EPh}G^{PA}C^{EAlk}** 53
3.1.4. Preparation of **98DNA_dsA^{NH2}U^{EPh}G^{PA}C^{EAlk}** 53
3.1.5. Preparation of **98DNA_dsA^{NH2}U^{NMe}G^{NMe2}C^{NMe3}** (unsuccessful) 55
3.2. UV-vis spectroscopy 56
4. Experimental section – microscopy 56
4.1. Preparation of double-stranded samples 57
4.2. Preparation of single-stranded samples 57
4.3. Confocal microscopy imaging 57
5. Copies of ESI spectra of modified oligonucleotides 59
6. Copies of NMR spectra 69
6.1. ^{1}H, ^{13}C and ^{31}P{^{1}H}NMR spectra of **dA^{NH2}TP** 69
6.2. ^{1}H, ^{13}C and ^{31}P{^{1}H}NMR spectra of **dU^{NMe}TP** 70
6.3. ^{1}H, ^{13}C and ^{31}P{^{1}H}NMR spectra of **dG^{NMe2}TP** 72
6.4. ^{1}H, ^{13}C and ^{31}P{^{1}H}NMR spectra of **dC^{NMe3}** 73
6.5. ^{1}H, ^{13}C and ^{31}P{^{1}H}NMR spectra of **dC^{NMe3}TP** 74
7. HPLC traces of modified dNTPs 76
7.1. HPLC traces of **dA^{NH2}TP** 76
7.2. HPLC traces of **dU^{NMe}TP** 77
7.3. HPLC traces of **dG^{NMe2}TP** 79
7.4. HPLC traces of **dC^{NMe3}** 80
7.5. HPLC traces of **dC^{NMe3}TP** 81
8. References 83

1. Experimental section – organic chemistry

General remarks

All solvents and reagents were purchased from commercial suppliers and used as received. NMR spectra were recorded on Bruker Avance 400 MHz (400.0 MHz for ^{1}H, 162 MHz for ^{31}P, 100 MHz for ^{13}C) and Bruker Avance 500 MHz (500 MHz for ^{1}H, 125.7 MHz for ^{13}C, 202.3 for ^{31}P) spectrometers from sample solutions in D_2O. Chemical shifts (in ppm, δ scale) were referenced as follows: D_2O (referenced to t-BuOH as an external reference; 1.25 ppm for ^{1}H NMR and 31.6 ppm for ^{13}C NMR). ^{31}P chemical shifts were referenced to H_3PO_4 as an external reference. Chemical shifts are given in ppm (δ scale), coupling constants (J) in Hz. Low- and high-resolution mass spectra were measured on LTQ Orbitrap XL spectrometer (ESI ionization, Thermo Fisher Scientific). All mass spectra were acquired by the MS service at IOCB. Purification of the obtained compounds was performed using HPLC (Waters modular HPLC system) on columns Phenomenex Kinetex EVO C18 (Kinetex® 5 µm EVO C18 100 Å, AXIA Packed LC Column 250 x 21.2 mm) or Waters X-Bridge Shield RP18 (XBridge BEH Shield RP18 OBD Prep Column, 130Å, 5 µm, 19 x 150 mm) and columns packed with DEAE Sepharose Fast Flow ion-exchange resin (Cytiva). Buffer A (0.1 M TEAB in H2O) and buffer B (0.1 M TEAB in 50% MeOH) were used for purification of nucleoside triphosphates on RP-HPLC columns. Reactions were monitored by thin layer chromatography (TLC) on TLC silica gel 60 F254 and TLC silica gel 60 RF-18 F254s (Merck Life Science) and detected by UV (254 nm) and by Advion Expression Compact Mass spectrometer connected with Plate Express® TLC Plate Reader using electrospray ionization (ESI). Reactions with triphosphates were analyzed by normal phase TLC using IPAV (isopropyl alcohol /NH_4OH /H_2O, ratio 11/7/2) as mobile phase or reverse phase TLC using MeOH/H_2O 1/1 as mobile phase.

Chemicals were of analytical grade. 3-Dimethylamino-1-propyne, N-Methylpropargylamine, were purchased from Merck Life Science. Propargylamine, hex-5-yn-1-amine hydrochloride, 5-iodo-2'-deoxyuridine, and 5-iodo-2'-deoxycytidine were purchased from Fluorochem Ltd. Synthesis and characterization data for 7-iodo-2’-deoxy-7-deazaadenosine 5’-*O*-triphosphate (dA^{I}TP)[1], 5-iodo-2’-deoxyuridine 5’-*O*-triphosphate (dU^{I}TP)[2], 7-iodo-2’-deoxy-7-deazaguanosine 5’-*O*-triphosphate (dG^{I}TP)[3], propargyltrimethylammonium iodide[4] were reported previously.

1.1. Synthesis of modified triphosphates dN^{R}TPs (N = U, A, G)

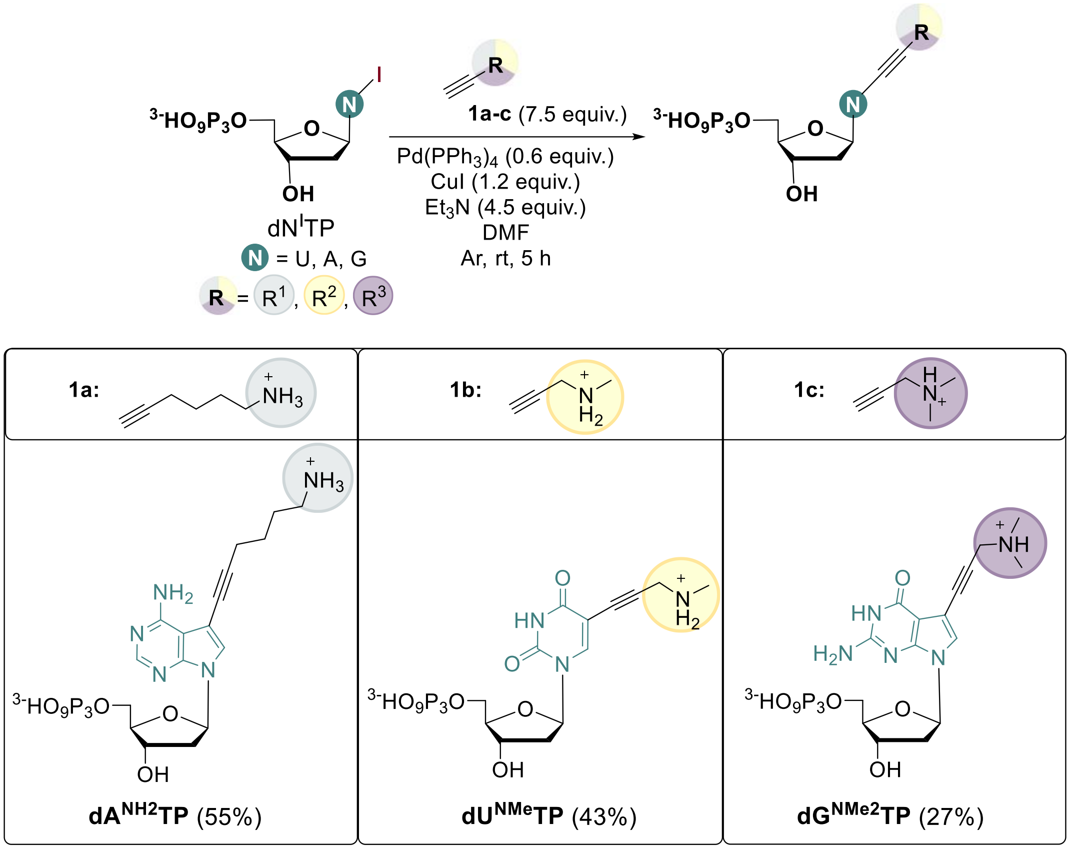

Scheme S1. Reaction scheme of **dN^{R}TP** synthesis (N = U, A, G) described by Method A.

Method A. Anhydrous DMF followed by Et_3N (4.5 equiv.) were added through a septum to an argon-purged flask charged with a halogenated nucleoside triphosphate dN^{I}TP (N = U, A, G) (1 equiv.), a corresponding alkyne **1a–c** (7.5 equiv.), CuI (1.2 equiv.), and $Pd(PPh_3)_4$ (0.6 equiv.). The mixture was stirred at room temperature for 5 hours under argon atmosphere. After 5 hours, the solvent was evaporated under vacuum. The reaction was monitored by reverse phase TLC using MeOH/H_2O 1/1 as mobile phase. The purification was done by reverse-phase HPLC with a linear gradient of MeOH in 0.1 M triethylammonium bicarbonate (TEAB) buffer (pH 7.6). In the case of **dU^{NMe}TP**, an additional purification using a Sepharose FF column with a linear gradient of 800 mM TEAB solution in H_2O (0–100%) was required.

7-(6-aminohex-1-yn-1-yl)-2'-deoxy-7-deazaadenosine 5'-*O*-triphosphate (dA^{NH2}TP)

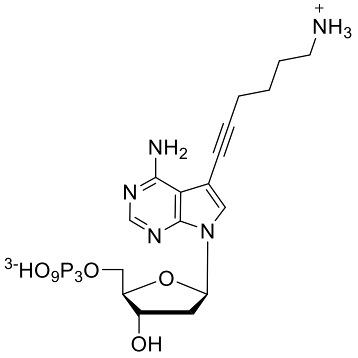

The compound **dA^{NH2}TP** was prepared from dA^{I}TP (62.3 mg, 0.068 mmol) by Method A as described above. The product was purified by reverse-phase HPLC with a linear gradient 10 to 100% of buffer B in buffer A (for details see section 7.1). The pure product isolated as a colorless solid (29.6 mg, 0.038 mmol; 55% yield); ^{1}H NMR (500.0 MHz, D_2O): 1.70 (m, 2H, C**H**$_2$CH$_2$CH$_2$NH$_2$); 1.92 (m, 2H, CH$_2$C**H**$_2$CH$_2$NH$_2$); 2.50 (ddd, 1H, J_{gem} = 13.9 Hz, $J_{2'a,1}$ = 6.4 Hz, $J_{2'a,3'}$ = 3.5 Hz, H-2´a); 2.56 (t, 2H, $J_{CH2,CH2}$ = 6.4 Hz, C≡CCH$_2$); 2.62 (ddd, 1H, J_{gem} = 13.9 Hz, $J_{2'b,1'}$ = 7.4 Hz, $J_{2'b,3'}$ = 6.0 Hz, H-2´b); 3.08 (m, 2H, CH$_2$CH$_2$C**H**$_2$NH$_2$); 4.17 (m, 1H, H-5´a); 4.20 – 4.27 (m, 2H, H-5´b,4´); 4.78 (m, 1H, H-3´); 6.59 (bt, 1H, $J_{1',2'b}$ = $J_{1',2'a}$ = 6.9 Hz, H-1´); 7.71 (s, 1H, H-8); 8.11 (s, 1H, H-2).

^{13}C NMR (125.7 MHz, D_2O): 20.38 (C≡C**C**H$_2$); 26.46 (**C**H$_2$CH$_2$CH$_2$NH$_2$); 27.98 (CH$_2$**C**H$_2$CH$_2$NH$_2$); 41.04 (CH$_2$CH$_2$**C**H$_2$NH$_2$); 41.46 (CH$_2$-2´); 67.44 (d, $J_{C,P}$ = 5.7 Hz, CH$_2$-5´); 73.24 (CH-3´); 75.35 (**C**≡CCH$_2$); 85.25 (CH-1´); 87.44 (d, $J_{C,P}$ = 9.2 Hz, CH-4´); 95.14 (C≡**C**CH$_2$); 99.17 (C-7); 104.36 (C-5); 128.22 (CH-8); 149.82 (C-4); 152.87 (CH-2); 158.39 (C-6).

^{31}P NMR (202.4 MHz, D_2O): -21.89 (t, 1P, $J_{\beta,\alpha}$ = $J_{\beta,\gamma}$ = 19.9 Hz, P_β); -10.50 (d, 1P, $J_{\alpha,\beta}$ = 19.7 Hz, P_α); -8.43 (d, 1P, $J_{\gamma,\beta}$ = 20.1 Hz, P_γ).

HR MS (ESI–) for $C_{17}H_{25}O_{12}N_5P_3$ [M – H]$^-$ calcd.: 584.07125 found: 584.07107.

5-(3-methylaminoprop-1-yn-1-yl)-2'-deoxyuridine 5'-*O*-triphosphate (dU^{NMe}TP)

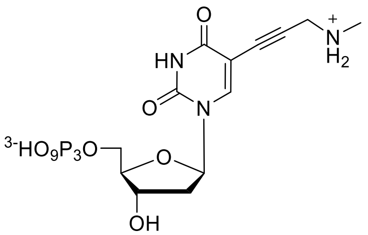

The compound **dU^{NMe}TP** was prepared from dU^{I}TP (30 mg, 0.033 mmol) by Method A as described above. The product was purified by reverse-phase HPLC with a linear gradient 0 to 100% of buffer B in buffer A. The appropriate fractions were collected, evaporated, and repurified by ion-exchange HPLC (0 to 100% of 800 mM TEAB in H_2O, for details see section 7.2). The pure product was isolated as a colorless solid (10.5 mg, 0.014 mmol; 43% yield); ^{1}H NMR (500.0 MHz, D_2O): 2.40 (dt, 1H, J_{gem} = 14.1 Hz, $J_{2´a,1´}$ = $J_{2´a,3´}$ = 6.1 Hz, H-2´a); 2.47 (ddd, 1H, J_{gem} = 14.1 Hz, $J_{2´a,1´}$ = 6.4 Hz, $J_{2´a,3´}$ = 5.2 Hz, H-2´b); 2.80 (s, 3H, CH_3NH); 4.04 and 4.09 (2×d, 2×1H, J_{gem} = 16.6 Hz, C≡CCH_2); 4.20 (m, 1H; H-4´); 4.26 (ddd, 1H, J_{gem} = 11.9 Hz, $J_{5´a,P}$ = 5.3 Hz, $J_{5´a,4´}$ = 2.4 Hz, 5´a); 4.31 (ddd, 1H, J_{gem} = 11.9 Hz, $J_{5´b,P}$ = 3.8 Hz, $J_{5´b,4´}$ = 2.6 Hz, 5´a); 4.67 (m, 1H, H-3´); 6.30 (t, 1H, $J_{1´,2´a}$ = $J_{1´,2´b}$ = 6.1 Hz, H-1´); 8.49 (s, 1H, H-6).

^{13}C NMR (125.7 MHz, D_2O): 34.61 (CH_3NH); 40.60 (C≡**C**CH_2); 41.57 (CH_2-2´); 66.70 (d, $J_{C,P}$ = 5.5 Hz, CH_2-5´); 71.48 (CH-3´); 81.24 (**C**≡CCH_2); 85.70 (C≡**C**CH_2); 87.80 (d, $J_{C,P}$ = 9.3 Hz, CH-4´); 87.81 (CH-1´); 100.02 (C-5); 148.68 (CH-6); 152.63 (C-2); 166.53 (C-4).

^{31}P NMR (202.4 MHz, D_2O): -21.65 (t, 1P, $J_{\beta,\alpha}$ = $J_{\beta,\gamma}$ = 19.6 Hz, P_β); -10.62 (d, 1P, $J_{\alpha,\beta}$ = 19.6 Hz, P_α); -7.44 (bs, 1P, P_γ).

HR MS (ESI–) for $C_{13}H_{19}O_{14}N_3P_3$ $[M – H]^–$ calcd.: 534.00798 found: 534.00848.

7-(3-dimethylaminoprop-1-yn-1-yl)-2’-deoxy-7-deazaguanosine 5’-*O*-triphosphate (dG^{NMe2}TP)

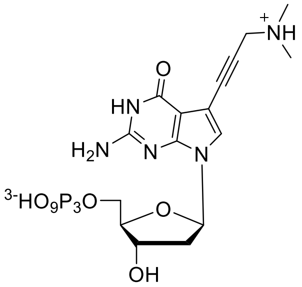

The compound **dG^{NMe2}TP** was prepared from dG^{I}TP (44.8 mg, 0.048 mmol) by Method A as described above. The product was purified by reverse-phase HPLC with a linear gradient 10 to 100% of buffer B in buffer A (for details see section 7.3). The pure product was isolated as a colorless solid (10.3 mg, 0.013 mmol; 27% yield); ^{1}H NMR (500 MHz, D_2O): 2.43 (ddd, 1H, J_{gem} = 14.0 Hz, $J_{2´a,1´}$ = 6.3 Hz, $J_{2´a,3´}$ = 3.3 Hz, H-2´a); 2.63 (ddd, 1H, J_{gem} = 14.0 Hz, $J_{2´b,1´}$ = 7.7 Hz, $J_{2´b,3´}$ = 6.1 Hz, H-2´b); 3.01 (s, 6H, $(CH_3)_2N$); 4.13 (dt, 1H, J_{gem} = 12.2 Hz, $J_{5´a,P}$ = $J_{5´a,4´}$ = 4.5 Hz, H-5´a); 4.18 – 4.25 (m, 2H, H-5´b,4´); 4.21 (s, 2H, C≡CCH_2); 6.46 (dd, 1H,

$J_{1',2'b}$ = 7.7 Hz, $J_{1',2'a}$ = 6.3 Hz, H-1´); 7.61 (s, 1H, H-8). *The signal of hydrogen H-3´ was overlapped by the signal of* H_2O.

^{13}C NMR (125.7 MHz, D_2O): 40.92 (CH_2-2´); 44.18 ($(CH_3)_2N$); 50.06 (C≡C**C**H_2); 67.43 (d, $J_{C,P}$ = 5.3 Hz, CH_2-5´); 73.31 (CH-3´); 81.41 and 84.93 (**C**≡**C**CH_2); 85.22 (CH-1´); 87.47 (d, $J_{C,P}$ = 9.2 Hz, CH-4´); 99.29 (C-7); 101.89 (C-5); 127.52 (CH-8); 152.94 (C-4); 155.43 (C-2); 162.89 (C-6).

^{31}P NMR (202.4 MHz, D_2O): -22.16 (t, 1P, $J_{\beta,\alpha}$ = $J_{\beta,\gamma}$ = 19.7 Hz, P_β); -10.50 (d, 1P, $J_{\alpha,\beta}$ = 19.8 Hz, P_α); -7.90 (bs, 1P, P_γ).

HR MS (ESI–) for $C_{16}H_{23}O_{13}N_5P_3$ $[M-H]^-$ calcd.: 586.05052 found: 586.05017.

1.2. Synthesis of modified 2'-deoxycytidine 5'-*O*-triphosphate dC^{NMe3}TP

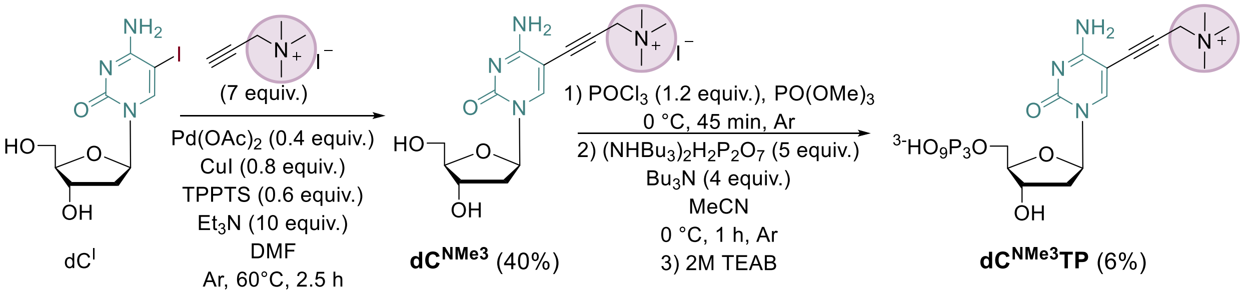

Scheme S2. Reaction scheme of **dC^{NMe3}TP** synthesis.

5-(3-trimethylaminoprop-1-yn-1-yl)-2'-deoxycytidine iodide (dC^{NMe3})

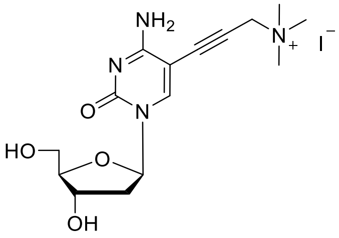

Anhydrous DMF followed by Et_3N (0.4 mL, 2.83 mmol) was added through a septum to an argon-purged flask charged with 5-iodo-2'-deoxycytidine dC^{I} (100 mg, 0.28 mmol), propargyltrimethylammonium iodide (446.2 mg, 1.98 mmol), CuI (43.2 mg, 0.23 mmol), TPPTS (96.61 mg, 0.17 mmol), and $Pd(OAc)_2$ (25.4 mg, 0.11 mmol). The mixture was stirred for 2.5 h at 60 °C under argon atmosphere. Then, the solvent was evaporated under vacuum. The reaction was monitored by normal phase TLC using DCM/MeOH 2/1 as mobile phase. The product was purified

by reverse-phase HPLC with a linear gradient of MeOH (for details see section 7.4) and isolated as an orange-colored solid (50.8 mg, 0.11 mmol; 40% yield).

^1H NMR (500.0 MHz, D_2O): 2.30 (dt, 1H, J_{gem} = 14.1 Hz, $J_{2'a,1'}$ = $J_{2'a,3'}$ = 6.4 Hz, H-2´a); 2.50 (ddd, 1H, J_{gem} = 14.1 Hz, $J_{2'b,1'}$ = 6.5 Hz, $J_{2'b,3'}$ = 4.7 Hz, H-2´b); 3.26 (s, 9H, $(CH_3)_3N$); 3.78 (dd, 1H; J_{gem} = 12.6 Hz, $J_{5'a,4'}$ = 4.9 Hz, H-5´a); 3.88 (dd, 1H; J_{gem} = 12.6 Hz, $J_{5'b,4'}$ = 3.3 Hz, H-5´b); 4.08 (td, 1H, (dd, 1H; $J_{4',5'a}$ = $J_{4',3'}$ = 4.7 Hz, $J_{4',5'b}$ = 3.3 Hz, H-4´); 4.43 (dt, 1H, $J_{3',2'}$ = 6.7 Hz, $J_{3',2'}$ = $J_{3',4'}$ = 4.5 Hz, H-3´); 4.51 (s, 2H, C≡CH_2); 6.20 (t, 1H, $J_{1',2'a}$ = $J_{1',2'b}$ = 6.2 Hz, H-1´); 8.37 (s, 1H, H-6).

^{13}C NMR (125.7 MHz, D_2O): 41.79 (CH_2-2´); 54.71 ($(CH_3)_3N$); 59.11 (C≡C**C**H_2); 62.85 (CH_2-5´); 71.85 (CH-3´); 84.51 and 84.97 (**C**≡**C**CH_2); 88.78 and 88.87 (CH-1´,4´); 92.02 (C-5); 149.05 (CH-6); 157.83 (C-2); 166.92 (C-4).

HR MS (ESI+) for $C_{15}H_{23}O_4N_4$ $[M + H]^+$ calcd.: 323.17138 found: 323.17155.

5-(3-trimethylaminoprop-1-yn-1-yl)-2'-deoxycytidine 5'-*O*-triphosphate (**dC^{NMe3}TP**)

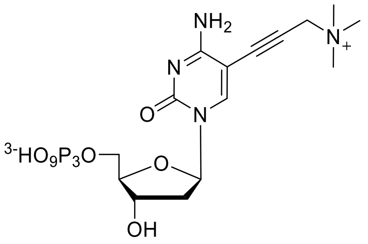

dC^{NMe3} (50.8 mg, 0.11 mmol) was dried overnight on high vacuum and then dissolved in $PO(OMe)_3$ (1 mL) in a sealed flask under argon atmosphere. The solution was cooled to 0 °C and $POCl_3$ (0.013 mL, 0.17 mmol) was added under constant stirring. The reaction was monitored by normal phase TLC using DCM/MeOH 7/1 as mobile phase. After 45 min, a precooled solution of tetrakis(tributylammonium)-pyrophosphate ($(NHBu_3)_2H_2P_2O_7$) (309.5 mg, 0.56 mmol) in MeCN (3 mL) was added followed by Bu_3N (0.1 mL, 0.45 mmol), and the resulting mixture was stirred for another 1 hour at 0 °C. Then, the reaction was quenched with an excess of 2M TEAB solution, and the solvents were evaporated. The crude product was purified by reverse-phase HPLC with a linear gradient 0 to 100% of buffer B in buffer A. The appropriate fractions were collected, evaporated, and repurified by ion-exchange HPLC (0 to 100% of 800 mM TEAB in H_2O, for details see section 7.5). The pure product was isolated as an orange-colored solid (5 mg, 0.007 mmol; 6% yield).

^{1}H NMR (500.0 MHz, D_2O): 2.33 (dt, 1H, J_{gem} = 14.1 Hz, $J_{2´a,1´}$ = $J_{2´a,3´}$ = 6.4 Hz, H-2´a); 2.48 (ddd, 1H, J_{gem} = 14.1 Hz, $J_{2´a,1´}$ = 6.3 Hz, $J_{2´a,3´}$ = 4.0 Hz, H-2´b); 3.27 (s, 9H, $(CH_3)_3N$); 4.20 - 4.30 (m, 3H; H-4´,5´); 4.52 (s, 2H, C≡CCH_2); 4.64 (m, 1H, H-3´); 6.28 (t, 1H, $J_{1´,2´a}$ = $J_{1´,2´b}$ = 6.4 Hz, H-1´); 8.44 (s, 1H, H-6).

^{13}C NMR (125.7 MHz, D_2O): 41.95 (CH_2-2´); 54.90 ($(CH_3)_3N$); 59.16 (C≡C**C**H_2); 67.07 (d, $J_{C,P}$ = 5.5 Hz, CH_2-5´); 72.33 (CH-3´); 84.42 (**C**≡CCH_2); 85.23 (C≡**C**CH_2); 87.96 (d, $J_{C,P}$ = 9.2 Hz, CH-4´); 88.68 (CH-1´); 92.4 (C-5); 149.10 (CH-6); 157.97 (C-2); 166.94 (C-4).

^{31}P NMR (202.4 MHz, D_2O): -22.43 (t, 1P, $J_{\beta,\alpha}$ = $J_{\beta,\gamma}$ = 20.1 Hz, P_β); -11.02 (d, 1P, $J_{\alpha,\beta}$ = 20.1 Hz, P_α); -9.09 (bs, 1P, P_γ).

HR MS (ESI–) for $C_{15}H_{24}O_{13}N_4P_3$ $[M – 2H]^{2–}$ calcd.: 561.05472 found: 561.05527.

7-(3-*O*-phosphate-prop-1-yn-1-yl)-2’-deoxy-7-deazaadenosine 5’-*O*-triphosphate (**dA^{OP}TP**), 7-(4-phosphoryl-but-1-yn-1-yl)-2’-deoxy-7-deazaguanosine 5’-*O*-triphosphate (**dG^{PA}TP**), 5-(3-sulfo-prop-1-yn-1-yl)-2’-deoxyuridine 5’-*O*-triphosphate (**dU^{SA}TP**), 5-(5-carboxy-pent-1-yn-1-yl)-2’-deoxycytidine 5’-*O*-triphosphate (**dC^{CA}TP**), 5-(2-phenyl-1-ethyn-1-yl)-2’-deoxyuridine 5’-*O*-triphosphate (**dU^{EPh}TP**), and 5-(pent-1-yn-1-yl)-2’-deoxycytidine 5’-*O*-triphosphate (**dC^{EAlk}TP**) were synthesized according to described protocols[5,6].

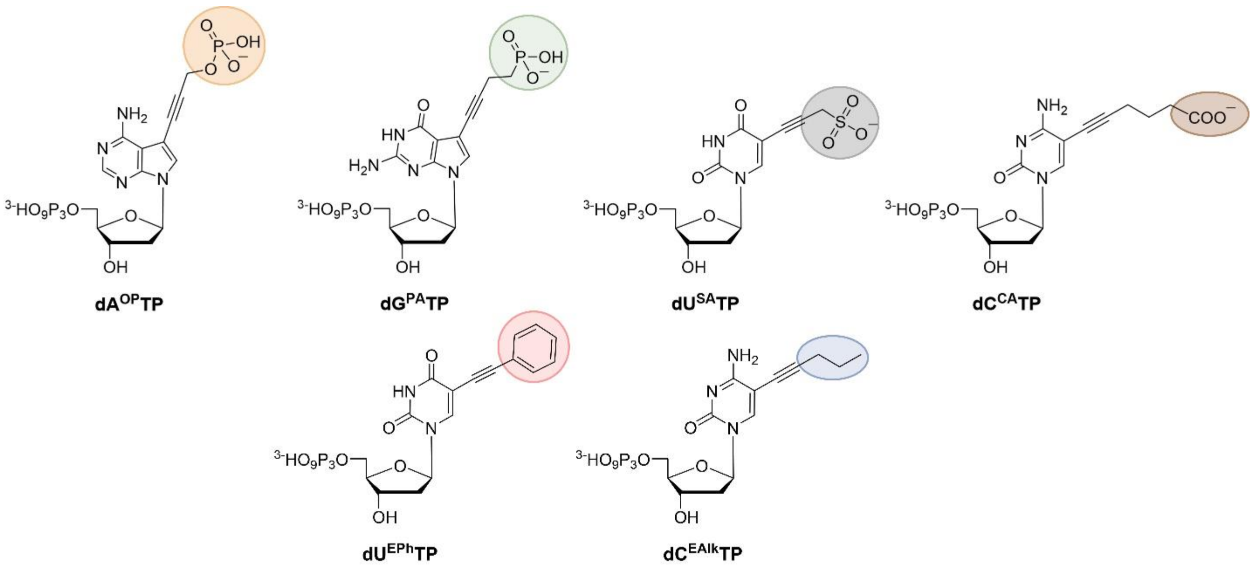

2. Experimental section – biochemistry

Synthetic oligonucleotides (ONs) were purchased from Eurofins Genomics (Germany), Generi Biotech (Czech Republic), Biomers (Germany), and GenScript Biotech (Netherlands) (for sequences see Table S1). Natural nucleoside triphosphates (dATP, dGTP, dTTP, dCTP) were purchased from ThermoScientific. KOD XL and Pwo DNA polymerases and corresponding polymerase reaction buffers were supplied by Merck Life Science; Vent(exo-) DNA polymerase and a corresponding polymerase reaction buffer were purchased from New England Biolabs. DNase I and a corresponding polymerase reaction buffer were purchased from ThermoFisher Scientific. The human pooled plasma was purchased from Biowest. DNA recovery in experiments with DNase I was calculated using ImageJ analyzer. Milli-Q water was used for all experiments. Other chemicals were of analytical grade. Streptavidin magnetic particles (Roche) were purchased from Merck Life Science. ONs' purification was performed using QIAquick PCR Purification Kit (Qiagen), Agencourt AMPure XP magnetic particles (Beckman Coulter Life science - GE Healthcare), and mini Quick Spin Oligo Columns (Roche). Sanger sequencing was done by SeqMe (Czech Republic). Used kits for gel extraction Pur-A-Lyze Maxi Dialysis Kit (Sigma Aldrich) and Amicon Ultra-0.5 Centrifugal Filters were purchased from Merck Life Science. Enzymatic reactions and annealing reactions were performed in a thermal cycler Biometra Trio (Analytik Jena). To analyze samples under denaturing conditions (50 °C, 1X TBE, pH 8.5), either 12.5% or 20% denaturing PAGE (acrylamide/bisacrylamide 19:1, 25% urea) was used. PAGE stop solution contained: 95% [v/v] formamide, 0.5 mM EDTA, 0.025% [w/v] bromophenol blue and 0.025% [w/v] xylene cyanol FF, 0.025% [w/v] SDS in Milli-Q water. To analyze samples under native conditions (0.5X TBE buffer, 120 V, 75 min), 2% agarose gel (Serva) was used; 6X DNA Gel Loading Dye (60 mM EDTA, 10 mM Tris-HCl (pH 7.6), 60% glycerol, 0.03% bromophenol blue, 0.03% xylene cyanol FF, ThermoFisher Scientific). All gels were analyzed by fluorescence imaging using Amersham Typhoon (Cytiva). Samples were concentrated on CentriVap Vacuum Concentrator system (Labconco). The analysis of ONs was done by UHPLC-MS-ESI (Agilent 1290 Infinity II Bio LC System with DAD detector and mass spectrometer MSD XT). The analysis was carried out according to standard procedures using a system of mobile phases A [12.2 mM Et_3N (triethylamine), 300 mM HFIP (1,1,1,3,3,3-hexafluoro-2-propanol) in H_2O] and B (12.2 mM Et_3N, 300 mM HFIP in MeOH) using bioZen Oligo column 1.7 µm 2.1×150 mm (Phenomenex). Deconvoluted MS spectra were obtained using UniDec[7]. The MALDI-TOF spectra were measured

on a UltrafleXtreme MALDI-TOF/TOF (Bruker) mass spectrometer with a 1 kHz smart beam II laser. The matrix consisted of 3-hydroxypicolinic acid (HPA)/picolinic acid (PA)/ammonium tartrate in ratio 9:1:1.

Table S1. List of sequences of primers and templates.

Name	Length (nt)	Sequence (5’→ 3’) [a]
Temp$^{\text{Oligo1A}}$	19	CCCTCCCATGCCGCCCATG
Temp$^{\text{Oligo1T}}$	19	CCCACCCATGCCGCCCATG
Temp$^{\text{Oligo1C}}$	19	CCCGCCCATGCCGCCCATG
Temp$^{\text{Oligo1G}}$	19	AAACCCCATGCCGCCCATG
Temp$^{\text{Prb4basII}}$	31	CTAGCATGAGCTCAGTCCCATGCCGCCCATG
Temp$^{\text{Prb4basII}}$-bio [b]	31	CTAGCATGAGCTCAGTCCCATGCCGCCCATG
Temp$^{\text{MO43}}$	43	CATGAGCTCAGTCTAGCATGAGCTCAGTCCC<br>ATGCCGCCCATG
Temp$^{\text{MO61}}$	61	GACATCATGAGAGACATCGCCTAGCATGAG<br>CTAGCATGAGCTCAGTCCCATGCCGCCCATG
Temp$^{\text{FVL-A}}$	98	GACATCATGAGAGACATCGCCTCTGGGCTA<br>ATAGGACTACTTCTAATCTGTAAGAGCAGAT<br>CCCTGGACAGGCAAGGAATACAGGTATTTT<br>GTCCTTG
Temp$^{\text{NK98}}$	98	GCAATCAGTACGAACTGACTGTCTGACTGTC<br>TAGCATGACTGTACTGTCTGTACGAACGATC<br>AGTCTGTTCAGACAGAACTAGCATACGTTCG<br>TATCG
Temp $^{\text{NK98}}$-sC3 [c]	98	GCAATCAGTACGAACTGACTGTCTGACTGTC<br>TAGCATGACTGTACTGTCTGTACGAACGATC<br>AGTCTGTTCAGACAGAACTAGCATACGTTCG<br>TATCG
Temp$^{\text{NK98_comp}}$	98	CGATACGAACGTATGCTAGTTCTGTCTGAAC<br>AGACTGATCGTTCGTACAGACAGTACAGTC

		ATGCTAGACAGTCAGACAGTCAGTTCGTACTGATTGC
Temp$^{\text{NK98}}$-bio [b]	98	GCAATCAGTACGAACTGACTGTCTGACTGTCTAGCATGACTGTACTGTCTGTACGAACGATCAGTCTGTTCAGACAGAACTAGCATACGTTCGTATCG
Temp$^{\text{NK98_comp}}$-bio [b]	98	CGATACGAACGTATGCTAGTTCTGTCTGAACAGACTGATCGTTCGTACAGACAGTACAGTCATGCTAGACAGTCAGACAGTCAGTTCGTACTGATTGC
Prim$^{\text{248short}}$-FAM [d]	15	CATGGGCGGCATGGG
Prim$^{\text{LT25TH}}$-FAM [d]	25	CAAGGACAAAATACCTGTATTCCTT
Prim$^{\text{NK98pr1}}$	25	CGATACGAACGTATGCTAGTTCTGT
Prim$^{\text{NK98pr1}}$-FAM [d]	25	CGATACGAACGTATGCTAGTTCTGT
Prim$^{\text{NK98pr2}}$	25	GCAATCAGTACGAACTGACTGTCTG
Prim$^{\text{NK98pr2}}$-Cy5 [e]	25	GCAATCAGTACGAACTGACTGTCTG
Prim$^{\text{Flank-NK98pr1}}$-FAM [d]	45	CATTCGGCTGCTCTTGATTTCGATACGAACGTATGCTAGTTCTGT
Prim^{Flank}	20	CATTCGGCTGCTCTTGATTT
Prim^{Flank}-FAM [d]	20	CATTCGGCTGCTCTTGATTT

[a] in the template ONs the segments forming duplex with the primer are underlined; [b] 5’-dual-biotinylated; [c] 3’-sC3 (three carbon spacer); [d] 5’-(6-FAM)-labelled, [e] 5’-Cy5-labelled

Table S2. List of synthesized ssONs/dsDNAs.

Name	Sequence (5'→ 3') [a]
**19ON_A^{NH2}** [b]	CATGGGCGGCATGGGA^{NH2}GGG
**19ON_U^{NMe}** [b]	CATGGGCGGCATGGGU^{NMe}GGG
**19ON_G^{NMe2}** [b]	CATGGGCGGCATGGGG^{NMe2}TTT
**19ON_C^{NMe3}** [b]	CATGGGCGGCATGGGC^{NMe3}GGG
**19DNA_A^{NH2}** [b]	CATGGGCGGCATGGGA^{NH2}GGG
**19DNA_U^{NMe}** [b]	CATGGGCGGCATGGGU^{NMe}GGG
**19DNA_G^{NMe2}** [b]	CATGGGCGGCATGGGG^{NMe2}TTT
**19DNA_C^{NMe3}** [b]	CATGGGCGGCATGGGC^{NMe3}GGG
**31ON** [b]	CATGGGCGGCATGGGACTGAGCTCATGCTAG
**31ON_A^{NH2}** [b]	CATGGGCGGCATGGGA^{NH2}CTGA^{NH2}GCTCA^{NH2}TGCTA^{NH2}G
**31ON_U^{NMe}** [b]	CATGGGCGGCATGGGACU^{NMe}GAGCU^{NMe}CAU^{NMe}GCU^{NMe}AG
**31ON_G^{NMe2}** [b]	CATGGGCGGCATGGGACTG^{NMe2}AG^{NMe2}CTCATG^{NMe2}CTAG^{NMe2}
**31ON_C^{NMe3}** [b]	CATGGGCGGCATGGGAC^{NMe3}TGAGC^{NMe3}TC^{NMe3}ATGC^{NMe3}TAG
**31DNA_A^{NH2}** [b]	CATGGGCGGCATGGGA^{NH2}CTGA^{NH2}GCTCA^{NH2}TGCTA^{NH2}G
**31DNA_U^{NMe}** [b]	CATGGGCGGCATGGGACU^{NMe}GAGCU^{NMe}CAU^{NMe}GCU^{NMe}AG
**31DNA_G^{NMe2}** [b]	CATGGGCGGCATGGGACTG^{NMe2}AG^{NMe2}CTCATG^{NMe2}CTAG^{NMe2}
**31DNA_C^{NMe3}** [b]	CATGGGCGGCATGGGAC^{NMe3}TGAGC^{NMe3}TC^{NMe3}ATGC^{NMe3}TAG
**31DNA_C^{NMe3}A^{NH2}** [b]	CATGGGCGGCATGGGA^{NH2}C^{NMe3}TGA^{NH2}GC^{NMe3}TC^{NMe3}A^{NH2}TGC^{NMe3}TA^{NH2}G
**31DNA_C^{NMe3}G^{NMe2}** [b]	CATGGGCGGCATGGGAC^{NMe3}TG^{NMe2}AG^{NMe2}C^{NMe3}TC^{NMe3}ATG^{NMe2}C^{NMe3}TAG^{NMe2}
**31DNA_C^{NMe3}U^{NMe}** [b]	CATGGGCGGCATGGGAC^{NMe3}U^{NMe}GAGC^{NMe3}U^{NMe}C^{NMe3}AU^{NMe}GC^{NMe3}U^{NMe}AG
**31DNA_A^{NH2}G^{NMe2}** [b]	CATGGGCGGCATGGGA^{NH2}CTG^{NMe2}A^{NH2}G^{NMe2}CTCA^{NH2}TG^{NMe2}CTA^{NH2}G^{NMe2}
**31DNA_A^{NH2}U^{NMe}** [b]	CATGGGCGGCATGGGA^{NH2}CU^{NMe}GA^{NH2}GCU^{NMe}CA^{NH2}U^{NMe}GCU^{NMe}A^{NH2}G
**31DNA_G^{NMe2}U^{NMe}** [b]	CATGGGCGGCATGGGACU^{NMe}G^{NMe2}AG^{NMe2}CU^{NMe}CAU^{NMe}G^{NMe2}CU^{NMe}AG^{NMe2}

**31DNA_C^{NMe3}A^{NH2}G^{NMe2}** [b]	CATGGGCGGCATGGGA^{NH2}C^{NMe3}TG^{NMe2}A^{NH2}G^{NMe2}C^{NMe3}TC^{NMe3}A^{NH2}TG^{NMe2}C^{NMe3}TA^{NH2}G^{NMe2}
**31DNA_C^{NMe3}A^{NH2}U^{NMe}** [b]	CATGGGCGGCATGGGA^{NH2}C^{NMe3}U^{NMe}GA^{NH2}GC^{NMe3}U^{NMe}C^{NMe3}A^{NH2}U^{NMe}GC^{NMe3}U^{NMe}A^{NH2}G
**31DNA_C^{NMe3}G^{NMe2}U^{NMe}** [b]	CATGGGCGGCATGGGAC^{NMe3}U^{NMe}G^{NMe2}AG^{NMe2}C^{NMe3}U^{NMe}C^{NMe3}AU^{NMe}G^{NMe2}C^{NMe3}U^{NMe}AG^{NMe2}
**31DNA_A^{NH2}G^{NMe2}U^{NMe}** [b]	CATGGGCGGCATGGGA^{NH2}CU^{NMe}G^{NMe2}A^{NH2}G^{NMe2}CU^{NMe}CA^{NH2}U^{NMe}G^{NMe2}CU^{NMe}A^{NH2}G^{NMe2}
**31ON_A^{NH2}U^{NMe}G^{NMe2}C^{NMe3}** [b]	CATGGGCGGCATGGGA^{NH2}C^{NMe3}U^{NMe}G^{NMe2}A^{NH2}G^{NMe2}C^{NMe3}U^{NMe}C^{NMe3}A^{NH2}U^{NMe}G^{NMe2}C^{NMe3}U^{NMe}A^{NH2}G^{NMe2}
**31DNA_A^{NH2}U^{NMe}G^{NMe2}C^{NMe3}** [b]	CATGGGCGGCATGGGA^{NH2}C^{NMe3}U^{NMe}G^{NMe2}A^{NH2}G^{NMe2}C^{NMe3}U^{NMe}C^{NMe3}A^{NH2}U^{NMe}G^{NMe2}C^{NMe3}U^{NMe}A^{NH2}G^{NMe2}
**31ON_A^{NH2}U^{EPh}G^{PA}C^{EAlk}** [b]	CATGGGCGGCATGGGA^{NH2}C^{EAlk}U^{EPh}G^{PA}A^{NH2}G^{PA}C^{EAlk}U^{EPh}C^{EAlk}A^{NH2}U^{EPh}G^{PA}C^{EAlk}U^{EPh}A^{NH2}G^{PA}
**31DNA_A^{NH2}U^{EPh}G^{PA}C^{EAlk}** [b]	CATGGGCGGCATGGGA^{NH2}C^{EAlk}U^{EPh}G^{PA}A^{NH2}G^{PA}C^{EAlk}U^{EPh}C^{EAlk}A^{NH2}U^{EPh}G^{PA}C^{EAlk}U^{EPh}A^{NH2}G^{PA}
**31ON_C^{CA}G^{PA}U^{SA}A^{OP}** [b]	CATGGGCGGCATGGGA^{OP}C^{CA}U^{SA}G^{PA}A^{OP}G^{PA}C^{CA}U^{SA}C^{CA}A^{OP}U^{SA}G^{PA}C^{CA}U^{SA}A^{OP}G^{PA}
**31DNA_C^{CA}G^{PA}U^{SA}A^{OP}** [b]	CATGGGCGGCATGGGA^{OP}C^{CA}U^{SA}G^{PA}A^{OP}G^{PA}C^{CA}U^{SA}C^{CA}A^{OP}U^{SA}G^{PA}C^{CA}U^{SA}A^{OP}G^{PA}
**43DNA_A^{NH2}U^{NMe}G^{NMe2}C^{NMe3}** [b]	CATGGGCGGCATGGGA^{NH2}C^{NMe3}U^{NMe}G^{NMe2}A^{NH2}G^{NMe2}C^{NMe3}U^{NMe}C^{NMe3}A^{NH2}U^{NMe}G^{NMe2}C^{NMe3}U^{NMe}A^{NH2}G^{NMe2}A^{NH2}C^{NMe3}U^{NMe}G^{NMe2}A^{NH2}G^{NMe2}C^{NMe3}U^{NMe}C^{NMe3}A^{NH2}U^{NMe}G^{NMe2}
**43DNA_A^{NH2}U^{EPh}G^{PA}C^{EAlk}** [b]	CATGGGCGGCATGGGA^{NH2}C^{EAlk}U^{EPh}G^{PA}A^{NH2}G^{PA}C^{EAlk}U^{EPh}C^{EAlk}A^{NH2}U^{EPh}G^{PA}C^{EAlk}U^{EPh}A^{NH2}G^{PA}A^{NH2}C^{EAlk}U^{EPh}G^{PA}A^{NH2}G^{PA}C^{EAlk}U^{EPh}C^{EAlk}A^{NH2}U^{EPh}G^{PA}
**61DNA_A^{NH2}U^{NMe}G^{NMe2}C^{NMe3}** [b]	CATGGGCGGCATGGGA^{NH2}C^{NMe3}U^{NMe}G^{NMe2}A^{NH2}G^{NMe2}C^{NMe3}U^{NMe}C^{NMe3}A^{NH2}U^{NMe}G^{NMe2}C^{NMe3}U^{NMe}A^{NH2}G^{NMe2}C^{NMe3}U^{NMe}C^{NMe3}A^{NH2}U^{NMe}G^{NMe2}C^{NMe3}U^{NMe}A^{NH2}G^{NMe2}G^{NMe2}C^{NMe3}G^{NMe2}A^{NH2}U^{NMe}G^{NMe2}U^{NMe}C^{NMe3}U^{NMe}C^{NMe3}U^{NMe}C^{NMe3}A^{NH2}U^{NMe}G^{NMe2}A^{NH2}U^{NMe}G^{NMe2}U^{NMe}C^{NMe3}
**61DNA_A^{NH2}U^{EPh}G^{PA}C^{EAlk}** [b]	CATGGGCGGCATGGGA^{NH2}C^{EAlk}U^{EPh}G^{PA}A^{NH2}G^{PA}C^{EAlk}U^{EPh}C^{EAlk}A^{NH2}U^{EPh}G^{PA}C^{EAlk}U^{EPh}A^{NH2}G^{PA}C^{EAlk}U^{EPh}C^{EAlk}A^{NH2}U^{EPh}G^{PA}C^{EAlk}U^{EPh}A^{NH2}G^{PA}G^{PA}C^{EAlk}G^{PA}A^{NH2}U^{EPh}G^{PA}U^{EPh}C^{EAlk}U^{EPh}C^{EAlk}U^{EPh}C^{EAlk}A^{NH2}U^{EPh}G^{PA}A^{NH2}U^{EPh}G^{PA}U^{EPh}C^{EAlk}
**98ON_A^{NH2}U^{NMe}G^{NMe2}C^{NMe3}** [b]	CGATACGAACGTATGCTAGTTCTGTC^{NMe3}U^{NMe}G^{NMe}A^{NH2}A^{NH2}C^{NMe3}A^{NH2}G^{NMe2}A^{NH2}C^{NMe3}U^{NMe}G^{NMe2}A^{NH2}U^{NMe}C^{NMe3}G^{NMe2}U^{NMe}U^{NMe}C^{NMe3}G^{NMe2}U^{NMe}A^{NH2}C^{NMe3}A^{NH2}G^{NMe2}A^{NH2}C^{NMe3}A^{NH2}G^{NMe2}U^{NMe}A^{NH2}C^{NMe3}A^{NH2}G^{NMe2}U^{NMe}C^{NMe3}A^{NH2}U^{NMe}G^{NMe2}C^{NMe3}U^{NMe}A^{NH2}G^{NMe2}A^{NH2}C^{NMe3}A^{NH2}G^{NMe2}U^{NMe}C^{NMe3}A^{NH2}G^{NMe2}A^{NH2}C^{NMe3}A^{NH2}G^{NMe2}U^{NMe}C^{NMe3}A^{NH2}G^{NMe2}U^{NMe}U^{NMe}C^{NMe3}G^{NMe2}U^{NMe}A^{NH2}C^{NMe3}U^{NMe}G^{NMe2}A^{NH2}U^{NMe}U^{NMe}G^{NMe2}C^{NMe3}
**98DNA_A^{NH2}U^{NMe}G^{NMe2}C^{NMe3}**	CGATACGAACGTATGCTAGTTCTGTC^{NMe3}U^{NMe}G^{NMe}A^{NH2}A^{NH2}C^{NMe3}A^{NH2}G^{NMe2}A^{NH2}C^{NMe3}U^{NMe}G^{NMe2}A^{NH2}U^{NMe}C^{NMe3}G^{NMe2}U^{NMe}U^{NMe}C^{NMe3}G^{NMe2}U^{NMe}A^{NH2}C^{NMe3}A^{NH2}G^{NMe2}A

	$^{NH2}C^{NMe3}A^{NH2}G^{NMe2}U^{NMe}A^{NH2}C^{NMe3}A^{NH2}G^{NMe2}U^{NMe}C^{NMe3}A^{NH2}U^{NMe}G^{NMe2}C^{NMe3}U^{NMe}A^{NH2}G^{NMe2}A^{NH2}C^{NMe3}A^{NH2}G^{NMe2}U^{NMe}C^{NMe3}A^{NH2}G^{NMe2}A^{NH2}C^{NMe3}A^{NH2}G^{NMe2}U^{NMe}C^{NMe3}A^{NH2}G^{NMe2}U^{NMe}U^{NMe}C^{NMe3}G^{NMe2}U^{NMe}A^{NH2}C^{NMe3}U^{NMe}G^{NMe2}A^{NH2}U^{NMe}U^{NMe}G^{NMe2}C^{NMe3}$
**98DNA_**$A^{NH2}U^{NMe}G^{NMe2}C^{NMe3}$ [b]	$\underline{CGATACGAACGTATGCTAGTTCTGT}C^{NMe3}U^{NMe}G^{NMe}A^{NH2}A^{NH2}C^{NMe3}A^{NH2}G^{NMe2}A^{NH2}C^{NMe3}U^{NMe}G^{NMe2}A^{NH2}U^{NMe}C^{NMe3}G^{NMe2}U^{NMe}U^{NMe}C^{NMe3}G^{NMe2}U^{NMe}A^{NH2}C^{NMe3}A^{NH2}G^{NMe2}A^{NH2}C^{NMe3}A^{NH2}G^{NMe2}U^{NMe}A^{NH2}C^{NMe3}A^{NH2}G^{NMe2}U^{NMe}C^{NMe3}A^{NH2}U^{NMe}G^{NMe2}C^{NMe3}U^{NMe}A^{NH2}G^{NMe2}A^{NH2}C^{NMe3}A^{NH2}G^{NMe2}U^{NMe}C^{NMe3}A^{NH2}G^{NMe2}A^{NH2}C^{NMe3}A^{NH2}G^{NMe2}U^{NMe}C^{NMe3}A^{NH2}G^{NMe2}U^{NMe}U^{NMe}C^{NMe3}G^{NMe2}U^{NMe}A^{NH2}C^{NMe3}U^{NMe}G^{NMe2}A^{NH2}U^{NMe}U^{NMe}G^{NMe2}C^{NMe3}$
**98cON_**$A^{NH2}U^{NMe}G^{NMe2}C^{NMe3}$ [c]	$\underline{GCAATCAGTACGAACTGACTGTCTG}A^{NH2}C^{NMe3}U^{NMe}G^{NMe2}U^{NMe}C^{NMe3}U^{NMe}A^{NH2}G^{NMe2}C^{NMe3}A^{NH2}U^{NMe}G^{NMe2}A^{NH2}C^{NMe3}U^{NMe}G^{NMe2}U^{NMe}A^{NH2}C^{NMe3}U^{NMe}G^{NMe2}U^{NMe}C^{NMe3}U^{NMe}G^{NMe2}U^{NMe}A^{NH2}C^{NMe3}G^{NMe2}A^{NH2}A^{NH2}C^{NMe3}G^{NMe2}A^{NH2}U^{NMe}C^{NMe3}A^{NH2}G^{NMe2}U^{NMe}C^{NMe3}U^{NMe}G^{NMe2}U^{NMe}U^{NMe}C^{NMe3}A^{NH2}G^{NMe2}A^{NH2}C^{NMe3}A^{NH2}G^{NMe2}A^{NH2}A^{NH2}C^{NMe3}U^{NMe}A^{NH2}G^{NMe2}C^{NMe3}A^{NH2}U^{NMe}A^{NH2}C^{NMe3}G^{NMe2}U^{NMe}U^{NMe}C^{NMe3}G^{NMe2}U^{NMe}A^{NH2}U^{NMe}C^{NMe3}G^{NMe2}$
**98cDNA_**$A^{NH2}U^{NMe}G^{NMe2}C^{NMe3}$ [c]	$\underline{GCAATCAGTACGAACTGACTGTCTG}A^{NH2}C^{NMe3}U^{NMe}G^{NMe2}U^{NMe}C^{NMe3}U^{NMe}A^{NH2}G^{NMe2}C^{NMe3}A^{NH2}U^{NMe}G^{NMe2}A^{NH2}C^{NMe3}U^{NMe}G^{NMe2}U^{NMe}A^{NH2}C^{NMe3}U^{NMe}G^{NMe2}U^{NMe}C^{NMe3}U^{NMe}G^{NMe2}U^{NMe}A^{NH2}C^{NMe3}G^{NMe2}A^{NH2}A^{NH2}C^{NMe3}G^{NMe2}A^{NH2}U^{NMe}C^{NMe3}A^{NH2}G^{NMe2}U^{NMe}C^{NMe3}U^{NMe}G^{NMe2}U^{NMe}U^{NMe}C^{NMe3}A^{NH2}G^{NMe2}A^{NH2}C^{NMe3}A^{NH2}G^{NMe2}A^{NH2}A^{NH2}C^{NMe3}U^{NMe}A^{NH2}G^{NMe2}C^{NMe3}A^{NH2}U^{NMe}A^{NH2}C^{NMe3}G^{NMe2}U^{NMe}U^{NMe}C^{NMe3}G^{NMe2}U^{NMe}A^{NH2}U^{NMe}C^{NMe3}G^{NMe2}$
**98ON_**$A^{NH2}U^{EPh}G^{PA}C^{EAlk}$	$\underline{CGATACGAACGTATGCTAGTTCTGT}C^{EAlk}U^{EPh}G^{PA}A^{NH2}A^{NH2}C^{EAlk}A^{NH2}G^{PA}A^{NH2}C^{EAlk}U^{EPh}G^{PA}A^{NH2}U^{EPh}C^{EAlk}G^{PA}U^{EPh}U^{EPh}C^{EAlk}G^{PA}U^{EPh}A^{NH2}C^{EAlk}A^{NH2}G^{PA}A^{NH2}C^{EAlk}A^{NH2}G^{PA}U^{EPh}A^{NH2}C^{EAlk}A^{NH2}G^{PA}U^{EPh}C^{EAlk}A^{NH2}U^{EPh}G^{PA}C^{EAlk}U^{EPh}A^{NH2}G^{PA}A^{NH2}C^{EAlk}A^{NH2}G^{PA}U^{EPh}C^{EAlk}A^{NH2}G^{PA}A^{NH2}C^{EAlk}A^{NH2}G^{PA}U^{EPh}C^{EAlk}A^{NH2}G^{PA}U^{EPh}U^{EPh}C^{EAlk}G^{PA}U^{EPh}A^{NH2}C^{EAlk}U^{EPh}G^{PA}A^{NH2}U^{EPh}U^{EPh}G^{PA}C^{EAlk}$
**98ON_**$A^{NH2}U^{EPh}G^{PA}C^{EAlk}$ [b]	$\underline{CGATACGAACGTATGCTAGTTCTGT}C^{EAlk}U^{EPh}G^{PA}A^{NH2}A^{NH2}C^{EAlk}A^{NH2}G^{PA}A^{NH2}C^{EAlk}U^{EPh}G^{PA}A^{NH2}U^{EPh}C^{EAlk}G^{PA}U^{EPh}U^{EPh}C^{EAlk}G^{PA}U^{EPh}A^{NH2}C^{EAlk}A^{NH2}G^{PA}A^{NH2}C^{EAlk}A^{NH2}G^{PA}U^{EPh}A^{NH2}C^{EAlk}A^{NH2}G^{PA}U^{EPh}C^{EAlk}A^{NH2}U^{EPh}G^{PA}C^{EAlk}U^{EPh}A^{NH2}G^{PA}A^{NH2}C^{EAlk}A^{NH2}G^{PA}U^{EPh}C^{EAlk}A^{NH2}G^{PA}A^{NH2}C^{EAlk}A^{NH2}G^{PA}U^{EPh}C^{EAlk}A^{NH2}G^{PA}U^{EPh}U^{EPh}C^{EAlk}G^{PA}U^{EPh}A^{NH2}C^{EAlk}U^{EPh}G^{PA}A^{NH2}U^{EPh}U^{EPh}G^{PA}C^{EAlk}$
**98DNA_**$A^{NH2}U^{EPh}G^{PA}C^{EAlk}$	$\underline{CGATACGAACGTATGCTAGTTCTGT}C^{EAlk}U^{EPh}G^{PA}A^{NH2}A^{NH2}C^{EAlk}A^{NH2}G^{PA}A^{NH2}C^{EAlk}U^{EPh}G^{PA}A^{NH2}U^{EPh}C^{EAlk}G^{PA}U^{EPh}U^{EPh}C^{EAlk}G^{PA}U^{EPh}A^{NH2}C^{EAlk}A^{NH2}G^{PA}A^{NH2}C^{EAlk}A^{NH2}G^{PA}U^{EPh}$

	$A^{NH2}C^{EAlk}A^{NH2}G^{PA}U^{EPh}C^{EAlk}A^{NH2}U^{EPh}G^{PA}C^{EAlk}U^{EPh}A^{NH2}G^{PA}A^{NH2}C^{EAlk}A^{NH2}G^{PA}U^{EPh}C^{EAlk}A^{NH2}G^{PA}A^{NH2}C^{EAlk}A^{NH2}G^{PA}U^{EPh}C^{EAlk}A^{NH2}G^{PA}U^{EPh}U^{EPh}C^{EAlk}G^{PA}U^{EPh}A^{NH2}C^{EAlk}U^{EPh}G^{PA}A^{NH2}U^{EPh}U^{EPh}G^{PA}C^{EAlk}$
**98DNA_$A^{NH2}U^{EPh}G^{PA}C^{EAlk}$** [b]	$\underline{CGATACGAACGTATGCTAGTTCTGT}C^{EAlk}U^{EPh}G^{PA}A^{NH2}A^{NH2}C^{EAlk}A^{NH2}G^{PA}A^{NH2}C^{EAlk}U^{EPh}G^{PA}A^{NH2}U^{EPh}C^{EAlk}G^{PA}U^{EPh}U^{EPh}C^{EAlk}G^{PA}U^{EPh}A^{NH2}C^{EAlk}A^{NH2}G^{PA}A^{NH2}C^{EAlk}A^{NH2}G^{PA}U^{EPh}A^{NH2}C^{EAlk}A^{NH2}G^{PA}U^{EPh}C^{EAlk}A^{NH2}U^{EPh}G^{PA}C^{EAlk}U^{EPh}A^{NH2}G^{PA}A^{NH2}C^{EAlk}A^{NH2}G^{PA}U^{EPh}C^{EAlk}A^{NH2}G^{PA}A^{NH2}C^{EAlk}A^{NH2}G^{PA}U^{EPh}C^{EAlk}A^{NH2}G^{PA}U^{EPh}U^{EPh}C^{EAlk}G^{PA}U^{EPh}A^{NH2}C^{EAlk}U^{EPh}G^{PA}A^{NH2}U^{EPh}U^{EPh}G^{PA}C^{EAlk}$
**98cON_$A^{NH2}U^{EPh}G^{PA}C^{EAlk}$**	$\underline{GCAATCAGTACGAACTGACTGTCTG}A^{NH2}C^{EAlk}U^{EPh}G^{PA}U^{EPh}C^{EAlk}U^{EPh}A^{NH2}G^{PA}C^{EAlk}A^{NH2}U^{EPh}G^{PA}A^{NH2}C^{EAlk}U^{EPh}G^{PA}U^{EPh}A^{NH2}C^{EAlk}U^{EPh}G^{PA}U^{EPh}C^{EAlk}U^{EPh}G^{PA}U^{EPh}A^{NH2}C^{EAlk}G^{PA}A^{NH2}A^{NH2}C^{EAlk}G^{PA}A^{NH2}U^{EPh}C^{EAlk}A^{NH2}G^{PA}U^{EPh}C^{EAlk}U^{EPh}G^{PA}U^{EPh}U^{EPh}C^{EAlk}A^{NH2}G^{PA}A^{NH2}C^{EAlk}A^{NH2}G^{PA}A^{NH2}A^{NH2}C^{EAlk}U^{EPh}A^{NH2}G^{PA}C^{EAlk}A^{NH2}U^{EPh}A^{NH2}C^{EAlk}G^{PA}U^{EPh}U^{EPh}C^{EAlk}G^{PA}U^{EPh}A^{NH2}U^{EPh}C^{EAlk}G^{PA}$
**98cON_$A^{NH2}U^{EPh}G^{PA}C^{EAlk}$** [c]	$\underline{GCAATCAGTACGAACTGACTGTCTG}A^{NH2}C^{EAlk}U^{EPh}G^{PA}U^{EPh}C^{EAlk}U^{EPh}A^{NH2}G^{PA}C^{EAlk}A^{NH2}U^{EPh}G^{PA}A^{NH2}C^{EAlk}U^{EPh}G^{PA}U^{EPh}A^{NH2}C^{EAlk}U^{EPh}G^{PA}U^{EPh}C^{EAlk}U^{EPh}G^{PA}U^{EPh}A^{NH2}C^{EAlk}G^{PA}A^{NH2}A^{NH2}C^{EAlk}G^{PA}A^{NH2}U^{EPh}C^{EAlk}A^{NH2}G^{PA}U^{EPh}C^{EAlk}U^{EPh}G^{PA}U^{EPh}U^{EPh}C^{EAlk}A^{NH2}G^{PA}A^{NH2}C^{EAlk}A^{NH2}G^{PA}A^{NH2}A^{NH2}C^{EAlk}U^{EPh}A^{NH2}G^{PA}C^{EAlk}A^{NH2}U^{EPh}A^{NH2}C^{EAlk}G^{PA}U^{EPh}U^{EPh}C^{EAlk}G^{PA}U^{EPh}A^{NH2}U^{EPh}C^{EAlk}G^{PA}$
**98cDNA_$A^{NH2}U^{EPh}G^{PA}C^{EAlk}$**	$\underline{GCAATCAGTACGAACTGACTGTCTG}A^{NH2}C^{EAlk}U^{EPh}G^{PA}U^{EPh}C^{EAlk}U^{EPh}A^{NH2}G^{PA}C^{EAlk}A^{NH2}U^{EPh}G^{PA}A^{NH2}C^{EAlk}U^{EPh}G^{PA}U^{EPh}A^{NH2}C^{EAlk}U^{EPh}G^{PA}U^{EPh}C^{EAlk}U^{EPh}G^{PA}U^{EPh}A^{NH2}C^{EAlk}G^{PA}A^{NH2}A^{NH2}C^{EAlk}G^{PA}A^{NH2}U^{EPh}C^{EAlk}A^{NH2}G^{PA}U^{EPh}C^{EAlk}U^{EPh}G^{PA}U^{EPh}U^{EPh}C^{EAlk}A^{NH2}G^{PA}A^{NH2}C^{EAlk}A^{NH2}G^{PA}A^{NH2}A^{NH2}C^{EAlk}U^{EPh}A^{NH2}G^{PA}C^{EAlk}A^{NH2}U^{EPh}A^{NH2}C^{EAlk}G^{PA}U^{EPh}U^{EPh}C^{EAlk}G^{PA}U^{EPh}A^{NH2}U^{EPh}C^{EAlk}G^{PA}$
**98cDNA_$A^{NH2}U^{EPh}G^{PA}C^{EAlk}$** [c]	$\underline{GCAATCAGTACGAACTGACTGTCTG}A^{NH2}C^{EAlk}U^{EPh}G^{PA}U^{EPh}C^{EAlk}U^{EPh}A^{NH2}G^{PA}C^{EAlk}A^{NH2}U^{EPh}G^{PA}A^{NH2}C^{EAlk}U^{EPh}G^{PA}U^{EPh}A^{NH2}C^{EAlk}U^{EPh}G^{PA}U^{EPh}C^{EAlk}U^{EPh}G^{PA}U^{EPh}A^{NH2}C^{EAlk}G^{PA}A^{NH2}A^{NH2}C^{EAlk}G^{PA}A^{NH2}U^{EPh}C^{EAlk}A^{NH2}G^{PA}U^{EPh}C^{EAlk}U^{EPh}G^{PA}U^{EPh}U^{EPh}C^{EAlk}A^{NH2}G^{PA}A^{NH2}C^{EAlk}A^{NH2}G^{PA}A^{NH2}A^{NH2}C^{EAlk}U^{EPh}A^{NH2}G^{PA}C^{EAlk}A^{NH2}U^{EPh}A^{NH2}C^{EAlk}G^{PA}U^{EPh}U^{EPh}C^{EAlk}G^{PA}U^{EPh}A^{NH2}U^{EPh}C^{EAlk}G^{PA}$
**98DNA_ds$A^{NH2}U^{EPh}G^{PA}C^{EAlk}$** [d]	$\underline{CGATACGAACGTATGCTAGTTCTGT}C^{EAlk}U^{EPh}G^{PA}A^{NH2}A^{NH2}C^{EAlk}A^{NH2}G^{PA}A^{NH2}C^{EAlk}U^{EPh}G^{PA}A^{NH2}U^{EPh}C^{EAlk}G^{PA}U^{EPh}U^{EPh}C^{EAlk}G^{PA}U^{EPh}A^{NH2}C^{EAlk}A^{NH2}G^{PA}A^{NH2}C^{EAlk}A^{NH2}G^{PA}U^{EPh}A^{NH2}C^{EAlk}A^{NH2}G^{PA}U^{EPh}C^{EAlk}A^{NH2}U^{EPh}G^{PA}C^{EAlk}U^{EPh}A^{NH2}G^{PA}A^{NH2}C^{EAlk}A^{NH2}G^{PA}U^{EPh}C^{EAlk}A^{NH2}G^{PA}A^{NH2}C^{EAlk}A^{NH2}G^{PA}U^{EPh}C^{EAlk}A^{NH2}G^{PA}U^{EPh}U^{EPh}C^{EAlk}G^{PA}U^{EPh}A^{NH2}C^{EAlk}U^{EPh}G^{PA}A^{NH2}U^{EPh}U^{EPh}G^{PA}C^{EAlk}$

**118DNA_A^{NH2}U^{NMe}G^{NMe2}C^{NMe3}** [b]	CATTCGGCTGCTCTTGATTTCGATACGAACGTATGCTAGTTCTGTC^{NMe3}U^{NMe}G^{NMe}A^{NH2}A^{NH2}C^{NMe3}A^{NH2}G^{NMe2}A^{NH2}C^{NMe3}U^{NMe}G^{NMe2}A^{NH2}U^{NMe}C^{NMe3}G^{NMe2}U^{NMe}U^{NMe}C^{NMe3}G^{NMe2}U^{NMe}A^{NH2}C^{NMe3}A^{NH2}G^{NMe2}A^{NH2}C^{NMe3}A^{NH2}G^{NMe2}U^{NMe}A^{NH2}C^{NMe3}A^{NH2}G^{NMe2}U^{NMe}C^{NMe3}A^{NH2}U^{NMe}G^{NMe2}C^{NMe3}U^{NMe}A^{NH2}G^{NMe2}A^{NH2}C^{NMe3}A^{NH2}G^{NMe2}U^{NMe}C^{NMe3}A^{NH2}G^{NMe2}A^{NH2}C^{NMe3}A^{NH2}G^{NMe2}U^{NMe}C^{NMe3}A^{NH2}G^{NMe2}U^{NMe}U^{NMe}C^{NMe3}G^{NMe2}U^{NMe}A^{NH2}C^{NMe3}U^{NMe}G^{NMe2}A^{NH2}U^{NMe}U^{NMe}G^{NMe2}C^{NMe3}
**118DNA_A^{NH2}U^{EPh}G^{PA}C^{EAlk}** [b]	CATTCGGCTGCTCTTGATTTCGATACGAACGTATGCTAGTTCTGTC^{EAlk}U^{EPh}G^{PA}A^{NH2}A^{NH2}C^{EAlk}A^{NH2}G^{PA}A^{NH2}C^{EAlk}U^{EPh}G^{PA}A^{NH2}U^{EPh}C^{EAlk}G^{PA}U^{EPh}U^{EPh}C^{EAlk}G^{PA}U^{EPh}A^{NH2}C^{EAlk}A^{NH2}G^{PA}A^{NH2}C^{EAlk}A^{NH2}G^{PA}U^{EPh}A^{NH2}C^{EAlk}A^{NH2}G^{PA}U^{EPh}C^{EAlk}A^{NH2}U^{EPh}G^{PA}C^{EAlk}U^{EPh}A^{NH2}G^{PA}A^{NH2}C^{EAlk}A^{NH2}G^{PA}U^{EPh}C^{EAlk}A^{NH2}G^{PA}A^{NH2}C^{EAlk}A^{NH2}G^{PA}U^{EPh}C^{EAlk}A^{NH2}G^{PA}U^{EPh}U^{EPh}C^{EAlk}G^{PA}U^{EPh}A^{NH2}C^{EAlk}U^{EPh}G^{PA}A^{NH2}U^{EPh}U^{EPh}G^{PA}C^{EAlk}
**98PCR_A^{NH2}** [d]	CGATACGAACGTATGCTAGTTCTGTCTGA^{NH2}A^{NH2}CA^{NH2}GA^{NH2}CTGA^{NH2}TCGTTCGTA^{NH2}CA^{NH2}GA^{NH2}CA^{NH2}GTA^{NH2}CA^{NH2}GTCA^{NH2}TGCTA^{NH2}GA^{NH2}CA^{NH2}GTCA^{NH2}GA^{NH2}CA^{NH2}GTCA^{NH2}GTTCGTA^{NH2}CTGA^{NH2}TTGC
**98PCR_U^{NMe}** [d]	CGATACGAACGTATGCTAGTTCTGTCU^{NMe}GAACAGACU^{NMe}GAU^{NMe}CGU^{NMe}U^{NMe}CGU^{NMe}ACAGACAGU^{NMe}ACAGU^{NMe}CAU^{NMe}GCU^{NMe}AGACAGU^{NMe}CAGACAGU^{NMe}CAGU^{NMe}U^{NMe}CGU^{NMe}ACU^{NMe}GAU^{NMe}U^{NMe}GC
**98PCR_G^{NMe2}** [d]	CGATACGAACGTATGCTAGTTCTGTCTG^{NMe2}AACAG^{NMe2}ACTG^{NMe2}ATCG^{NMe2}TTCG^{NMe2}TACAG^{NMe2}ACAG^{NMe2}TACAG^{NMe2}TCATG^{NMe2}CTAG^{NMe2}ACAG^{NMe2}TCAG^{NMe2}ACAG^{NMe2}TCAG^{NMe2}TTCG^{NMe2}TACTG^{NMe2}ATTG^{NMe2}C
**98PCR_C^{NMe3}** [d]	CGATACGAACGTATGCTAGTTCTGTC^{NMe3}TGAAC^{NMe3}AGAC^{NMe3}TGATC^{NMe3}GTTC^{NMe3}GTAC^{NMe3}AGAC^{NMe3}AGTAC^{NMe3}AGTC^{NMe3}ATGC^{NMe3}TAGAC^{NMe3}AGTC^{NMe3}AGAC^{NMe3}AGTC^{NMe3}AGTTC^{NMe3}GTAC^{NMe3}TGATTGC^{NMe3}
**98PCR_C^{NMe3}A^{NH2}** [d]	CGATACGAACGTATGCTAGTTCTGTC^{NMe3}TGA^{NH2}A^{NH2}C^{NMe3}A^{NH2}GA^{NH2}C^{NMe3}TGA^{NH2}TC^{NMe3}GTTC^{NMe3}GTA^{NH2}C^{NMe3}A^{NH2}GA^{NH2}C^{NMe3}A^{NH2}GTA^{NH2}C^{NMe3}A^{NH2}GTC^{NMe3}A^{NH2}TGC^{NMe3}TA^{NH2}GA^{NH2}C^{NMe3}A^{NH2}GTC^{NMe3}A^{NH2}GA^{NH2}C^{NMe3}A^{NH2}GTC^{NMe3}A^{NH2}GTTC^{NMe3}GTA^{NH2}C^{NMe3}TGA^{NH2}TTGC^{NMe3}
**98PCR_C^{NMe3}G^{NMe2}** [d]	CGATACGAACGTATGCTAGTTCTGTC^{NMe3}TG^{NMe}AAC^{NMe3}AG^{NMe2}AC^{NMe3}TG^{NMe2}ATC^{NMe3}G^{NMe2}TTC^{NMe3}G^{NMe2}TAC^{NMe3}AG^{NMe2}AC^{NMe3}AG^{NMe2}TAC^{NMe3}AG^{NMe2}TC^{NMe3}ATG^{NMe2}C^{NMe3}TAG^{NMe2}AC^{NMe3}AG^{NMe2}TC^{NMe3}AG^{NMe2}AC^{NMe3}AG^{NMe2}TC^{NMe3}AG^{NMe2}TTC^{NMe3}G^{NMe2}TAC^{NMe3}TG^{NMe2}ATTG^{NMe2}C^{NMe3}
**98PCR_C^{NMe3}U^{NMe}** [d]	CGATACGAACGTATGCTAGTTCTGTC^{NMe3}U^{NMe}GAAC^{NMe3}AGAC^{NMe3}U^{NMe}GAU^{NMe}C^{NMe3}GU^{NMe}U^{NMe}C^{NMe3}GU^{NMe}AC^{NMe3}AGAC^{NMe3}AGU^{NMe}AC^{NMe3}AGU^{NMe}C^{NMe3}AU^{NMe}GC

	^{NMe3}U^{NMe}AGAC^{NMe3}AGU^{NMe}C^{NMe3}AGAC^{NMe3}AGU^{NMe}C^{NMe3}AGU^{NMe}U^{NMe}C^{NMe3}GU^{NMe}AC^{NMe3}U^{NMe}GAU^{NMe}U^{NMe}GC^{NMe3}
**98PCR_A^{NH2}G^{NMe2}** *d*	CGATACGAACGTATGCTAGTTCTGTCTG^{NMe}A^{NH2}A^{NH2}CA^{NH2}G^{NMe2}A^{NH2}CTG^{NMe2}A^{NH2}TCG^{NMe2}TTCG^{NMe2}TA^{NH2}CA^{NH2}G^{NMe2}A^{NH2}CA^{NH2}G^{NMe2}TA^{NH2}CA^{NH2}G^{NMe2}TCA^{NH2}TG^{NMe2}CTA^{NH2}G^{NMe2}A^{NH2}CA^{NH2}G^{NMe2}TCA^{NH2}G^{NMe2}A^{NH2}CA^{NH2}G^{NMe2}TCA^{NH2}G^{NMe2}TTCG^{NMe2}TA^{NH2}CTG^{NMe2}A^{NH2}TTG^{NMe2}C
**98PCR_A^{NH2}U^{NMe}** *d*	CGATACGAACGTATGCTAGTTCTGTCU^{NMe}GA^{NH2}A^{NH2}CA^{NH2}GA^{NH2}CU^{NMe}GA^{NH2}U^{NMe}CGU^{NMe}U^{NMe}CGU^{NMe}A^{NH2}CA^{NH2}GA^{NH2}CA^{NH2}GU^{NMe}A^{NH2}CA^{NH2}GU^{NMe}CA^{NH2}U^{NMe}GCU^{NMe}A^{NH2}GA^{NH2}CA^{NH2}GU^{NMe}CA^{NH2}GA^{NH2}CA^{NH2}GU^{NMe}CA^{NH2}GU^{NMe}U^{NMe}CGU^{NMe}A^{NH2}CU^{NMe}GA^{NH2}U^{NMe}U^{NMe}GC
**98PCR_G^{NMe2}U^{NMe}** *d*	CGATACGAACGTATGCTAGTTCTGTCU^{NMe}G^{NMe}AACAG^{NMe2}ACU^{NMe}G^{NMe2}AU^{NMe}CG^{NMe2}U^{NMe}U^{NMe}CG^{NMe2}U^{NMe}ACAG^{NMe2}ACAG^{NMe2}U^{NMe}ACAG^{NMe2}U^{NMe}CAU^{NMe}G^{NMe2}CU^{NMe}AG^{NMe2}ACAG^{NMe2}U^{NMe}CAG^{NMe2}ACAG^{NMe2}U^{NMe}CAG^{NMe2}U^{NMe}U^{NMe}CG^{NMe2}U^{NMe}ACU^{NMe}G^{NMe2}AU^{NMe}U^{NMe}G^{NMe2}C
**98PCR_A^{NH2}G^{NMe2}C^{NMe3}** *d*	CGATACGAACGTATGCTAGTTCTGTC^{NMe3}TG^{NMe}A^{NH2}A^{NH2}C^{NMe3}A^{NH2}G^{NMe2}A^{NH2}C^{NMe3}TG^{NMe2}A^{NH2}TC^{NMe3}G^{NMe2}TTC^{NMe3}G^{NMe2}TA^{NH2}C^{NMe3}A^{NH2}G^{NMe2}A^{NH2}C^{NMe3}A^{NH2}G^{NMe2}TA^{NH2}C^{NMe3}A^{NH2}G^{NMe2}TC^{NMe3}A^{NH2}TG^{NMe2}C^{NMe3}TA^{NH2}G^{NMe2}A^{NH2}C^{NMe3}A^{NH2}G^{NMe2}TC^{NMe3}A^{NH2}G^{NMe2}A^{NH2}C^{NMe3}A^{NH2}G^{NMe2}TC^{NMe3}A^{NH2}G^{NMe2}TTC^{NMe3}G^{NMe2}TA^{NH2}C^{NMe3}TG^{NMe2}A^{NH2}TTG^{NMe2}C^{NMe3}
**98ON_A^{NH2}U^{NMe}C^{NMe3}** *b*	CGATACGAACGTATGCTAGTTCTGTC^{NMe3}U^{NMe}GA^{NH2}A^{NH2}C^{NMe3}A^{NH2}GA^{NH2}C^{NMe3}U^{NMe}GA^{NH2}U^{NMe}C^{NMe3}GU^{NMe}U^{NMe}C^{NMe3}GU^{NMe}A^{NH2}C^{NMe3}A^{NH2}GA^{NH2}C^{NMe3}A^{NH2}GU^{NMe}A^{NH2}C^{NMe3}A^{NH2}GU^{NMe}C^{NMe3}A^{NH2}U^{NMe}GC^{NMe3}U^{NMe}A^{NH2}GA^{NH2}C^{NMe3}A^{NH2}GU^{NMe}C^{NMe3}A^{NH2}GA^{NH2}C^{NMe3}A^{NH2}GU^{NMe}C^{NMe3}A^{NH2}GU^{NMe}U^{NMe}C^{NMe3}GU^{NMe}A^{NH2}C^{NMe3}U^{NMe}GA^{NH2}U^{NMe}U^{NMe}GC^{NMe3}
**98PCR_U^{NMe}G^{NMe2}C^{NMe3}** *d*	CGATACGAACGTATGCTAGTTCTGTC^{NMe3}U^{NMe}G^{NMe}AAC^{NMe3}AG^{NMe2}AC^{NMe3}U^{NMe}G^{NMe2}AU^{NMe}C^{NMe3}G^{NMe2}U^{NMe}U^{NMe}C^{NMe3}G^{NMe2}U^{NMe}AC^{NMe3}AG^{NMe2}AC^{NMe3}AG^{NMe2}U^{NMe}AC^{NMe3}AG^{NMe2}U^{NMe}C^{NMe3}AU^{NMe}G^{NMe2}C^{NMe3}U^{NMe}AG^{NMe2}AC^{NMe3}AG^{NMe2}U^{NMe}C^{NMe3}AG^{NMe2}AC^{NMe3}AG^{NMe2}U^{NMe}C^{NMe3}AG^{NMe2}U^{NMe}U^{NMe}C^{NMe3}G^{NMe2}U^{NMe}AC^{NMe3}U^{NMe}G^{NMe2}AU^{NMe}U^{NMe}G^{NMe2}C^{NMe3}
**98PCR_A^{NH2}U^{NMe}G^{NMe2}** *d*	CGATACGAACGTATGCTAGTTCTGTCU^{NMe}G^{NMe}A^{NH2}A^{NH2}CA^{NH2}G^{NMe2}A^{NH2}CU^{NMe}G^{NMe2}A^{NH2}U^{NMe}CG^{NMe2}U^{NMe}U^{NMe}CG^{NMe2}U^{NMe}A^{NH2}CA^{NH2}G^{NMe2}A^{NH2}CA^{NH2}G^{NMe2}U^{NMe}A^{NH2}CA^{NH2}G^{NMe2}U^{NMe}CA^{NH2}U^{NMe}G^{NMe2}CU^{NMe}A^{NH2}G^{NMe2}A^{NH2}CA^{NH2}G^{NMe2}U^{NMe}CA^{NH2}G^{NMe2}A^{NH2}CA^{NH2}G^{NMe2}U^{NMe}CA^{NH2}G

	$^{NMe2}U^{NMe}U^{NMe}CG^{NMe2}U^{NMe}A^{NH2}CU^{NMe}G^{NMe2}A^{NH2}U^{NMe}U^{NMe}G^{NMe2}C$
**98PCR_**$\mathbf{A^{NH2}U^{EPh}G^{PA}C^{EAlk}}$ [d]	$\underline{CGATACGAACGTATGCTAGTTCTGT}C^{EAlk}U^{EPh}G^{PA}A^{NH2}A^{NH2}C^{EAlk}A^{NH2}G^{PA}A^{NH2}C^{EAlk}U^{EPh}G^{PA}A^{NH2}U^{EPh}C^{EAlk}G^{PA}U^{EPh}U^{EPh}C^{EAlk}G^{PA}U^{EPh}A^{NH2}C^{EAlk}A^{NH2}G^{PA}A^{NH2}C^{EAlk}A^{NH2}G^{PA}U^{EPh}A^{NH2}C^{EAlk}A^{NH2}G^{PA}U^{EPh}C^{EAlk}A^{NH2}U^{EPh}G^{PA}C^{EAlk}U^{EPh}A^{NH2}G^{PA}A^{NH2}C^{EAlk}A^{NH2}G^{PA}U^{EPh}C^{EAlk}A^{NH2}G^{PA}A^{NH2}C^{EAlk}A^{NH2}G^{PA}U^{EPh}C^{EAlk}A^{NH2}G^{PA}U^{EPh}U^{EPh}C^{EAlk}G^{PA}U^{EPh}A^{NH2}C^{EAlk}U^{EPh}G^{PA}A^{NH2}U^{EPh}U^{EPh}G^{PA}C^{EAlk}$
**98ON** [b]	$\underline{CGATACGAACGTATGCTAGTTCTGT}$CTGAACAGACTGATCGTTCGTACAGACAGTACAGTCATGCTAGACAGTCAGACAGTCAGTTCGTACTGATTGC
**98DNA**	$\underline{CGATACGAACGTATGCTAGTTCTGT}$CTGAACAGACTGATCGTTCGTACAGACAGTACAGTCATGCTAGACAGTCAGACAGTCAGTTCGTACTGATTGC
**98DNA** [b]	$\underline{CGATACGAACGTATGCTAGTTCTGT}$CTGAACAGACTGATCGTTCGTACAGACAGTACAGTCATGCTAGACAGTCAGACAGTCAGTTCGTACTGATTGC
**98ON_**$\mathbf{C^{CA}G^{PA}U^{SA}A^{OP}}$ [b]	$\underline{CGATACGAACGTATGCTAGTTCTGT}C^{CA}U^{SA}G^{PA}A^{OP}A^{OP}C^{CA}A^{OP}G^{PA}A^{OP}C^{CA}U^{SA}G^{PA}A^{OP}U^{SA}C^{CA}G^{PA}U^{SA}U^{SA}C^{CA}G^{PA}U^{SA}A^{OP}C^{CA}A^{OP}G^{PA}A^{OP}C^{CA}A^{OP}G^{PA}U^{SA}A^{OP}C^{CA}A^{OP}G^{PA}U^{SA}C^{CA}A^{OP}U^{SA}G^{PA}C^{CA}U^{SA}A^{OP}G^{PA}A^{OP}C^{CA}A^{OP}G^{PA}U^{SA}C^{CA}A^{OP}G^{PA}A^{OP}C^{CA}A^{OP}G^{PA}U^{SA}C^{CA}A^{OP}G^{PA}U^{SA}U^{SA}C^{CA}G^{PA}U^{SA}A^{OP}C^{CA}U^{SA}G^{PA}A^{OP}U^{SA}U^{SA}G^{PA}C^{CA}$
**98DNA_**$\mathbf{C^{CA}G^{PA}U^{SA}A^{OP}}$ [b]	$\underline{CGATACGAACGTATGCTAGTTCTGT}C^{CA}U^{SA}G^{PA}A^{OP}A^{OP}C^{CA}A^{OP}G^{PA}A^{OP}C^{CA}U^{SA}G^{PA}A^{OP}U^{SA}C^{CA}G^{PA}U^{SA}U^{SA}C^{CA}G^{PA}U^{SA}A^{OP}C^{CA}A^{OP}G^{PA}A^{OP}C^{CA}A^{OP}G^{PA}U^{SA}A^{OP}C^{CA}A^{OP}G^{PA}U^{SA}C^{CA}A^{OP}U^{SA}G^{PA}C^{CA}U^{SA}A^{OP}G^{PA}A^{OP}C^{CA}A^{OP}G^{PA}U^{SA}C^{CA}A^{OP}G^{PA}A^{OP}C^{CA}A^{OP}G^{PA}U^{SA}C^{CA}A^{OP}G^{PA}U^{SA}U^{SA}C^{CA}G^{PA}U^{SA}A^{OP}C^{CA}U^{SA}G^{PA}A^{OP}U^{SA}U^{SA}G^{PA}C^{CA}$

Note: ON – single-stranded DNA; DNA – double-stranded DNA (a complementary strand is an unmodified template); [a] primer region underlined; [b] 5'-(6-FAM)-labelled extended strand after PEX; [c] 5'-Cy5-labelled extended strand after PEX; [d] a complementary strand is modified in the same manner using modification under study (primer regions are not modified)

2.1. PEX – Single incorporation (one modified $dN^{R}TP$)

The reaction mixture (10 µL) contained one of 19-mer templates $Temp^{Oligo1A}$ / $Temp^{Oligo1T}$ / $Temp^{Oligo1C}$ / $Temp^{Oligo1G}$ (3 µM, 0.75 µL), primer $Prim^{248short}$-FAM (3 µM, 0.5 µL), dGTP (dTTP in the case of $Temp^{Oligo1G}$) (0.25 mM, 1 µL), modified $\mathbf{dN^{R}TP}$ of study (R = NH2, NMe, NMe2, NMe3; N = A, U, G, C) (2 mM, 1 µL each), either KOD XL or Vent(exo-) DNA polymerase (units specification in Table S3) and a corresponding reaction buffer (10X, 1 µL) as supplied by the

manufacturer. The reaction mixture was incubated for 30 min at 60 °C, stopped by addition of PAGE stop solution (10 µL) and denatured for 3 min at 95 °C. Samples were analyzed on 12.5% PAGE and visualized using fluorescence imaging (Figure S1).

Table S3. Reaction condition specifications for single incorporation.

Template	KOD XL	Vent(exo-)	**dN^{R}TP**
Temp^{Oligo1A}	0.01 U	0.05 U	**dA^{NH2}TP**
Temp^{Oligo1T}	0.01 U	0.05 U	**dU^{NMe}TP**
Temp^{Oligo1G}	0.1 U	0.2 U	**dG^{NMe2}TP**
Temp^{Oligo1C}	0.1 U	0.2 U	**dC^{NMe3}TP**

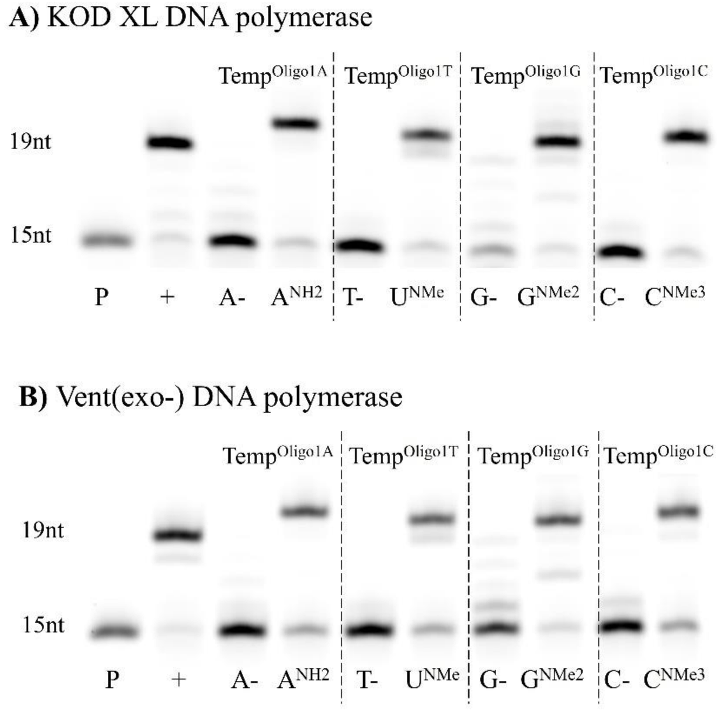

Figure S1. Denaturing PAGE analysis of PEX reaction with one modified **dN^{R}TP** using: A) KOD XL DNA polymerase, B) Vent(exo-) DNA polymerase: (P) primer Prim^{248short}-FAM; template Temp^{Oligo1A}: (+) dATP, dGTP; (A-) dGTP; (A^{NH2}) **dA^{NH2}TP**, dGTP; template Temp^{Oligo1T}: (T-) dGTP; (U^{NMe}) **dU^{NMe}TP**, dGTP; template Temp^{Oligo1G}: (G-) dTTP; (G^{NMe2}) **dG^{NMe2}TP**, dTTP; template Temp^{Oligo1C}: (C-) dGTP; (C^{NMe3}) **dC^{NMe3}TP**, dGTP.

2.2. PEX – Multiple incorporation (one modified dN^{R}TP)

The reaction mixture (10 µL) contained 31-mer template Temp^{Prb4basII} (3 µM, 0.75 µL), primer Prim^{248short}-FAM (3 µM, 0.5 µL), appropriate natural dNTPs (0.25 mM, 1 µL each), modified **dN^{R}TP** of study (R = NH2, NMe, NMe2, NMe3; N = A, U, G, C) (2 mM, 1 µL each), either KOD XL or Vent(exo-) DNA polymerase (units specification in Table S4) and a corresponding reaction buffer (10X, 1 µL) as supplied by the manufacturer. The positive control contained either 0.3 U of KOD XL or 0.5 U of Vent(exo-) DNA polymerase and natural dNTPs (1 mM, 1 µL). The reaction mixture was incubated for 30 min at 60 °C, stopped by addition of PAGE stop solution (10 µL), and denatured for 3 min at 95 °C. Samples were analyzed on 12.5% PAGE and visualized using fluorescence imaging (Figure S2).

Table S4. Reaction condition specifications for multiple incorporation.

dN^{R}TP	KOD XL	Vent(exo-)
**dA^{NH2}TP**	0.01 U	0.05 U
**dU^{NMe}TP**	0.1 U	0.2 U
**dG^{NMe2}TP**	0.3 U	0.5 U
**dC^{NMe3}TP**	0.3 U	0.5 U

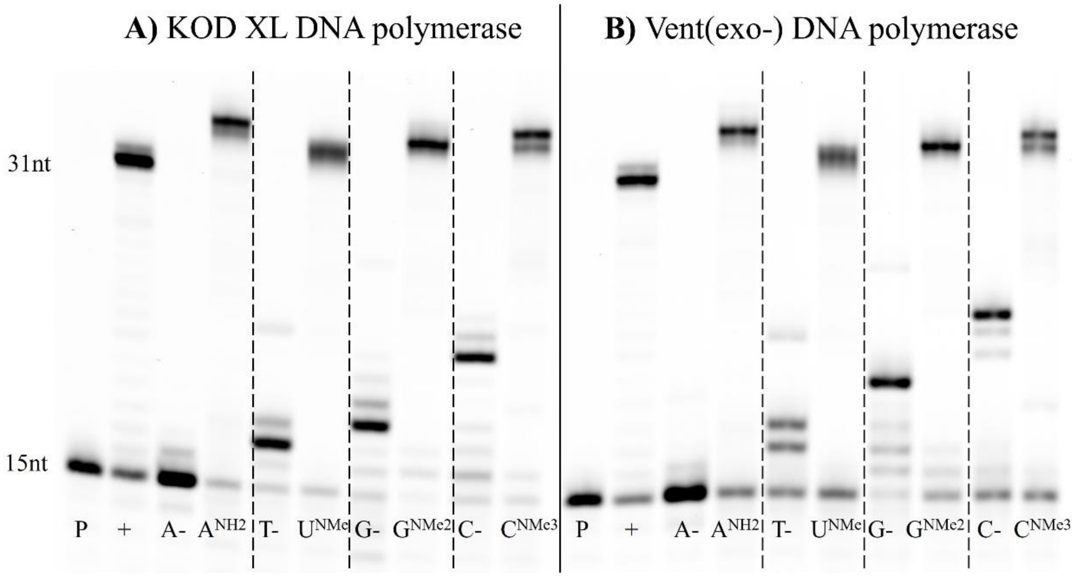

Figure S2. Denaturing PAGE analysis of PEX reaction with one modified **dN^{R}TP** using: A) KOD XL DNA polymerase, B) Vent(exo-) DNA polymerase: (P) primer Prim^{248short}-FAM;

template Temp^{Prb4basII}: (+) natural dNTPs; (A-) dCTP, dGTP, dTTP; (A^{NH2}) dCTP, **dA^{NH2}TP**, dGTP, dTTP; (T-) dCTP, dATP, dGTP; (U^{NMe}) dCTP, dATP, dGTP, **dU^{NMe}TP**; (G-) dCTP, dATP, dTTP; (G^{NMe2}) dCTP, dATP, **dG^{NMe2}TP**, dTTP; (C-) dATP, dGTP, dTTP; (C^{NMe3}) **dC^{NMe3}TP**, dATP, dGTP, dTTP.

2.3. PEX – Multiple incorporation (two, three, and four modified dN^{R}TPs)

The reaction mixture (10 µL) contained 31-mer template Temp^{Prb4basII} (3 µM, 0.75 µL), primer Prim^{248short}-FAM (3 µM, 0.5 µL), appropriate natural dNTPs (0.25 mM, 1 µL) or none in the case of four modifications, modified **dN^{R}TP**s of study (R = NH2, NMe, NMe2, NMe3; N = A, U, G, C) (1 µL, see specifications in Table S5), KOD XL DNA polymerase (Table S5), and the enzyme reaction buffer (10X, 1 µL) as supplied by the manufacturer. The reaction mixture was incubated for 30 min at 72 °C, stopped by addition of PAGE stop solution (10 µL), and denatured for 5 min at 95 °C. Samples were analyzed on 20% PAGE and visualized using fluorescence imaging (Figure S3).

Table S5. Reaction condition specifications for multiple incorporation of combinations of **dN^{R}TP**s.

Lanes in Figure S3	Natural dNTPs	KOD XL	**dA^{NH2}TP**	**dU^{NMe}TP**	**dG^{NMe2}TP**	**dC^{NMe3}TP**
5	dCTP, dGTP	0.01 U	0.25 mM	2 mM	–	–
8	dCTP, dTTP	0.1 U	0.25 mM	–	2 mM	–
11	dGTP, dTTP	0.2 U	0.25 mM	–	–	2 mM
14	dCTP, dATP	0.1 U	–	2 mM	2 mM	–
17	dATP, dGTP	0.7 U	–	2 mM	–	2 mM
20	dATP, dTTP	0.2 U	–	–	2 mM	2 mM
24	dCTP	0.1 U	0.25 mM	2 mM	2 mM	–
28	dGTP	0.5 U	0.25 mM	2 mM	–	2 mM
32	dTTP	0.6 U	0.25 mM	–	2 mM	2 mM
36	dATP	0.5 U	–	2 mM	2 mM	2 mM
41	–	0.6 U	0.25 mM	2 mM	2 mM	2 mM

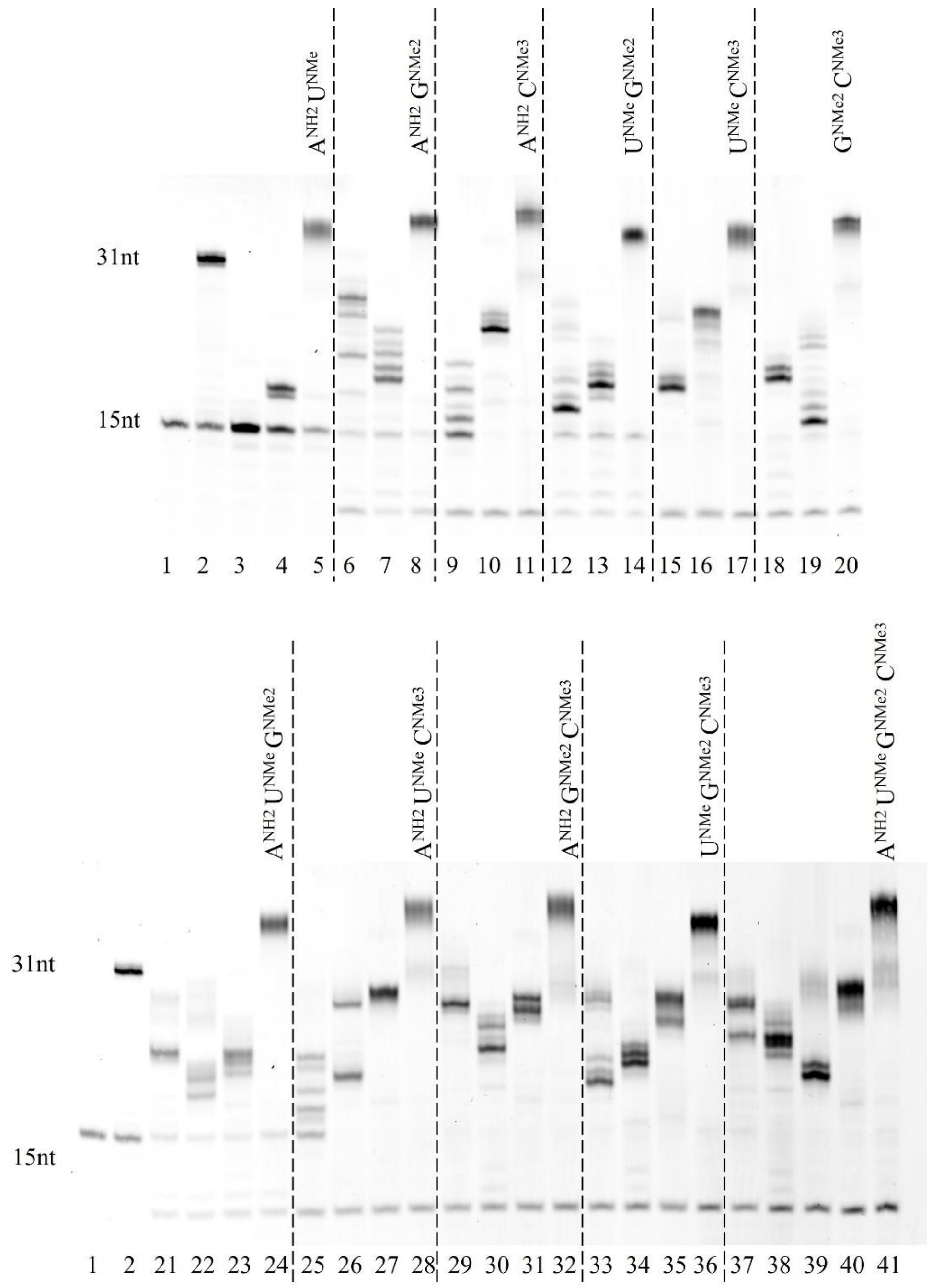

Figure S3. Denaturing PAGE analysis of PEX reaction with combinations of modified **dN^{R}TP**s using KOD XL DNA polymerase, primer Prim^{248short}-FAM, and template Temp^{Prb4basII}: (lanes 1) primer Prim^{248short}-FAM; (lanes 2) natural dNTPs; (lane 3) dCTP, dGTP, **dU^{NMe}TP**; (lane 4) dCTP, **dA^{NH2}TP**, dGTP; (lane 5) dCTP, **dA^{NH2}TP**, dGTP, **dU^{NMe}TP**; (lane 6) dCTP, **dG^{NMe2}TP**, dTTP; (lane 7) dCTP, **dA^{NH2}TP**, dTTP; (lane 8) dCTP, **dA^{NH2}TP**, **dG^{NMe2}TP**, dTTP; (lane 9) **dC^{NMe3}TP**, dGTP, dTTP; (lane 10) **dA^{NH2}TP**, dGTP, dTTP; (lane 11) **dC^{NMe3}TP**,

dA^{NH2}TP, dGTP, dTTP; (lane 12) dCTP, dATP, **dG^{NMe2}TP**; (lane 13) dCTP, dATP, **dU^{NMe}TP**; (lane 14) dCTP, dATP, **dG^{NMe2}TP**, **dU^{NMe}TP**; (lane 15) **dC^{NMe3}TP**, dATP, dGTP; (lane 16) dATP, dGTP, **dU^{NMe}TP**; (lane 17) **dC^{NMe3}TP**, dATP, dGTP, **dU^{NMe}TP**; (lane 18) **dC^{NMe3}TP**, dATP, dTTP; (lane 19) dATP, **dG^{NMe2}TP**, dTTP; (lane 20) **dC^{NMe3}TP**, dATP, **dG^{NMe2}TP**, dTTP; (lane 21) dCTP, **dG^{NMe2}TP**, **dU^{NMe}TP**; (lane 22) dCTP, **dA^{NH2}TP**, **dG^{NMe2}TP**; (lane 23) dCTP, **dA^{NH2}TP**, **dU^{NMe}TP**; (lane 24) dCTP, **dA^{NH2}TP**, **dG^{NMe2}TP**, **dU^{NMe}TP**; (lane 25) **dC^{NMe3}TP**, dGTP, **dU^{NMe}TP**; (lane 26) **dC^{NMe3}TP**, **dA^{NH2}TP**, dGTP; (lane 27) **dA^{NH2}TP**, dGTP, **dU^{NMe}TP**; (lane 28) **dC^{NMe3}TP**, **dA^{NH2}TP**, dGTP, **dU^{NMe}TP**; (lane 29) **dC^{NMe3}TP**, **dG^{NMe2}TP**, dTTP; (lane 30) **dC^{NMe3}TP**, **dA^{NH2}TP**, dTTP; (lane 31) **dA^{NH2}TP**, **dG^{NMe2}TP**, dTTP; (lane 32) **dC^{NMe3}TP**, **dA^{NH2}TP**, **dG^{NMe2}TP**, dTTP; (lane 33) **dC^{NMe3}TP**, dATP, **dG^{NMe2}TP**; (lane 34) **dC^{NMe3}TP**, dATP, **dU^{NMe}TP**; (lane 35) dATP, **dG^{NMe2}TP**, **dU^{NMe}TP**; (lane 36) **dC^{NMe3}TP**, dATP, **dG^{NMe2}TP**, **dU^{NMe}TP**; (lane 37) **dC^{NMe3}TP**, **dG^{NMe2}TP**, **dU^{NMe}TP**; (lane 38) **dC^{NMe3}TP**, **dA^{NH2}TP**, **dU^{NMe}TP**; (lane 39) **dC^{NMe3}TP**, **dA^{NH2}TP**, **dG^{NMe2}TP**; (lane 40) **dA^{NH2}TP**, **dG^{NMe2}TP**, **dU^{NMe}TP**; (lane 41) **dC^{NMe3}TP**, **dA^{NH2}TP**, **dG^{NMe2}TP**, **dU^{NMe}TP**.

2.4. PEX – Multiple incorporation (dA^{NH2}TP, dU^{NMe}TP, dG^{NMe2}TP, dC^{NMe3}TP in various template length)

Method B. The reaction mixture (10 µL) contained one of three templates (31-mer Temp^{Prb4basII} / 43-mer Temp^{MO43}/ 61-mer Temp^{MO61}) (3 µM, 0.75 µL), primer Prim^{248short}-FAM (3 µM, 0.5 µL), **dA^{NH2}TP** (0.25 mM, 1 µL), **dU^{NMe}TP** (2 mM, 1 µL), **dG^{NMe2}TP** (2 mM, 1 µL), **dC^{NMe3}TP** (2 mM, 1 µL), KOD XL DNA polymerase (0.6 U), and the enzyme reaction buffer (10X, 1 µL) as supplied by the manufacturer. Positive controls contained 0.3 U of KOD XL DNA polymerase and natural dNTPs (1 mM, 1 µL). The reaction mixture was incubated for 30 min at 72 °C, stopped by addition of PAGE stop solution (10 µL), and denatured for 5 min at 95 °C. Samples were analyzed on 20% PAGE and visualized using fluorescence imaging (Figure S4 – lanes 3-8). In attempts to remove the smearing of the bands, samples were additionally analyzed on 20% PAGE using 1X TBE buffer with pH 9.5 (Figure S5A – lanes 2-7), 10.5 (Figure S5B – lanes 2-7), and 11.9 (Figure S5C – lanes 2-7).

Method C. The reaction mixture (10 µL) contained 98-mer template Temp^{NK98} (3 µM, 0.75 µL), primer Prim^{NK98pr1}-FAM (3 µM, 0.5 µL), **dA^{NH2}TP** (0.25 mM, 1 µL), **dU^{NMe}TP** (2 mM, 1 µL), **dG^{NMe2}TP** (2 mM, 1 µL), **dC^{NMe3}TP** (2 mM, 1 µL), $MgSO_4$ (25 mM, 0.6 µL), KOD XL DNA polymerase (2 U), and the enzyme reaction buffer (10X, 1 µL) as supplied by the manufacturer. Positive control contained 0.35 U of KOD XL DNA polymerase and natural dNTPs (1 mM, 1 µL). The reaction mixture was incubated for 30 min at 72 °C, stopped by addition of PAGE stop solution (10 µL), and denatured for 5 min at 95 °C. Samples were analyzed on 20% PAGE and visualized using fluorescence imaging (Figure S4 – lanes 9-10). In attempts to remove the smearing of the bands, samples were additionally analyzed on 20% PAGE using 1X TBE buffer with pH 9.5 (Figure S5A – lanes 8-9), 10.5 (Figure S5B – lanes 8-9), and 11.9 (Figure S5C – lanes 8-9).

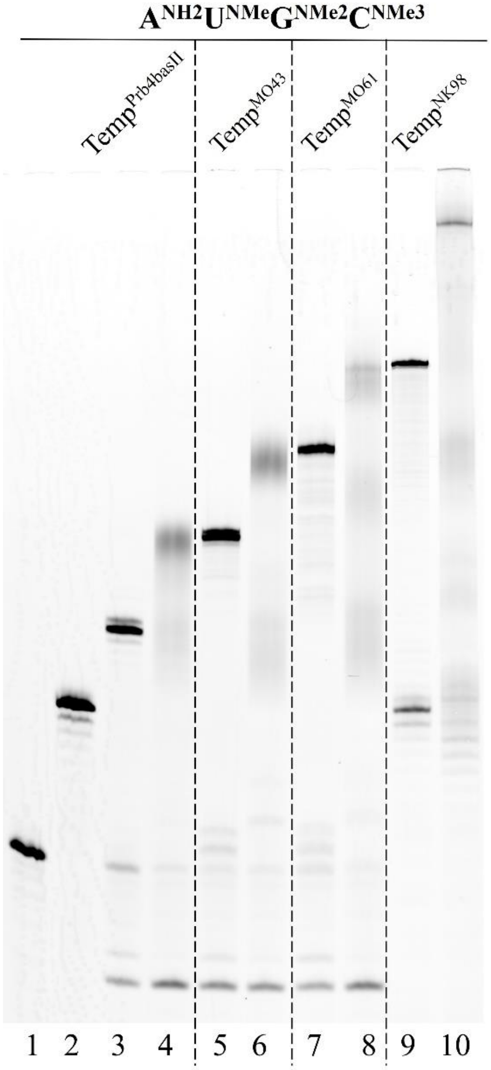

Figure S4. Denaturing PAGE analysis of PEX reactions with the set of four modified **dN^{R}TP**s (R = NH2, NMe, NMe2, NMe3; N = A, U, G, C) using KOD XL DNA polymerase and various templates: (lane 1) primer Prim^{248short}-FAM; (lane 2) primer Prim^{NK98pr1}-FAM; template Temp^{Prb4basII}: (lane 3) natural dNTPs; (lane 4) **dA^{NH2}TP**, **dU^{NMe}TP**, **dG^{NMe2}TP**, **dC^{NMe3}TP**; template Temp^{MO43}: (lane 5) natural dNTPs; (lane 6) **dA^{NH2}TP**, **dU^{NMe}TP**, **dG^{NMe2}TP**, **dC^{NMe3}TP**; template Temp^{MO61}: (lane 7) natural dNTPs; (lane 8) **dA^{NH2}TP**, **dU^{NMe}TP**, **dG^{NMe2}TP**, **dC^{NMe3}TP**; template Temp^{NK98}: (lane 9) natural dNTPs; (lane 10) **dA^{NH2}TP**, **dU^{NMe}TP**, **dG^{NMe2}TP**, **dC^{NMe3}TP**.

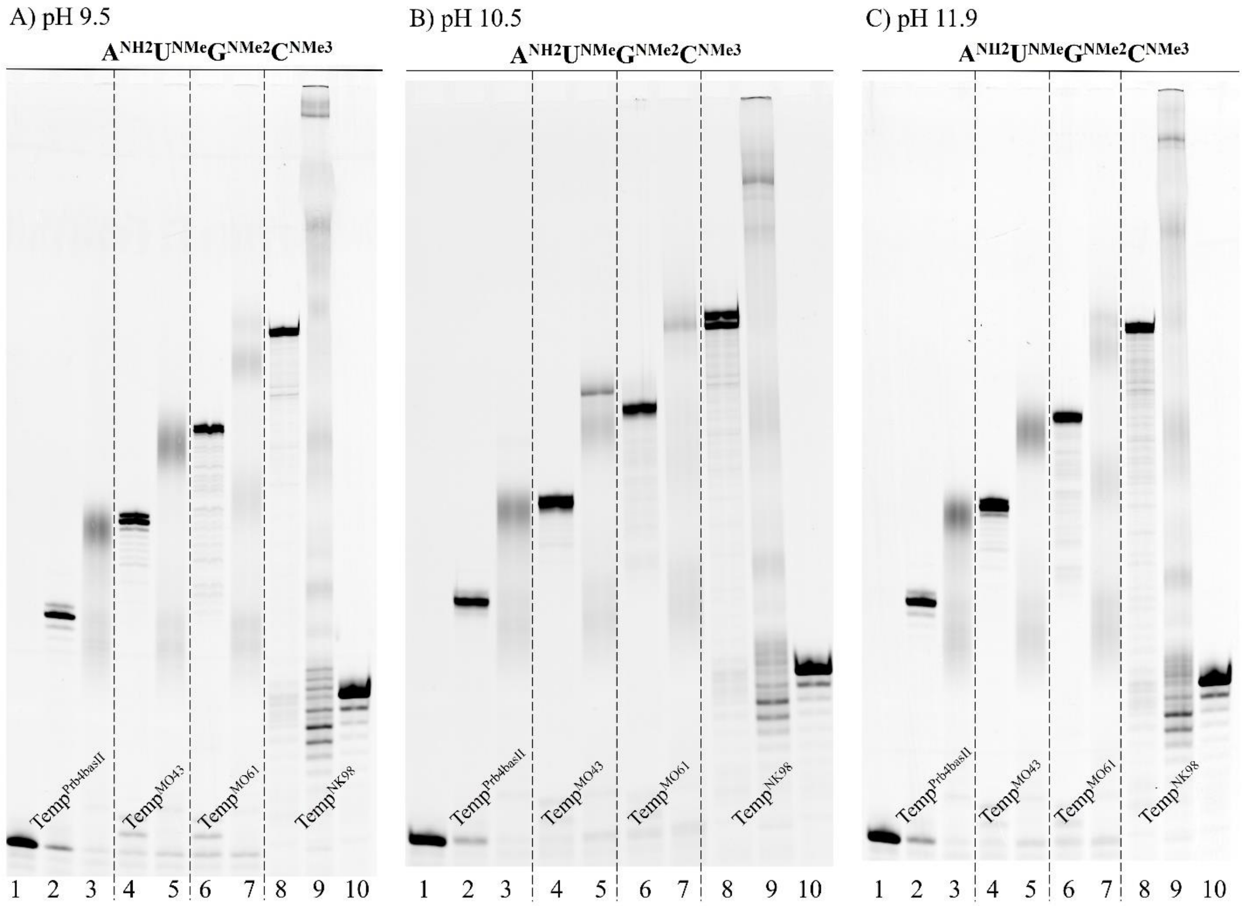

Figure S5. Denaturing PAGE analysis of PEX reactions using 1X TBE buffer with higher pH values: A) pH 9.5, B) pH 10.5, C) pH 11.9: (lane 1) primer Prim^{248short}-FAM; template Temp^{Prb4basII}: (lane 2) natural dNTPs; (lane 3) **dA^{NH2}TP**, **dU^{NMe}TP**, **dG^{NMe2}TP**, **dC^{NMe3}TP**; template Temp^{MO43}: (lane 4) natural dNTPs; (lane 5) **dA^{NH2}TP**, **dU^{NMe}TP**, **dG^{NMe2}TP**, **dC^{NMe3}TP**; template Temp^{MO61}: (lane 6) natural dNTPs; (lane 7) **dA^{NH2}TP**, **dU^{NMe}TP**,

$\mathbf{dG^{NMe2}TP}$, $\mathbf{dC^{NMe3}TP}$; template $Temp^{NK98}$: (lane 8) natural dNTPs; (lane 9) $\mathbf{dA^{NH2}TP}$, $\mathbf{dU^{NMe}TP}$, $\mathbf{dG^{NMe2}TP}$, $\mathbf{dC^{NMe3}TP}$; (lane 10) primer $Prim^{NK98pr1}$-FAM.

2.5. PEX with 98-mer template containing nucleotide repetitions

The reaction mixture (10 µL) contained 98-mer template $Temp^{FVL-A}$ (3 µM, 0.75 µL), primer $Prim^{LT25TH}$-FAM (3 µM, 0.5 µL), appropriate natural dNTPs (0.25 mM, 1 µL each), modified $\mathbf{dN^{R}TP}$ of study (R = NH2, NMe, NMe2, NMe3; N = A, U, G, C) (2 mM, 1 µL each), KOD XL DNA polymerase (units specification in Table S6) and a corresponding reaction buffer (10X, 1 µL) as supplied by the manufacturer. The positive control contained 0.3 U of KOD XL DNA polymerase and natural dNTPs (1 mM, 1 µL). The reaction mixture was incubated for 40 min at 60 °C, stopped by addition of PAGE stop solution (10 µL), and denatured for 5 min at 95 °C. Samples were analyzed on 12.5% PAGE and visualized using fluorescence imaging (Figure S6).

Table S6. Reaction condition specifications for multiple incorporation of $\mathbf{dN^{R}TP}$s using template $Temp^{FVL-A}$.

$\mathbf{dN^{R}TP}$	KOD XL
$\mathbf{dA^{NH2}TP}$	0.01 U
$\mathbf{dU^{NMe}TP}$	0.2 U
$\mathbf{dG^{NMe2}TP}$	0.5 U
$\mathbf{dC^{NMe3}TP}$	0.6 U

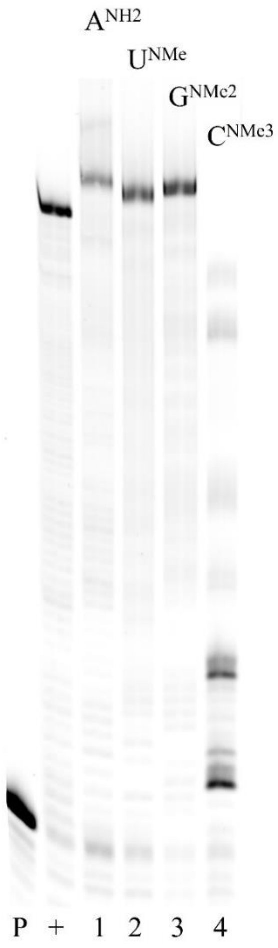

Figure S6. Denaturing PAGE analysis of PEX reaction with one modified **dN^{R}TP** using KOD XL DNA polymerase and template Temp$^{FVL\text{-}A}$: (P) primer Prim^{LT25TH}-FAM; (+) natural dNTPs; (lane 1) dCTP, **dA^{NH2}TP**, dGTP, dTTP; (lane 2) dCTP, dATP, dGTP, **dU^{NMe}TP**; (lane 3) dCTP, dATP, **dG^{NMe2}TP**, dTTP; (lane 4) **dC^{NMe3}TP**, dATP, dGTP, dTTP.

2.6. PEX – Multiple incorporation (dA^{NH2}TP, dU^{EPh}TP, dG^{PA}TP, dC^{EAlk}TP in various template length)

Method D. The reaction mixture (10 µL) contained one of three templates (31-mer Temp^{Prb4basII} / 43-mer Temp^{MO43}/ 61-mer Temp^{MO61}) (3 µM, 0.75 µL), primer Prim^{248short}-FAM (3 µM, 0.5 µL), **dA^{NH2}TP** (0.25 mM, 1 µL), **dU^{EPh}TP** (2 mM, 1 µL), **dG^{PA}TP** (2 mM, 1 µL), **dC^{EAlk}TP** (2 mM, 1 µL), KOD XL DNA polymerase (0.1 U), and the enzyme reaction buffer (10X, 1 µL) as supplied by the manufacturer. Positive controls contained 0.3 U of KOD XL DNA polymerase and natural dNTPs (1 mM, 1 µL). The reaction mixture was incubated for 30 min at 72 °C, stopped by addition

of PAGE stop solution (10 µL), and denatured for 5 min at 95 °C. Samples were analyzed on 20% PAGE and visualized using fluorescence imaging (Figure S7 – lanes 2-7).

Method E. The reaction mixture (10 µL) contained 98-mer template Temp^{NK98} (3 µM, 0.75 µL), primer Prim^{NK98pr1}-FAM (3 µM, 0.5 µL), **dA^{NH2}TP** (0.25 mM, 1 µL), **dU^{EPh}TP** (2 mM, 1 µL), **dG^{PA}TP** (2 mM, 1 µL), **dC^{EAlk}TP** (2 mM, 1 µL), KOD XL DNA polymerase (0.2 U), and the enzyme reaction buffer (10X, 1 µL) as supplied by the manufacturer. Positive control contained 0.35 U of KOD XL DNA polymerase and natural dNTPs (1 mM, 1 µL). The reaction mixture was incubated for 30 min at 60 °C, stopped by addition of PAGE stop solution (10 µL), and denatured for 5 min at 95 °C. Samples were analyzed on 20% PAGE and visualized using fluorescence imaging (Figure S7 – lanes 8,9).

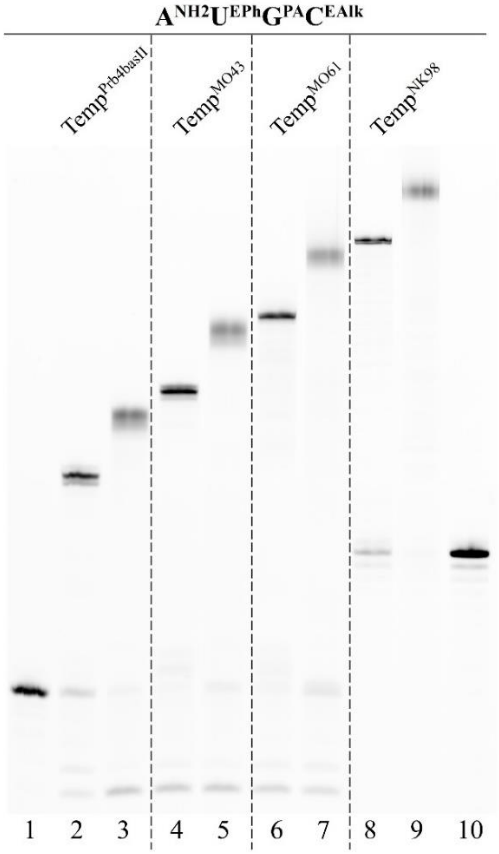

Figure S7. Denaturing PAGE analysis of PEX reactions with the set of four modified **dN^{R}TP**s (R = NH2, EPh, PA, EAlk; N = A, U, G, C) using KOD XL DNA polymerase and various templates: (lane 1) primer Prim^{248short}-FAM; template Temp^{Prb4basII}: (lane 2) natural dNTPs; (lane 3) **dA^{NH2}TP**, **dU^{EPh}TP**, **dG^{PA}TP**, **dC^{EAlk}TP**; template Temp^{MO43}: (lane 4) natural dNTPs;

(lane 5) **dA^{NH2}TP**, **dU^{EPh}TP**, **dG^{PA}TP**, **dC^{EAlk}TP**; template Temp^{MO61}: (lane 6) natural dNTPs; (lane 7) **dA^{NH2}TP**, **dU^{EPh}TP**, **dG^{PA}TP**, **dC^{EAlk}TP**; template Temp^{NK98}: (lane 8) natural dNTPs; (lane 9) **dA^{NH2}TP**, **dU^{EPh}TP**, **dG^{PA}TP**, **dC^{EAlk}TP**; (lane 10) primer Prim^{NK98pr1}-FAM.

2.7. Sample preparation for mass spectrometry analysis

Method F. In order to obtain sufficient amounts of **19DNA_A^{NH2}**, **19DNA_U^{NMe}**, **19DNA_G^{NMe2}**, and **19DNA_C^{NMe3}** (for sequences see Table S2), corresponding PEX reactions (described in section 2.1) were ten times scaled up. The reactions were stopped by cooling to 8 °C, purified using QIAquick Nucleotide Removal Kit, concentrated to 10 µL, and analyzed by UHPLC-MS (for results see Table S8). For copies of ESI spectra go to section 5, Figures S21-S28.

Method G. In order to obtain sufficient amounts of **31ON_A^{NH2}**, **31ON_U^{NMe}**, **31ON_G^{NMe2}**, **31ON_C^{NMe3}**, **31ON_A^{NH2}U^{NMe}G^{NMe2}C^{NMe3}**, and **31ON_A^{NH2}U^{EPh}G^{PA}C^{EAlk}** (for sequences see Table S2), the reaction mixture (50 µL) containing dual-biotinylated template Temp^{Prb4basII}-bio, primer Prim^{248short}-FAM, appropriate natural dNTPs (4 mM, 2.5 µL) and modified **dN^{R}TP**s (4 mM, 2.5 µL; only in the case of **dC^{NMe3}TP** 6 mM), $MgSO_4$ (if needed, specified in Table S7), KOD XL DNA polymerase (all conditions are specified in Table S7), and the enzyme reaction buffer (10X, 5 µL) as supplied by the manufacturer was incubated either at 60 or 72 °C (temperature and time are specified in Table S7). The reaction was stopped by cooling to 8 °C. The products were purified using the DBStv magnetoseparation procedure followed by UHPLC-MS analysis (Table S8). For copies of ESI spectra go to section 5, Figures S29-S36, S35-S36. In the case of **31ON_A^{NH2}U^{NMe}G^{NMe2}C^{NMe3}** it was not possible to obtain the mass by UHPLC-MS, instead, MALDI-TOF analysis was used (Table S8, Figure S37).

DBStv magnetoseparation procedure. Streptavidin particles (50 µL) were washed with binding buffer (3 × 200 µL; 10 mM Tris, 1 mM EDTA, 100 mM NaCl, pH 7.5). 50 µL of PEX solutions were mixed with binding buffer (200 µL) and incubated for 30 min at 15 °C and 1400 rpm. The magnetic beads were collected on a magnet (DynaMagTM-2, Invitrogen), and washed with washing buffer (3 × 200 µL; 10 mM Tris, 1 mM EDTA, 500 mM NaCl, pH 7.5) and water (3 × 200 µL). Then water (50 µL) was added and the sample was denatured for 5 min at 900 rpm and 75 °C. The beads were collected on a magnet and the DNA containing solution was transferred into a clean vial.

Table S7. Reaction condition specifications for Method G.

ON name	**dN^{R}TP**s	Template	Primer	Reaction time	Temperature	KOD XL	$MgSO_4$
**31ON_A^{NH2}**	**dA^{NH2}TP**	150 pmol	150 pmol	40 min	60 °C	0.5 U	–
**31ON_U^{NMe}**	**dU^{NMe}TP**					1.25 U	
**31ON_G^{NMe2}**	**dG^{NMe2}TP**					0.5 U	
**31ON_C^{NMe3}**	**dC^{NMe3}TP**	75 pmol	75 pmol	60 min	72 °C	2.5 U	1.5 mM
**31ON_A^{NH2}U^{NMe}G^{NMe2}C^{NMe3}**	**dA^{NH2}TP**, **dU^{NMe}TP**, **dG^{NMe2}TP**, **dC^{NMe3}TP**					2.5 U	1.5 mM
**31ON_A^{NH2}U^{EPh}G^{PA}C^{EAlk}**	**dA^{NH2}TP**, **dU^{EPh}TP**, **dG^{PA}TP**, **dC^{EAlk}TP**			30 min		1 U	–

2.8. UHPLC-MS and MALDI-TOF measurements

Table S8. Overview of modified ssONs and their masses measured by UHPLC-MS (MALDI-TOF in the case of **31ON_A^{NH2}U^{NMe}G^{NMe2}C^{NMe3}**).

ON name	Mass calculated [Da]	Mass found [Da]	Δ [Da]	Figure number
**19ON_A^{NH2}** ^{a}	6605.9	6605	0.9	S21, S22
**19ON_U^{NMe}** ^{a}	6555.9	6555	0.9	S23, S24
**19ON_G^{NMe2}** ^{a}	6532.9	6532	0.9	S25, S26
**19ON_C^{NMe3}** ^{a}	6583.9	6582	1.9	S27, S28
**31ON_A^{NH2}** ^{a}	10530.3	10530	0.3	S29, S30
**31ON_U^{NMe}** ^{a}	10366.3	10360	6.3	S31, S32
**31ON_G^{NMe2}** ^{a}	10474.3	10470	4.3	S33, S34
**31ON_C^{NMe3}** ^{a}	10538.3	10530	8.3	S35, S36
**31ON_A^{NH2}U^{NMe}G^{NMe2}C^{NMe3}** ^{a}	11446.3	11446.9	0.6	S37
**31ON_A^{NH2}U^{EPh}G^{PA}C^{EAlk}** ^{a}	11662.3	11660	2.3	S38, S39

Note: ^{a} 5'-(6-FAM)-labelled extended strand after PEX

2.9. PCR – Multiple incorporation (one modified dN^{R}TP)

The reaction mixture (10 µL) contained template Temp^{NK98} (0.5 µM, 0.5 µL), reverse primer Prim^{NK98pr1}-FAM and forward primer Prim^{NK98pr2}-Cy5 (10 µM, 1 µL each), appropriate set of natural dNTPs (2 mM, 1 µL), one of the modified **dN^{R}TP**s (R = NH2, NMe, NMe2, NMe3; N = A, U, G, C) (1 µL, conditions specified in Table S9), suitable additives, KOD XL DNA polymerase (Table S9), and a corresponding reaction buffer (10X, 1 µL) as supplied by the manufacturer. The positive control contained 0.1 U of KOD XL DNA polymerase and natural dNTPs (2 mM, 2 µL). All reaction mixtures were under cycling protocol: 94 °C for 5 min, followed by 30 cycles at 94 °C for 1 min, 53 °C for 30 sec, and 72 °C for 2 min, followed by a final elongation step at 72 °C for

5 min. Samples were analyzed on 12.5% PAGE and agarose gel electrophoresis and visualized using fluorescence imaging (Figure S8).

Table S9. Reaction condition specifications for PCR (one modified **dN^{R}TP**).

Entry	**dN^{R}TP**, mM	KOD XL, U	Additives	PCR product
1	**dA^{NH2}TP**, 2 mM	0.5 U	5% formamide	**98PCR_A^{NH2}**
2	**dU^{NMe}TP**, 4 mM	2 U	1.5 mM $MgSO_4$	**98PCR_U^{NMe}**
3	**dG^{NMe2}TP**, 2 mM	0.5 U	1 M betaine	**98PCR_G^{NMe2}**
4	**dC^{NMe3}TP**, 2 mM	1.5 U	1.5 M betaine	**98PCR_C^{NMe3}**

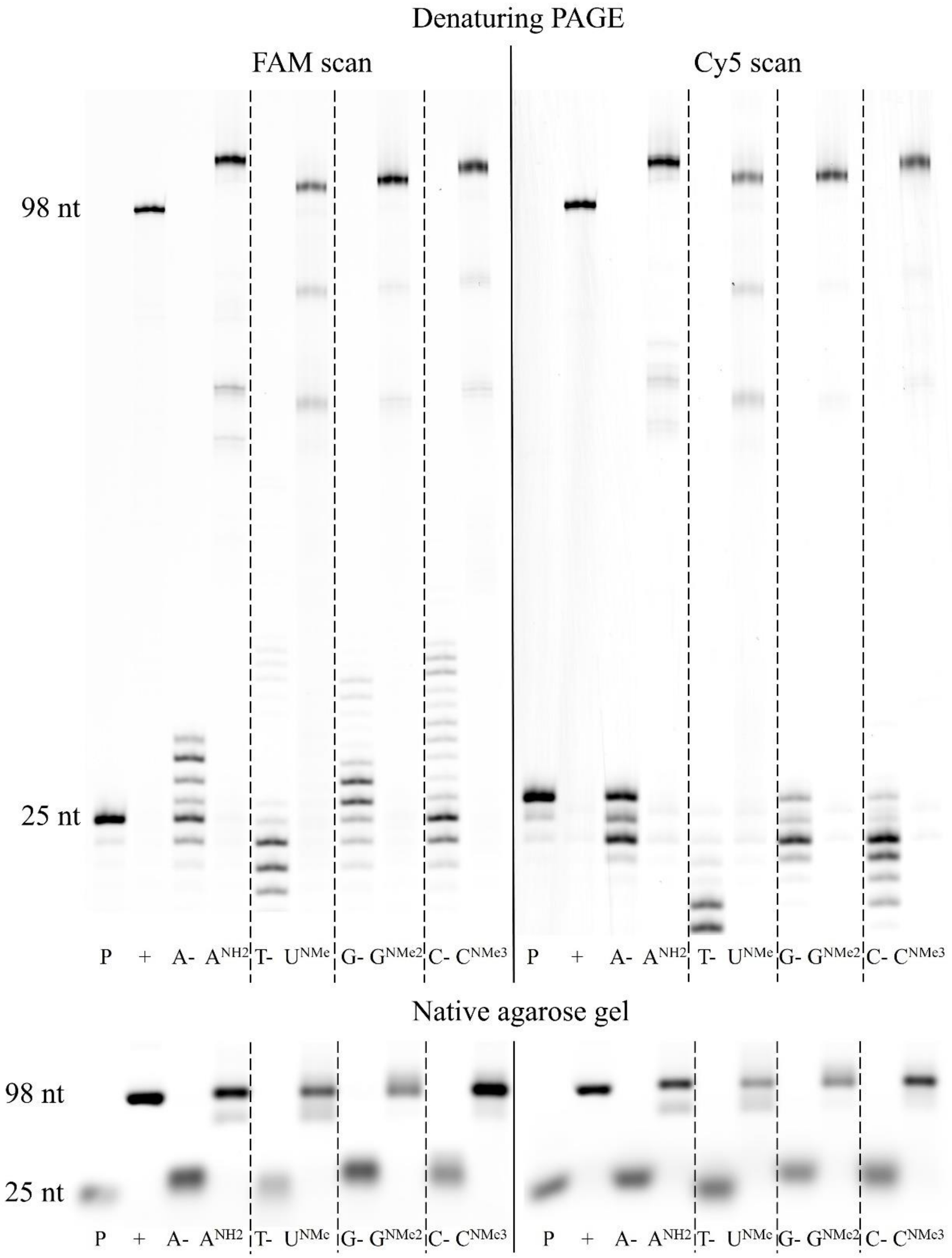

Figure S8. Denaturing PAGE (upper) and native agarose (lower) gel analysis of PCR reactions with one modified **dN^R^TP** using 98-mer template Temp^{NK98} and KOD XL DNA polymerase: (P) primer; (+) natural dNTPs; (A-) dCTP, dGTP, dTTP; (A^{NH2}) dCTP, **dA^{NH2}TP**, dGTP, dTTP; (T-) dCTP, dATP, dGTP; (U^{NMe}) dCTP, dATP, dGTP, **dU^{NMe}TP**; (G-) dCTP, dATP, dTTP;

(G^{NMe2}) dCTP, dATP, **dG^{NMe2}TP**, dTTP; (C-) dATP, dGTP, dTTP; (C^{NMe3}) **dC^{NMe3}TP**, dATP, dGTP, dTTP.

2.10. PCR – Multiple incorporation (two modified dN^{R}TPs)

The reaction mixture (10 µL) contained template Temp^{NK98} (0.5 µM, 0.5 µL), reverse primer Prim^{NK98pr1}-FAM and forward primer Prim^{NK98pr2}-Cy5 (10 µM, 1 µL each), appropriate set of two natural dNTPs (2 mM, 1 µL), two modified **dN^{R}TP**s (R = NH2, NMe, NMe2, NMe3; N = A, U, G, C) (2 mM, 1 µL each), additives (specifications in Table S10), KOD XL DNA polymerase (specifications in Table S10) and a corresponding reaction buffer (10X, 1 µL) as supplied by the manufacturer. The positive control contained 0.1 U of KOD XL DNA polymerase and natural dNTPs (2 mM, 1 µL). All reaction mixtures were under cycling protocol: 94 °C for 5 min, followed by 30 cycles at 94 °C for 1 min, 53 °C for 30 min, and 72 °C for either 2 or 5 min (elongation time is specified in Table S10), followed by a final elongation step at 72 °C for 5 min. Samples were analyzed on 12.5% PAGE and visualized using fluorescence imaging (Figure S9).

Table S10. Reaction condition specifications for PCR (two modified **dN^{R}TP**s).

Lanes in Figure S9	**dN^{R}TP**s	KOD XL, U	Additives	Elongation step, min	PCR product
5	**dA^{NH2}TP**, **dU^{NMe}TP**	1.5 U	5% formamide, 1.5 mM $MgSO_4$	2	**98PCR_A^{NH2}U^{NMe}**
8	**dA^{NH2}TP**, **dG^{NMe2}TP**	1 U	1 M betaine	2	**98PCR_A^{NH2}G^{NMe2}**
11	**dA^{NH2}TP**, **dC^{NMe3}TP**	3 U	1.5 M betaine	5	**98PCR_A^{NH2}C^{NMe3}**
14	**dU^{NMe}TP**, **dG^{NMe2}TP**	1.5 U	–	2	**98PCR_U^{NMe}G^{NMe2}**
17	**dU^{NMe}TP**, **dC^{NMe3}TP**	1.5 U	1.5 M betaine, 1.5 mM $MgSO_4$	5	**98PCR_U^{NMe}C^{NMe3}**
20	**dG^{NMe2}TP**, **dC^{NMe3}TP**	2 U	1.7 M betaine	5	**98PCR_G^{NMe2}C^{NMe3}**

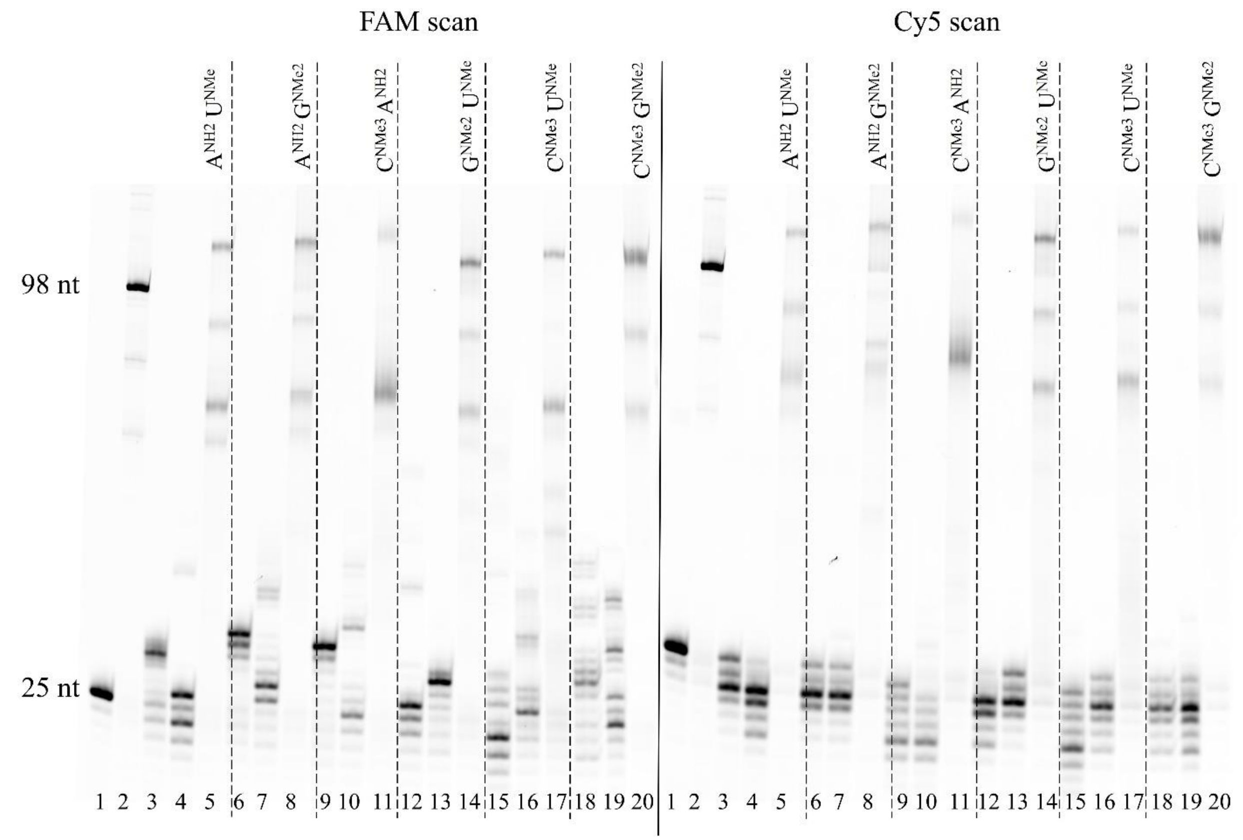

Figure S9. Denaturing PAGE analysis of PCR reactions with two modified **dN^{R}TP**s using 98-mer template Temp^{NK98} and KOD XL DNA polymerase: (lane 1) primer; (lane 2) natural dNTPs; (lane 3) dCTP, dGTP, **dU^{NMe}TP**; (lane 4) dCTP, **dA^{NH2}TP**, dGTP; (lane 5) dCTP, **dA^{NH2}TP**, dGTP, **dU^{NMe}TP**; (lane 6) dCTP, **dG^{NMe2}TP**, dTTP; (lane 7) dCTP, **dA^{NH2}TP**, dTTP; (lane 8) dCTP, **dA^{NH2}TP**, **dG^{NMe2}TP**, dTTP; (lane 9) **dC^{NMe3}TP**, dGTP, dTTP; (lane 10) **dA^{NH2}TP**, dGTP, dTTP; (lane 11) **dC^{NMe3}TP**, **dA^{NH2}TP**, dGTP, dTTP; (lane 12) dCTP, dATP, **dG^{NMe2}TP**; (lane 13) dCTP, dATP, **dU^{NMe}TP**; (lane 14) dCTP, dATP, **dG^{NMe2}TP**, **dU^{NMe}TP**; (lane 15) **dC^{NMe3}TP**, dATP, dGTP; (lane 16) dATP, dGTP, **dU^{NMe}TP**; (lane 17) **dC^{NMe3}TP**, dATP, dGTP, **dU^{NMe}TP**; (lane 18) **dC^{NMe3}TP**, dATP, dTTP; (lane 19) dATP, **dG^{NMe2}TP**, dTTP; (lane 20) **dC^{NMe3}TP**, dATP, **dG^{NMe2}TP**, dTTP.

2.11. PCR – Multiple incorporation (three and four modified dN^{R}TPs)

Method H. The reaction mixture (10 µL) contained template Temp^{NK98} (0.5 µM, 0.5 µL), reverse primer Prim^{NK98pr1}-FAM and forward primer Prim^{NK98pr2}-Cy5 (10 µM, 1 µL each), an appropriate natural dNTP (2 mM, 1 µL), three modified **dN^{R}TP**s (R = NH2, NMe, NMe2, NMe3;

N = A, U, G, C) (2 mM, 1 µL each), additives (specifications in Table S11), KOD XL DNA polymerase (specifications in Table S11) and a corresponding reaction buffer (10X, 1 µL) as supplied by the manufacturer. The positive control contained 0.1 U of KOD XL DNA polymerase and natural dNTPs (2 mM, 1 µL). All reaction mixtures were under cycling protocol: 94 °C for 5 min, followed by 30 cycles at 94 °C for 1 min, 53 °C for 30 min, and 72 °C for 5 min, followed by a final elongation step at 72 °C for 5 min. Samples were analyzed on 12.5% PAGE and visualized using fluorescence imaging (Figure S10 – lanes 3-6).

Method I. The reaction mixture (10 µL) contained template Temp^{NK98} (0.5 µM, 0.5 µL), reverse primer Prim^{NK98pr1}-FAM and forward primer Prim^{NK98pr2}-Cy5 (10 µM, 1 µL each), set of modified **dN^{R}TP**s (2 mM, 1 µL each) – **dA^{NH2}TP**, **dU^{NMe}TP**, **dG^{NMe2}TP**, **dC^{NMe3}TP** or **dA^{NH2}TP**, **dU^{EPh}TP**, **dG^{PA}TP**, **dC^{EAlk}TP**, additives (specifications in Table S11), KOD XL DNA polymerase (specifications in Table S11) and a corresponding reaction buffer (10X, 1 µL) as supplied by the manufacturer. The positive control contained 0.1 U of KOD XL DNA polymerase and natural dNTPs (2 mM, 1 µL). All reaction mixtures were under cycling protocol: 94 °C for 5 min, followed by 30 cycles at 94 °C for 1 min, 53 °C for 30 min, and 72 °C for 5 min, followed by a final elongation step at 72 °C for 5 min. Samples were analyzed on 12.5% PAGE and visualized using fluorescence imaging (Figure S10 – lanes 7-8).

Table S11. Reaction condition specifications for PCR (three and four modified **dN^{R}TP**s).

Lanes in Figure S10	**dN^{R}TP**s	KOD XL	Additives	PCR product
3	**dA^{NH2}TP**, **dU^{NMe}TP**, **dG^{NMe2}TP**	1.5 U	1 M betaine	**98PCR_A^{NH2}U^{NMe}G^{NMe2}**
4	**dA^{NH2}TP**, **dU^{NMe}TP**, **dC^{NMe3}TP**	3 U	1.5 mM $MgSO_4$, 1.5 M betaine	**98ON_A^{NH2}U^{NMe}C^{NMe3}**
5	**dA^{NH2}TP**, **dG^{NMe2}TP**, **dC^{NMe3}TP**	3 U	1.7 M betaine	**98PCR_A^{NH2}G^{NMe2}C^{NMe3}**
6	**dU^{NMe}TP**, **dG^{NMe2}TP**, **dC^{NMe3}TP**	2 U	1.7 M betaine	**98PCR_U^{NMe}G^{NMe2}C^{NMe3}**
7	**dA^{NH2}TP**, **dU^{NMe}TP**, **dG^{NMe2}TP**, **dC^{NMe3}TP**	3 U	1.5 M betaine	**no product**
8	**dA^{NH2}TP**, **dU^{EPh}TP**, **dG^{PA}TP**, **dC^{EAlk}TP**	0.5 U	5% formamide	**98PCR_ A^{NH2}U^{EPh}G^{PA}C^{EAlk}**

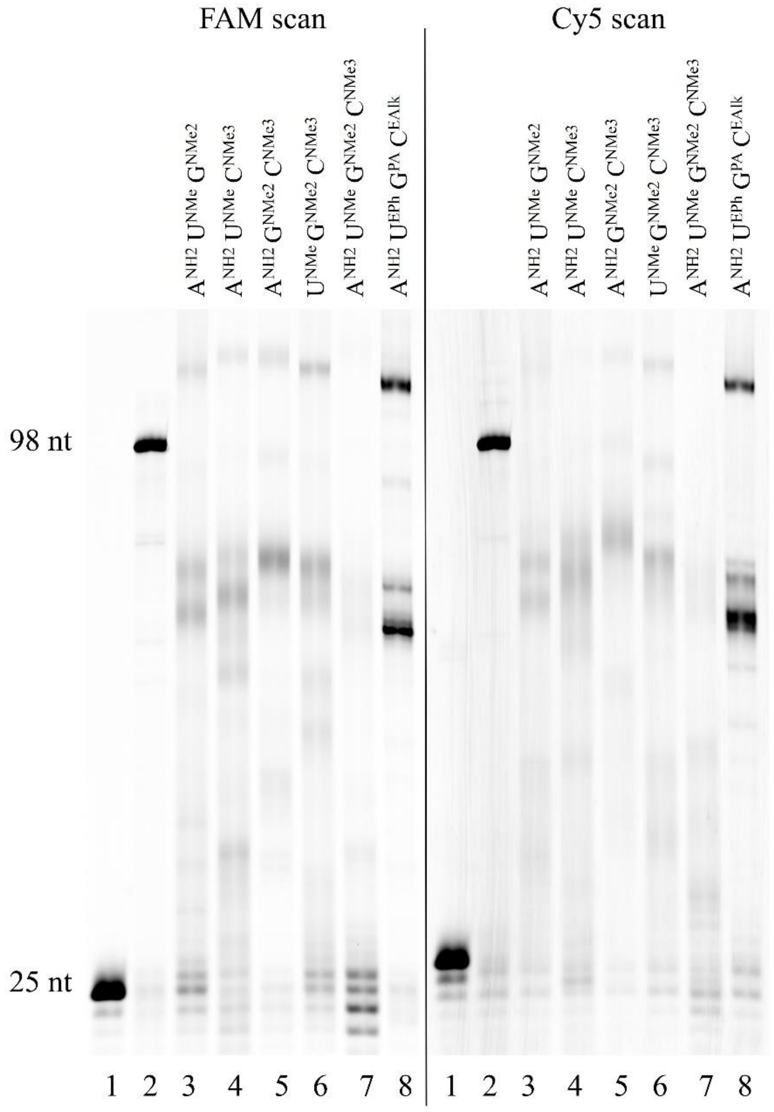

Figure S10. Denaturing PAGE analysis of PCR reactions with three and four modified **dN$^{\mathbf{R}}$TP**s using 98-mer template Temp^{NK98} and KOD XL DNA polymerase: (lane 1) primer; (lane 2) natural dNTPs; (lane 3) **dA$^{\mathbf{NH2}}$TP**, **dU$^{\mathbf{NMe}}$TP**, **dG$^{\mathbf{NMe2}}$TP**, dCTP; (lane 4) **dA$^{\mathbf{NH2}}$TP**, **dU$^{\mathbf{NMe}}$TP**, dGTP, **dC$^{\mathbf{NMe3}}$TP**; (lane 5) **dA$^{\mathbf{NH2}}$TP**, dTTP, **dG$^{\mathbf{NMe2}}$TP**, **dC$^{\mathbf{NMe3}}$TP**; (lane 6) dATP, **dU$^{\mathbf{NMe}}$TP**, **dG$^{\mathbf{NMe2}}$TP**, **dC$^{\mathbf{NMe3}}$TP**; (lane 7) **dA$^{\mathbf{NH2}}$TP**, **dU$^{\mathbf{NMe}}$TP**, **dG$^{\mathbf{NMe2}}$TP**, **dC$^{\mathbf{NMe3}}$TP**; (lane 8) **dA$^{\mathbf{NH2}}$TP**, **dU$^{\mathbf{EPh}}$TP**, **dG$^{\mathbf{PA}}$TP**, **dC$^{\mathbf{EAlk}}$TP**.

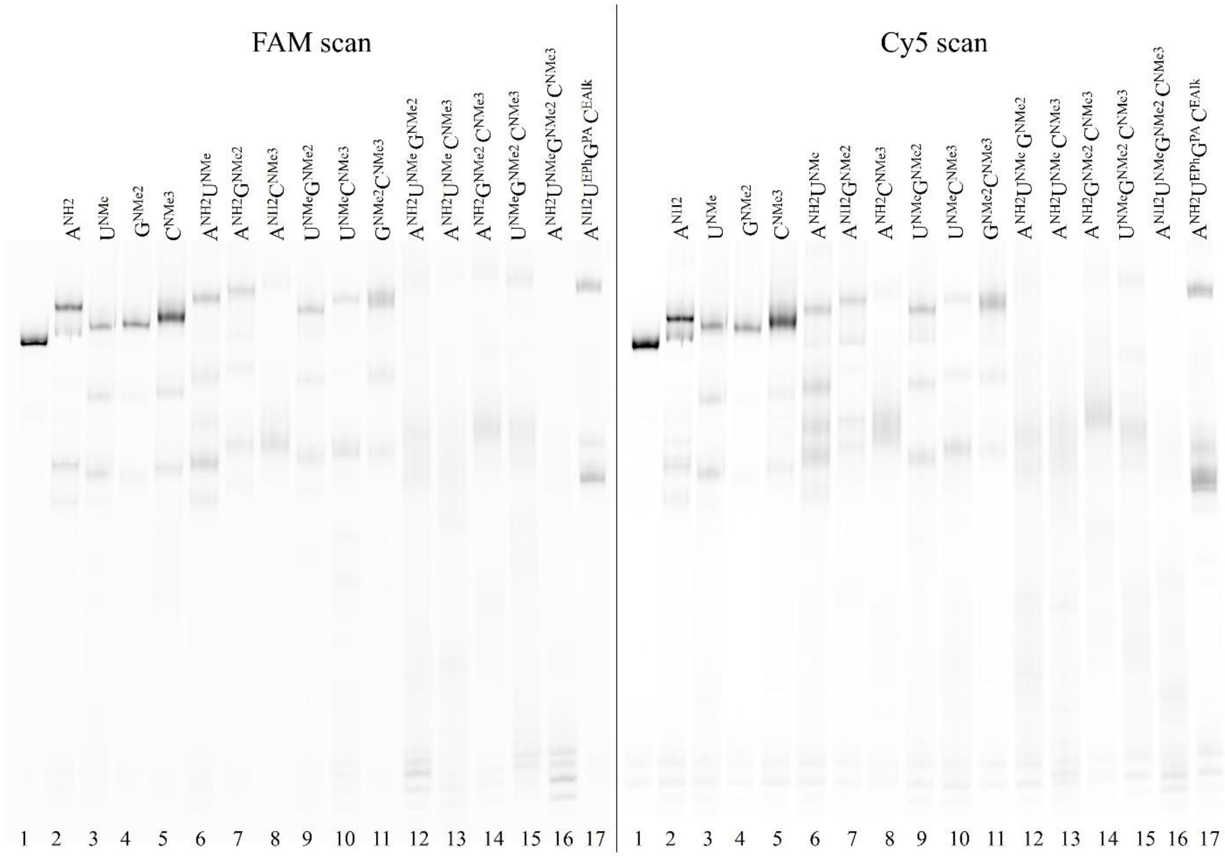

Figure S11. The unmodified copy of Figure 3B containing: (lane 1) natural dNTPs; (lane 2) dCTP, **dA^{NH2}TP**, dGTP, dTTP; (lane 3) dCTP, dATP, dGTP, **dU^{NMe}TP**; (lane 4) dCTP, dATP, **dG^{NMe2}TP**, dTTP; (lane 5) **dC^{NMe3}TP**, dATP, dGTP, dTTP; (lane 6) dCTP, **dA^{NH2}TP**, dGTP, **dU^{NMe}TP**; (lane 7) dCTP, **dA^{NH2}TP**, **dG^{NMe2}TP**, dTTP; (lane 8) **dC^{NMe3}TP**, **dA^{NH2}TP**, dGTP, dTTP; (lane 9) dCTP, dATP, **dG^{NMe2}TP**, **dU^{NMe}TP**; (lane 10) **dC^{NMe3}TP**, dATP, dGTP, **dU^{NMe}TP**; (lane 11) **dC^{NMe3}TP**, dATP, **dG^{NMe2}TP**, dTTP; (lane 12) **dA^{NH2}TP**, **dU^{NMe}TP**, **dG^{NMe2}TP**, dCTP; (lane 13) **dA^{NH2}TP**, **dU^{NMe}TP**, dGTP, **dC^{NMe3}TP**; (lane 14) **dA^{NH2}TP**, dTTP, **dG^{NMe2}TP**, **dC^{NMe3}TP**; (lane 15) dATP, **dU^{NMe}TP**, **dG^{NMe2}TP**, **dC^{NMe3}TP**; (lane 16) **dA^{NH2}TP**, **dU^{NMe}TP**, **dG^{NMe2}TP**, **dC^{NMe3}TP**; (lane 17) **dA^{NH2}TP**, **dU^{EPh}TP**, **dG^{PA}TP**, **dC^{EAlk}TP**.

2.12. aPCR – Multiple incorporation (four modified dN^{R}TPs)

The reaction mixture (10 µL) contained template Temp^{NK98} (5 µM, 0.5 µL), primer Prim^{NK98pr1}-FAM (10 µM, 1 µL), set of modified **dN^{R}TPs** (2 mM, 1 µL each) – **dA^{NH2}TP**, **dU^{NMe}TP**, **dG^{NMe2}TP**, **dC^{NMe3}TP** or **dA^{NH2}TP**, **dU^{EPh}TP**, **dG^{PA}TP**, **dC^{EAlk}TP**, additives (specifications in Table S12), KOD XL DNA polymerase (specifications in Table S12) and a

corresponding reaction buffer (10X, 1 μL) as supplied by the manufacturer. The positive control contained 0.1 U of KOD XL DNA polymerase and natural dNTPs (2 mM, 1 μL). All reaction mixtures were under cycling protocol: 94 °C for 5 min, followed by 30 cycles at 94 °C for 1 min, 53 °C for 30 min, and 72 °C for 10 min, followed by a final elongation step at 72 °C for 5 min. Samples were analyzed on 12.5% PAGE and visualized using fluorescence imaging (Figure S12).

Table S12. Reaction condition specifications for aPCR (four modified **dN^{R}TP**s).

Lanes in Figure S12	dN^{R}TPs	KOD XL	Additives	aPCR product
7	**dA^{NH2}TP**, **dU^{NMe}TP**, **dG^{NMe2}TP**, **dC^{NMe3}TP**	2 U	1.5 M betaine	**98ON_A^{NH2}U^{NMe}G^{NMe2}C^{NMe3}**
12	**dA^{NH2}TP**, **dU^{EPh}TP**, **dG^{PA}TP**, **dC^{EAlk}TP**	0.5 U	5% formamide	**98ON_A^{NH2}U^{EPh}G^{PA}C^{EAlk}**

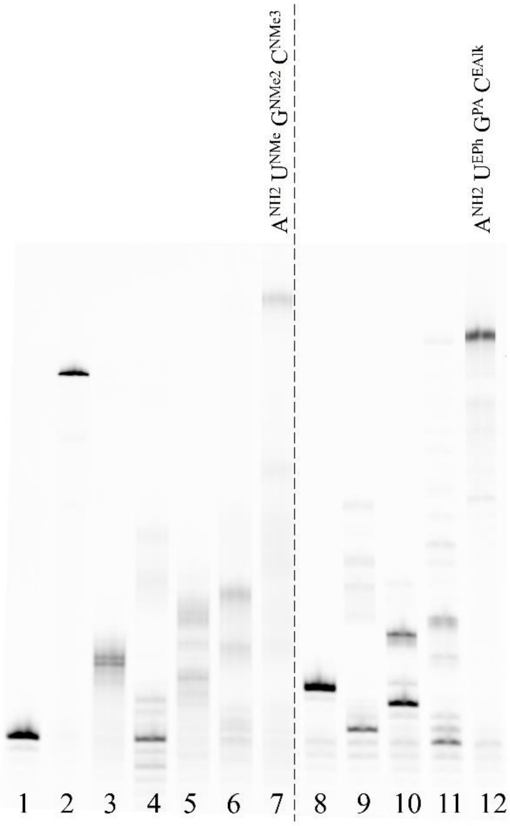

Figure S12. Denaturing PAGE analysis of aPCR reactions with four modified **dN^{R}TP**s using 98-mer template Temp^{NK98} and KOD XL DNA polymerase: (lane 1) primer Prim^{NK98pr1}-FAM;

(lane 2) natural dNTPs; (lane 3) **dU^{NMe}TP**, **dG^{NMe2}TP**, **dC^{NMe3}TP**; (lane 4) **dA^{NH2}TP**, **dG^{NMe2}TP**, **dC^{NMe3}TP**; (lane 5) **dA^{NH2}TP**, **dU^{NMe}TP**, **dC^{NMe3}TP**; (lane 6) **dA^{NH2}TP**, **dU^{NMe}TP**, **dG^{NMe2}TP**; (lane 7) **dA^{NH2}TP**, **dU^{NMe}TP**, **dG^{NMe2}TP**, **dC^{NMe3}TP**; (lane 8) **dU^{EPh}TP**, **dG^{PA}TP**, **dC^{EAlk}TP**; (lane 9) **dA^{NH2}TP**, **dG^{PA}TP**, **dC^{EAlk}TP**; (lane 10) **dA^{NH2}TP**, **dU^{EPh}TP**, **dC^{EAlk}TP**; (lane 11) **dA^{NH2}TP**, **dU^{EPh}TP**, **dG^{PA}TP**; (lane 12) **dA^{NH2}TP**, **dU^{EPh}TP**, **dG^{PA}TP**, **dC^{EAlk}TP**.

2.13. Nuclease degradation experiments

For nuclease degradation experiments four 5'-(6-FAM)-labelled 31-mer ONs were prepared – **31ON**, **31ON_C^{CA}G^{PA}U^{SA}A^{OP}**, **31ON_A^{NH2}U^{NMe}G^{NMe2}C^{NMe3}**, and **31ON_A^{NH2}U^{EPh}G^{PA}C^{EAlk}** (for sequences see Table S2). **31ON** is a non-modified ON, **31ON_C^{CA}G^{PA}U^{SA}A^{OP}** is a fully-modified ON bearing following anionic modifications:

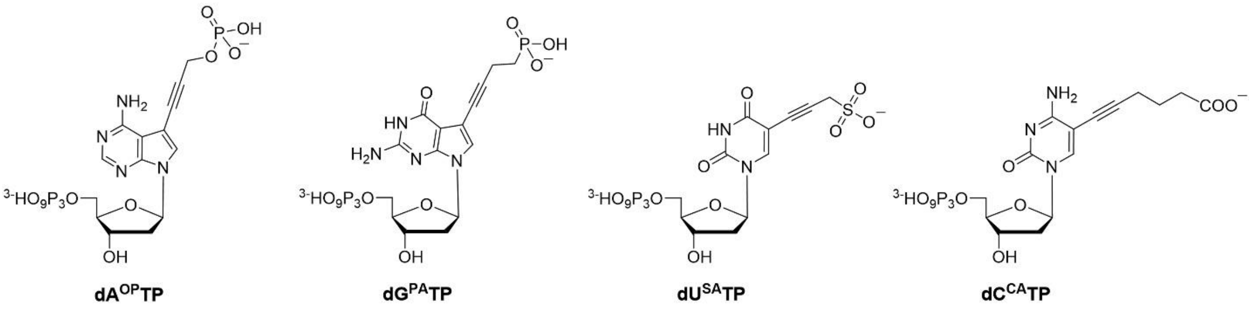

The synthesis of **31ON** and **31ON_C^{CA}G^{PA}U^{SA}A^{OP}** was reported previously[6]. The preparation of **31ON_A^{NH2}U^{NMe}G^{NMe2}C^{NMe3}** and **31ON_A^{NH2}U^{EPh}G^{PA}C^{EAlk}** is described in section 2.7.

To conduct nuclease degradation experiments, four 5'-(6-FAM)-labelled 31-mer ONs (0.1 µg) – **31ON**, **31ON_C^{CA}G^{PA}U^{SA}A^{OP}**, **31ON_A^{NH2}U^{NMe}G^{NMe2}C^{NMe3}**, and **31ON_A^{NH2}U^{EPh}G^{PA}C^{EAlk}** were mixed with DNase I (0.01 U) and the supplied reaction buffer (10X, 1 µL) and further diluted to total volume of 10 µL with DNase-free water. The reaction mixture was incubated at 37 °C for 5, 30, and 60 min and terminated by addition of 1 µL of 50 mM EDTA followed by incubation at 70 °C for 10 min. The experiment was repeated three times. The results of **31ON** and **31ON_C^{CA}G^{PA}U^{SA}A^{OP}** digestion were analyzed by 12.5% PAGE (to be comparable with previously reported[6]), and the results of **31ON_A^{NH2}U^{NMe}G^{NMe2}C^{NMe3}** and **31ON_A^{NH2}U^{EPh}G^{PA}C^{EAlk}** digestion were analyzed by 20% PAGE for a better band resolution.

Samples were visualized using fluorescence imaging (Figure S13). The values of DNA recoveries in each experiment and the average values are calculated in Table S13.

Table S13. Recovery (expressed in percent) of natural and hypermodified DNA after incubation with 0.01 U of DNase I for a set of three experiments.

DNA title	Experiment No.	Incubation time with DNase I		
		5 min	30 min	60 min
**31ON**	1	22%	8%	1%
	2	26%	4%	1%
	3	28%	7%	2%
	average	25±3%	6±2%	1%
**31ON_C^{CA}G^{PA}U^{SA}A^{OP}**	1	61%	25%	12%
	2	65%	21%	20%
	3	54%	19%	12%
	average	60±5%	22±2%	15±4%
**31ON_A^{NH2}U^{EPh}G^{PA}C^{EAlk}**	1	47%	18%	8%
	2	59%	10%	5%
	3	63%	10%	4%
	average	56±7%	13±4%	6±2%
**31ON_A^{NH2}U^{NMe}G^{NMe2}C^{NMe3}**	1	80%	39%	20%
	2	77%	46%	33%
	3	80%	39%	20%
	average	79±1%	41±3%	24±6%

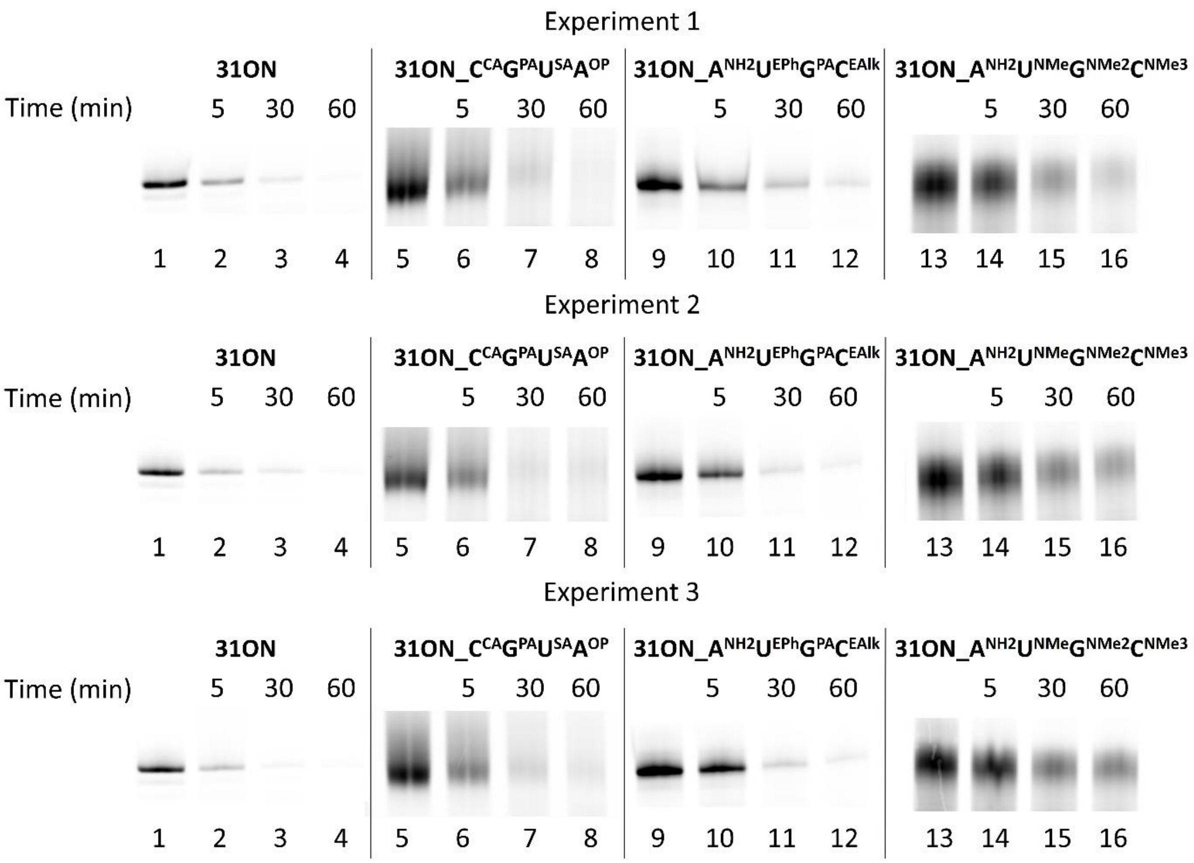

Figure S13. PAGE analysis of stability of natural **31ON** (lanes 2-4), anionic **31ON_C^{CA}G^{PA}U^{SA}A^{OP}** (lanes 6-8), mixed **31ON_A^{NH2}U^{EPh}G^{PA}C^{EAlk}** (lanes 10-12), and cationic **31ON_A^{NH2}U^{NMe}G^{NMe2}C^{NMe3}** (lanes 14-16) in the presence of 0.01 U of DNase I for 5 min (lanes 2, 6, 10, 14), 30 min (lanes 3, 7, 11, 15), and 60 min (lanes 4, 8, 12, 16). Lanes 1, 5, 9, and 13 were not incubated with DNase I. Data presented for a set of three experiments (from top to bottom).

2.14. ONs stability in human plasma

To study the ONs' stability in human plasma, four 5'-(6-FAM)-labelled 31-mer ONs (0.1 µg, 1 µL) – **31ON**, **31ON_C^{CA}G^{PA}U^{SA}A^{OP}**, **31ON_A^{NH2}U^{NMe}G^{NMe2}C^{NMe3}**, and **31ON_A^{NH2}U^{EPh}G^{PA}C^{EAlk}** (preparation described in section 2.13; for sequences see Table S2) were mixed with 19 µL of human plasma and incubated at 37 °C for 17 h. Then, plasma was deactivated by incubation at 70 °C for 20 min. To analyze the liquid part of the samples, reaction mixtures were centrifuged for 3 min (13000 rpm), followed by separation of the supernatant from the sediment. The experiment was repeated three times. Prior loading on gel, samples were

20 times diluted with water and mixed with PAGE stop solution. The results were analyzed by 20% PAGE and visualized using fluorescence imaging (Figure S14).

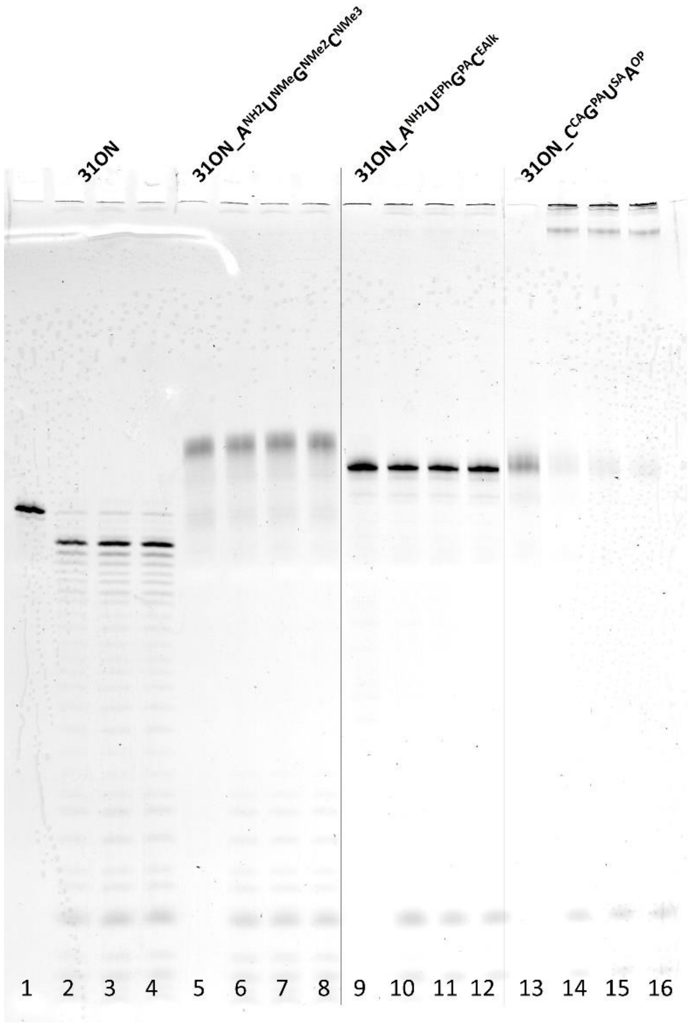

Figure S14. PAGE analysis of stability of natural **31ON** (lanes 2-4), cationic **31ON_A^{NH2}U^{NMe}G^{NMe2}C^{NMe3}** (lanes 6-8), mixed **31ON_A^{NH2}U^{EPh}G^{PA}C^{EAlk}** (lanes 10-12), and anionic **31ON_C^{CA}G^{PA}U^{SA}A^{OP}** (lanes 14-16) in human plasma for 17 hours. Lanes 1, 5, 9, and 13 were not incubated in human plasma.

2.15. Application of fully-modified ssONs for sequencing

2.15.1. Re-PCR of fully-modified ssONs obtained by PEX

To be able to sequence fully-modified ssONs after PEX, the following approach has been used (Scheme S3): modified **118DNA_A^{NH2}U^{NMe}G^{NMe2}C^{NMe3}** and **118DNA_A^{NH2}U^{EPh}G^{PA}C^{EAlk}** were

obtained by PEX using 45nt 5'-(6-FAM)-labelled extended primer Prim$^{Flank-NK98pr1}$-FAM and 98-mer template Temp^{NK98}-sC3 modified at 3'-end with three carbon spacer (sC3) preventing any unwanted extension during PEX (see further in section 2.15.2 and 2.15.3). Then the samples were loaded on 2% agarose gel (60 min, 125 V) followed by gel extraction of the product-containing area using a plastic pipette tip. Tips containing gel fragments were soaked overnight in water, and then the extracted modified template was used for re-PCR with primers Prim^{Flank}-FAM and Prim^{NK98pr2} and natural dNTPs (see section 2.15.4).

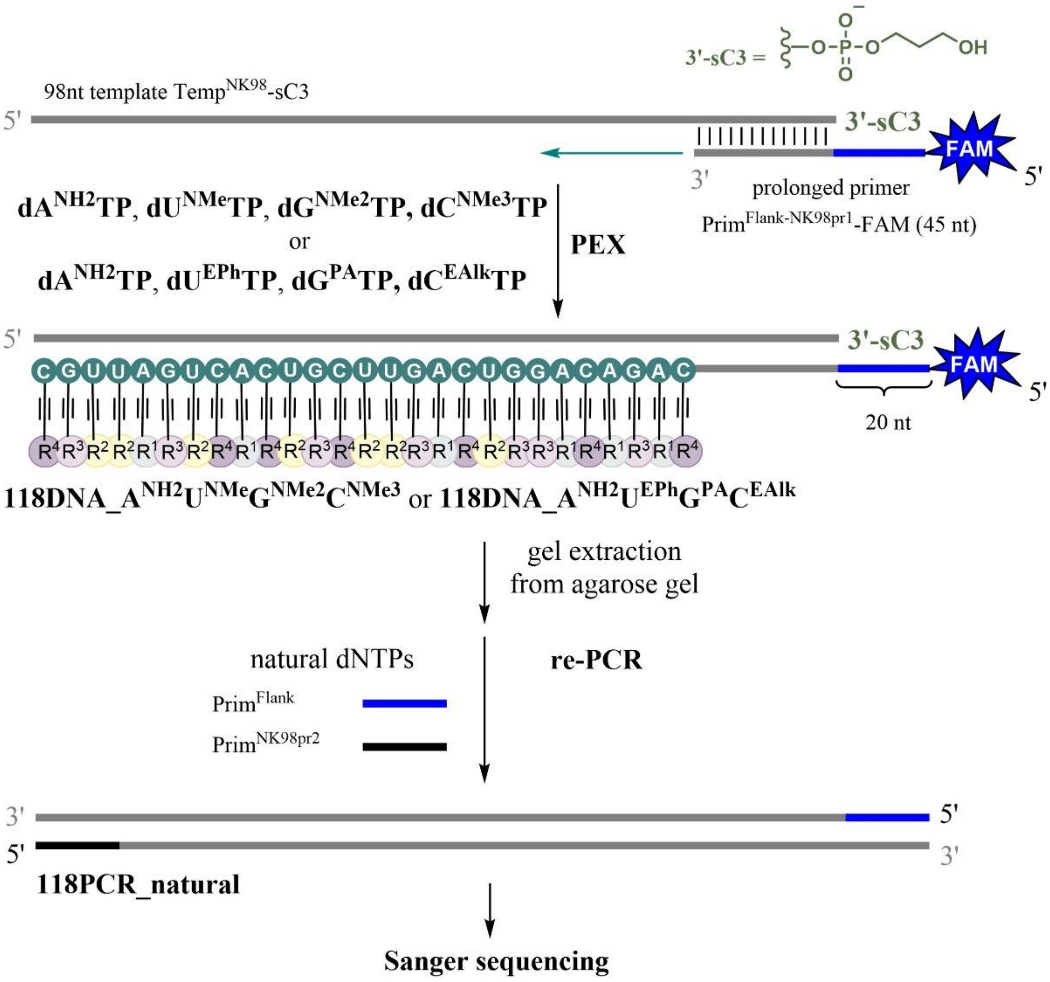

Scheme S3. PEX with an extended primer and a set of four modified **dN^{R}TP**s followed by re-PCR with natural dNTPs.

2.15.2. PEX – synthesis of 118DNA_A^{NH2}U^{NMe}G^{NMe2}C^{NMe3}

The reaction mixture (10 µL) containing template Temp^{NK98}-sC3 (3 µM, 0.75 µL), primer Prim$^{Flank-NK98pr1}$-FAM (3 µM, 0.5 µL), **dA^{NH2}TP** (0.25 mM, 1 µL), **dU^{NMe}TP** (2 mM, 1 µL),

dG^{NMe2}TP (2 mM, 1 µL), **dC^{NMe3}TP** (2 mM, 1 µL), $MgSO_4$ (25 mM, 0.6 µL), KOD XL DNA polymerase (2 U), and the enzyme reaction buffer (10X, 1 µL) as supplied by the manufacturer was incubated for 60 min at 72 °C. Positive control contained 0.35 U of KOD XL DNA polymerase and natural dNTPs (1 mM, 1 µL) and was incubated for 30 min at 72 °C. The reactions were stopped by cooling to 8 °C and samples were analyzed by 2% agarose gel electrophoresis (60 min, 125 V) and visualized using fluorescence imaging (Figure S15, lane 3).

2.15.3. PEX – synthesis of 118DNA_A^{NH2}U^{EPh}G^{PA}C^{EAlk}

The reaction mixture (10 µL) containing template Temp^{NK98}-sC3 (3 µM, 0.75 µL), primer Prim$^{Flank\text{-}NK98pr1}$-FAM (3 µM, 0.5 µL), **dA^{NH2}TP** (0.25 mM, 1 µL), **dU^{EPh}TP** (2 mM, 1 µL), **dG^{PA}TP** (2 mM, 1 µL), **dC^{EAlk}TP** (2 mM, 1 µL), KOD XL DNA polymerase (0.2 U), and the enzyme reaction buffer (10X, 1 µL) as supplied by the manufacturer was incubated for 30 min at 60 °C. The reactions were stopped by cooling to 8 °C and samples were analyzed by 2% agarose gel electrophoresis (60 min, 125 V) and visualized using fluorescence imaging (Figure S15, lane 4).

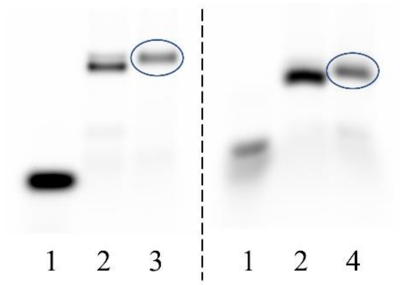

Figure S15. Agarose gel analysis of PEX reactions using extended primer Prim$^{Flank\text{-}NK98pr1}$-FAM and a set of four modified **dN^{R}TP**s: (lane 1) primer Prim$^{Flank\text{-}NK98pr1}$-FAM; (lane 2) positive control; (lane 3) **dA^{NH2}TP**, **dU^{NMe}TP**, **dG^{NMe2}TP**, **dC^{NMe3}TP**; (lane 4) **dA^{NH2}TP**, **dU^{EPh}TP**, **dG^{PA}TP**, **dC^{EAlk}TP**. Modified DNA template was extracted from the circled areas of the gel.

2.15.4. Re-PCR – synthesis of 118PCR_natural (for further sequencing)

Method J. The reaction mixtures (20 µL) contained traces of **118DNA_A^{NH2}U^{NMe}G^{NMe2}C^{NMe3}** as a template (synthesis described in section 2.15.2), forward primer Prim^{Flank}-FAM and reverse primer Prim^{NK98pr2}-Cy5 (10 µM, 2 µL each), all four natural

dNTPs (1 mM, 4.5 μL), Pwo DNA polymerase (0.3 U), and the enzyme reaction buffer (10X, 2 μL) as supplied by the manufacturer. The positive control contained 0.2 U of Pwo DNA polymerase, template Temp^{NK98} (0.5 μM, 0.5 μL), primers Prim$^{Flank\text{-}NK98pr1}$-FAM and Prim^{NK98pr2}-Cy5 (10 μM, 2 μL each), and natural dNTPs (1 mM, 4.5 μL). The reaction mixture was under cycling protocol: 94 °C for 5 min, followed by 30 cycles at 94 °C for 1 min, 61 °C for 30 s, and 72 °C for 1 min, followed by a final elongation step at 72 °C for 5 min. The obtained **118PCR_natural_1** DNA duplex was analyzed by 12.5% PAGE and visualized using fluorescence imaging (Figure S16, lane 3).

Method K. The reaction mixtures (20 μL) contained traces of **118DNA_A^{NH2}U^{EPh}G^{PA}C^{EAlk}** as a template (synthesis described in section 2.15.3), forward primer Prim^{Flank}-FAM and reverse primer Prim^{NK98pr2}-Cy5 (10 μM, 2 μL each), all four natural dNTPs (1 mM, 4.5 μL), $MgSO_4$ (25 mM, 1.2 μL), Pwo DNA polymerase (0.3 U), and the enzyme reaction buffer (10X, 2 μL) as supplied by the manufacturer. The positive control was carried out as described in Method J. The reaction mixture was under cycling protocol: 94 °C for 5 min, followed by 30 cycles at 94 °C for 1 min, 54 °C for 30 s, and 72 °C for 1 min, followed by a final elongation step at 72 °C for 5 min. The obtained **118PCR_natural_2** DNA duplex was analyzed by 12.5% PAGE and visualized using fluorescence imaging (Figure S16, lane 4).

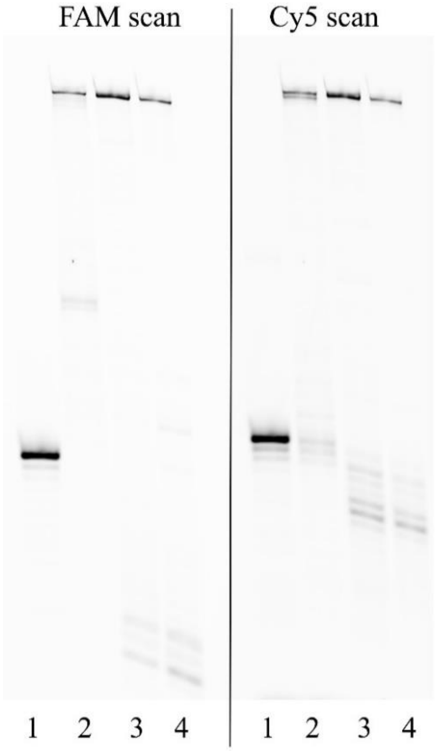

Figure S16. Denaturing PAGE analysis of the performed re-PCRs: (lane 1) primers Prim^{Flank}-FAM and Prim^{NK98pr2}-Cy5; (lane 2) positive control; (lane 3) **118PCR_natural_1**; (lane 4) **118PCR_natural_2**.

2.16. Sanger sequencing

In order to have dsDNA for Sanger sequencing, **118PCR_natural_1** and **118PCR_natural_2** were obtained using either **118DNA_A^{NH2}U^{NMe}G^{NMe2}C^{NMe3}** or **118DNA_A^{NH2}U^{EPh}G^{PA}C^{EAlk}** as templates by the procedure described in section 2.15 (re-PCRs were performed with non-labelled primers). Samples were purified using QIAquick PCR Purification Kit according to the manufacturer's protocol. Resulting natural 118bp DNA samples (40 ng) were sent for Sanger sequencing using corresponding primers (5 µL, 5 µM).

2.17. Results of Sanger sequencing

2.17.1. 118PCR_natural_1

A) Sequencing chromatogram with forward primer Prim^{Flank}

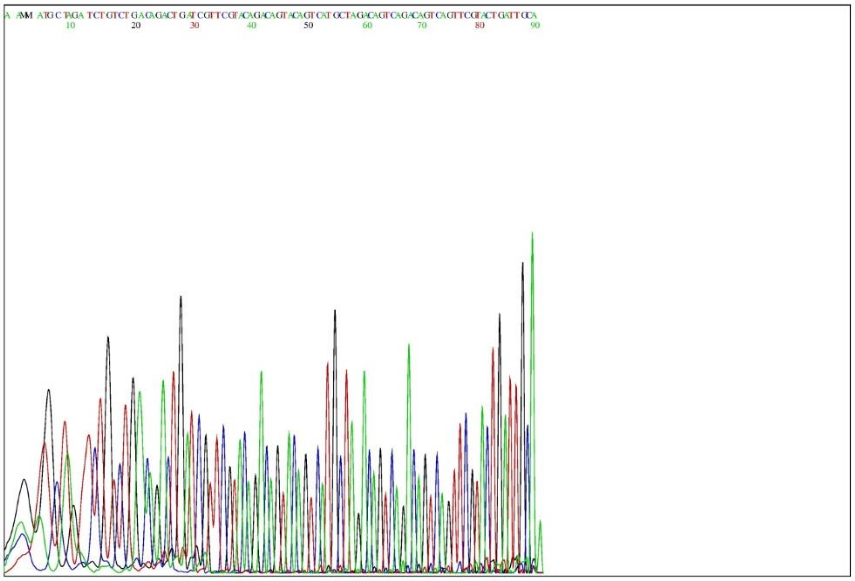

B) Sequencing chromatogram with reverse primer Prim^{NK98pr2}

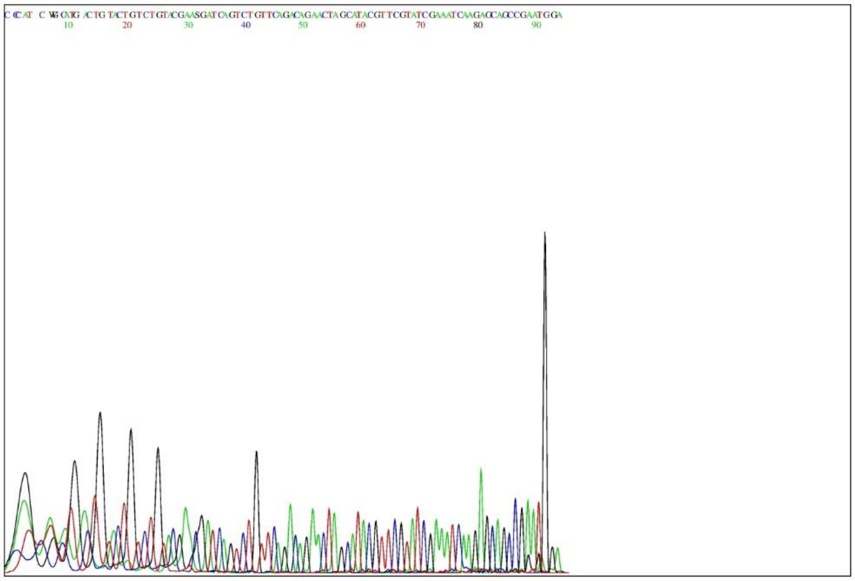

C) Region of properly analyzed sequence shown in blue box

CATTCGGCTGCTCTTGATTTCGATACGAACGTATGCTAGTTCTGTCTGAACAGACTGATCGTTCGTACAGACAGTACAGTCATGCTAGACAGTCAGACAGTCAGTTCGTACTGATTGC

2.17.2. 118PCR_natural_2

A) Sequencing chromatogram with forward primer Prim^{Flank}

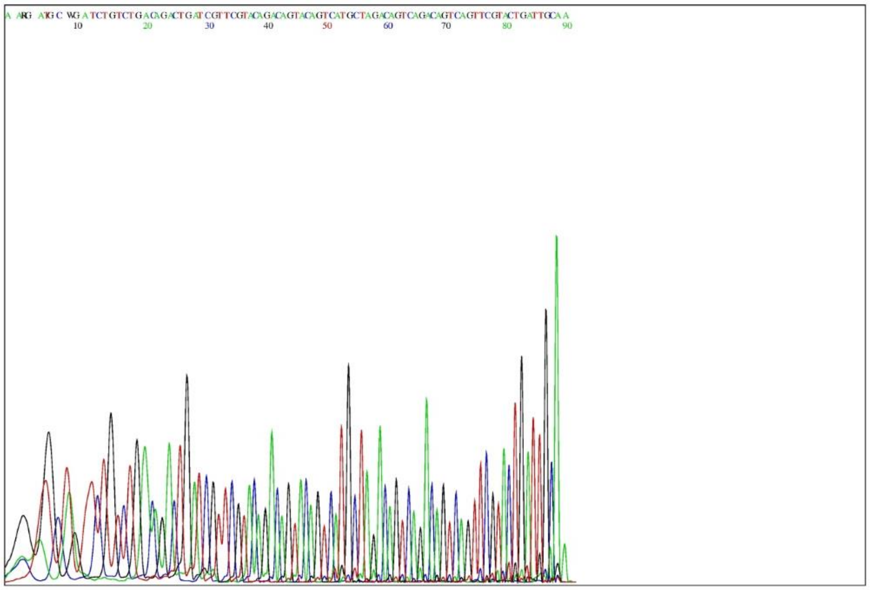

B) Sequencing chromatogram with reverse primer Prim^{NK98pr2}

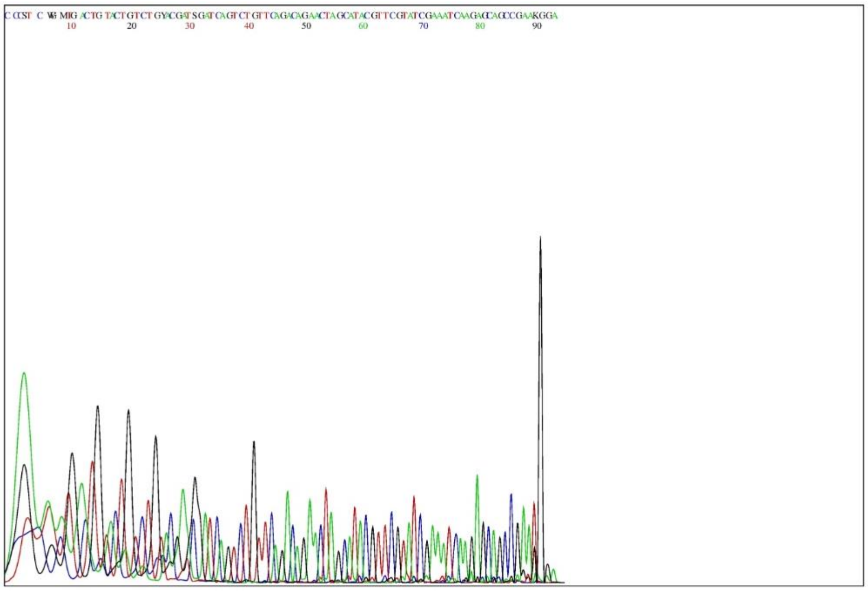

C) Region of properly analyzed sequence shown in blue box

CATTCGGCTGCTCTTGATTTCGATACGAACGTATGCTAGTTCTGTCTGAACAGACTGATCGTTCGTACAGACAGTACAGTCATGCTAGACAGTCAGACAGTCAGTTCGTACTGATTGC

3. Experimental section – CD spectroscopy and melting temperatures determination

General remarks

The circular dichroism (CD) measurements were performed on a Jasco-1500 spectropolarimeter (JASCO Inc. Easton, MD, USA). in spectral range from 200 nm to 400 nm in 1 mm rectangular quartz cell with following experimental setup: standard instrument sensitivity, 1 nm bandwidth, a scanning speed of 10 nm/min, a response time of 8 s and three accumulations. After baseline subtraction, the final data were recalculated on the concentration of nucleotide and expressed as molar differential extinction ΔƐ ($cm^{-1}mol^{-1}$).

Melting temperature measurements performed using UV-absorption were carried out on Cary 100 Bio UV/VIS Spectrophotometer with temperature controller (Varian). The spectra were recorded in 1 cm rectangular quartz cell, in temperature range 25 °C - 95 °C with temperature increment 1 °C/min under 260 nm detection and were obtained from three cycles (6 ramps in total). Tm values (in °C) were calculated using first negative derivative of intensity over temperature.

3.1. Preparation of the samples

3.1.1. Preparation of 98DNA

The reaction mixture (20 µL) contained template $Temp^{NK98}$ (0.5 µM, 0.5 µL), reverse primer $Prim^{NK98pr1}$ and forward primer $Prim^{NK98pr2}$ (10 µM, 6 µL each), natural dNTPs (2 mM, 2 µL), KOD XL DNA polymerase (0.6 U) and a corresponding reaction buffer (10X, 2 µL) as supplied by the manufacturer. The reaction mixture was under cycling protocol: 94 °C for 5 min, followed by 30 cycles at 94 °C for 1 min, 53 °C for 30 sec, and 72 °C for 1 min, followed by a final elongation step at 72 °C for 5 min. The reaction was repeated 8 times to obtain sufficient DNA concentration for measurements. Joined fractions of **98DNA** were purified using QIAquick PCR Purification Kit (Qiagen), concentrated, diluted to 200 µL final volume with Tris-HCl buffer (10 mM, 1 mM EDTA, 65 mM NaCl, pH 7.5), and set for measurements.

3.1.2. Preparation of 98DNA_$A^{NH2}U^{NMe}G^{NMe2}C^{NMe3}$

The reaction mixture (50 µL) containing template $Temp^{NK98}$ (100 µM, 0.75 µL), primer $Prim^{NK98pr1}$ (100 µM, 0.75 µL), set of four modified **$dN^{R}TP$s** (R = NH2, NMe, NMe2, NMe3; N = A, U, G, C) (4 mM, 2.5 µL; only in the case of **$dC^{NMe3}TP$** 6 mM), $MgSO_4$ (25 mM, 3 µL), betaine (5 M, 15 µL), KOD XL DNA polymerase (2.5 U), and the enzyme reaction buffer (10X,

5 μL) as supplied by the manufacturer was incubated for 2 h at 72 °C. The reaction was stopped by cooling to 8 °C. The reaction was repeated 17 times to obtain sufficient DNA concentration for measurements. Joined fractions of **98DNA_A^{NH2}U^{NMe}G^{NMe2}C^{NMe3}** were purified using Agencourt AMPure XP magnetic particles, concentrated, diluted to 200 μL final volume with Tris-HCl buffer (10 mM, 1 mM EDTA, 65 mM NaCl, pH 7.5), and set for measurements.

3.1.3. Preparation of 98DNA_A^{NH2}U^{EPh}G^{PA}C^{EAlk}

The reaction mixture (50 μL) containing template Temp^{NK98} (100 μM, 3 μL), primer Prim^{NK98pr1} (100 μM, 3 μL), set of four modified **dN^{R}TP**s (R = NH2, EPh, PA, EAlk; N = A, U, G, C) (4 mM, 2.5 μL), KOD XL DNA polymerase (2.5 U), and the enzyme reaction buffer (10X, 5 μL) as supplied by the manufacturer was incubated for 1 h at 60 °C. The reaction was stopped by cooling to 8 °C. The reaction was repeated 3 times to obtain sufficient DNA concentration for measurements. Joined fractions of **98DNA_A^{NH2}U^{EPh}G^{PA}C^{EAlk}** were purified using Agencourt AMPure XP magnetic particles, concentrated, diluted to 200 μL final volume with Tris-HCl buffer (10 mM, 1 mM EDTA, 65 mM NaCl, pH 7.5), and set for measurements.

3.1.4. Preparation of 98DNA_dsA^{NH2}U^{EPh}G^{PA}C^{EAlk}

Non-labelled oligonucleotides **98ON_A^{NH2}U^{EPh}G^{PA}C^{EAlk}** and **98cON_A^{NH2}U^{EPh}G^{PA}C^{EAlk}** (preparation described below) were annealed together in Tris-HCl buffer (10 mM, 1 mM EDTA, 65 mM NaCl, pH 7.5) under following protocol: 95 °C for 5 min, followed by gradual cooling to 25 °C for 90 min. To prove the suitability of the conditions, annealing was additionally performed with 5’-(6-FAM)-labelled **98ON_A^{NH2}U^{EPh}G^{PA}C^{EAlk}** and 5’-Cy5-labelled **98cON_A^{NH2}U^{EPh}G^{PA}C^{EAlk}**. The labelled double-stranded product was further analyzed by 2% agarose gel electrophoresis and visualized using fluorescence imaging (Figure S17). The non-labelled sample was diluted to 200 μL final volume with the annealing buffer and set for measurements.

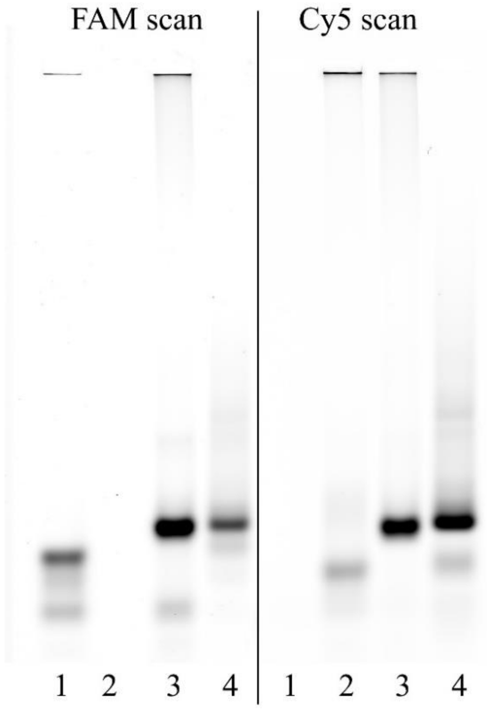

Figure S17. Agarose gel displaying (1) 5'-(6-FAM)-labelled single-stranded **98ON_A^{NH2}U^{EPh}G^{PA}C^{EAlk}**, (2) 5'-Cy5-labelled single-stranded **98cON_A^{NH2}U^{EPh}G^{PA}C^{EAlk}**, (3) annealed double-stranded **98DNA_dsA^{NH2}U^{EPh}G^{PA}C^{EAlk}**; (4) double-stranded **98PCR_A^{NH2}U^{EPh}G^{PA}C^{EAlk}** (described in section 2.11, Method I, but purified using Agencourt AMPure XP magnetic particles).

Preparation of **98ON_A^{NH2}U^{EPh}G^{PA}C^{EAlk}** and **98cON_A^{NH2}U^{EPh}G^{PA}C^{EAlk}**:

To obtain double-stranded products **98DNA_A^{NH2}U^{EPh}G^{PA}C^{EAlk}** and **98cDNA_A^{NH2}U^{EPh}G^{PA}C^{EAlk}**, PEX reaction, described in section 3.1.3, was repeated 8 times using dual-biotinylated templates (template Temp^{NK98}-bio and primer Prim^{NK98pr1} in the case of **98DNA_A^{NH2}U^{EPh}G^{PA}C^{EAlk}** and template Temp^{NK98_comp}-bio and primer Prim^{NK98pr2} in the case of **98cDNA_A^{NH2}U^{EPh}G^{PA}C^{EAlk}**). After that, the products were purified using Agencourt AMPure XP magnetic particles to remove shorter side-product ONs, and then modified strands were separated from the templates using Streptavidin magnetic particles (Roche) according to DBStv magnetoseparation procedure (section 2.7).

3.1.5. Preparation of 98DNA_ds$A^{NH2}U^{NMe}G^{NMe2}C^{NMe3}$ (unsuccessful)

The mixture of 5'-(6-FAM)-labelled **98ON_$A^{NH2}U^{NMe}G^{NMe2}C^{NMe3}$** and 5'-Cy5-labelled **98cON_$A^{NH2}U^{NMe}G^{NMe2}C^{NMe3}$** (preparation described below) in Tris-HCl buffer (10 mM, 1 mM EDTA, 65 mM NaCl, pH 7.5) was under the following protocol: 95 °C for 5 min, followed by gradual cooling to 25 °C for 90 min. The results were further analyzed by 2% agarose gel electrophoresis and visualized using fluorescence imaging (Figure S18).

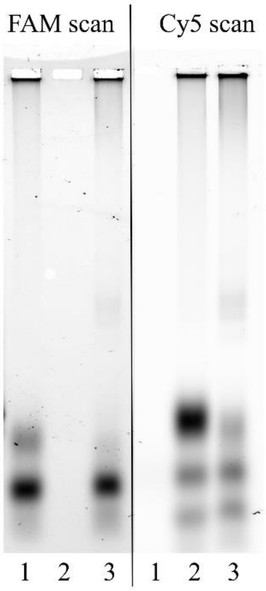

Figure S18. Agarose gel displaying: A) (lane 1) 5'-(6-FAM)-labelled single-stranded **98ON_$A^{NH2}U^{NMe}G^{NMe2}C^{NMe3}$**, (lane 2) 5'-Cy5-labelled single-stranded **98cON_$A^{NH2}U^{NMe}G^{NMe2}C^{NMe3}$**, (lane 3) result of **98ON_$A^{NH2}U^{NMe}G^{NMe2}C^{NMe3}$** and **98cON_$A^{NH2}U^{NMe}G^{NMe2}C^{NMe3}$** annealing.

Preparation of **98ON_$A^{NH2}U^{NMe}G^{NMe2}C^{NMe3}$** and **98cON_$A^{NH2}U^{NMe}G^{NMe2}C^{NMe3}$**:

To obtain double-stranded products **98DNA_$A^{NH2}U^{NMe}G^{NMe2}C^{NMe3}$** and **98cDNA_$A^{NH2}U^{NMe}G^{NMe2}C^{NMe3}$**, PEX reaction, described in section 3.1.2, was performed using dual-biotinylated templates (template $Temp^{NK98}$-bio and primer $Prim^{NK98pr1}$-FAM in the case of **98DNA_$A^{NH2}U^{NMe}G^{NMe2}C^{NMe3}$** and template $Temp^{NK98\_comp}$-bio and primer $Prim^{NK98pr2}$-Cy5 in the case of **98cDNA_$A^{NH2}U^{NMe}G^{NMe2}C^{NMe3}$**). After that, the products were purified using Agencourt AMPure XP magnetic particles to remove shorter side-product ONs, and then modified

strands were separated from the templates using Streptavidin magnetic particles (Roche) according to DBStv magnetoseparation procedure (section 2.7).

3.2. UV-vis spectroscopy

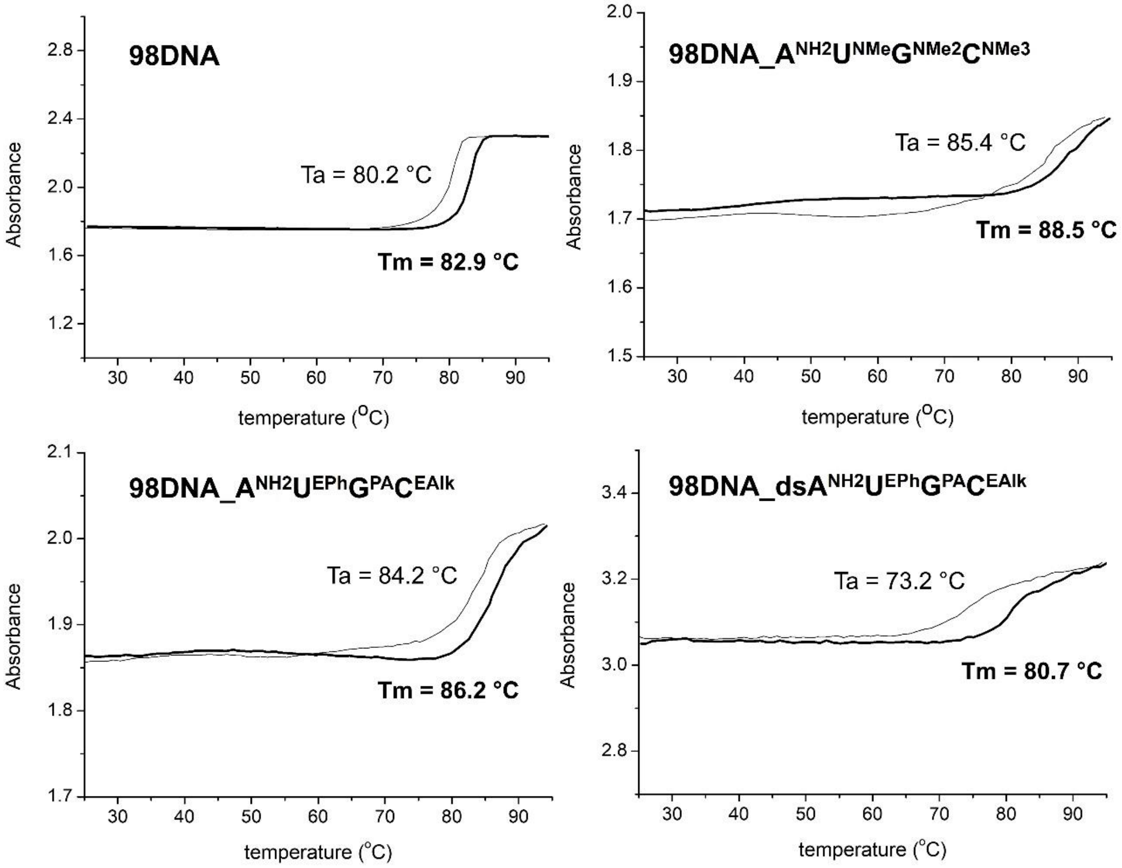

Figure S19. Melting curves with calculated melting (Tm) and annealing (Ta) temperatures of **98DNA**, **98DNA_A^{NH2}U^{NMe}G^{NMe2}C^{NMe3}**, **98DNA_A^{NH2}U^{EPh}G^{PA}C^{EAlk}**, **98DNA_dsA^{NH2}U^{EPh}G^{PA}C^{EAlk}** obtained from UV spectroscopy at 260 nm absorption (buffer composition: 10 mM Tris, 65 mM NaCl, 1 mM EDTA, pH 7.5).

4. Experimental section – microscopy

General remarks

The confocal microscopy imaging measurements were performed on a confocal microscope ZEISS LSM 980. Water immersion objective C-Apochromat 40x/1.20W was used. The samples were irradiated with a laser beam being set at 488 nm, the detector (GaAsP – PMT) range was set

to 490 – 685 nm. Detector gain 845 V and detector offset 5 were set. The images were processed using ZEISS ZEN 3.2 software.

4.1. Preparation of double-stranded samples

Double-stranded **98DNA**, **98DNA_A^{NH2}U^{NMe}G^{NMe2}C^{NMe3}**, and **98DNA_A^{NH2}U^{EPh}G^{PA}C^{EAlk}** were prepared according to procedures described in sections 3.1.1, 3.1.2, 3.1.3, using 5'-(6-FAM)-labelled primer Prim^{NK98pr1}-FAM. The products were purified using mini Quick Spin Oligo Columns (Roche), concentrated, and diluted to 0.1 µM solutions with Milli-Q water.

Preparation of 98DNA_C^{CA}G^{PA}U^{SA}A^{OP}

The reaction mixture (10 µL) containing template Temp^{NK98} (100 µM, 0.5 µL), primer Prim^{NK98pr1}-FAM (100 µM, 0.5 µL), set of four modified **dN^{R}TP**s (R = OP, SA, PA, CA; N = A, U, G, C; structures specified in section 1.2) (4 mM, 0.75 µL), KOD XL DNA polymerase (1.25 U), and the enzyme reaction buffer (10X, 1 µL) as supplied by the manufacturer was incubated for 40 min at 60 °C. The reaction was stopped by cooling to 8 °C. The product was purified using mini Quick Spin Oligo Columns (Roche), concentrated, and diluted to 0.1 µM solution with Milli-Q water.

4.2. Preparation of single-stranded samples

Single-stranded **98ON**, **98ON_A^{NH2}U^{NMe}G^{NMe2}C^{NMe3}**, **98ON_A^{NH2}U^{EPh}G^{PA}C^{EAlk}**, and **98ON_C^{CA}G^{PA}U^{SA}A^{OP}** (for sequences see Table S2) were prepared from corresponding double-stranded PEX products (preparation described in section 4.1), obtained with dual-biotinylated templates, using Streptavidin magnetic particles (Roche) according to DBStv magnetoseparation procedure (section 2.7). Further, single-stranded products were concentrated and diluted to 0.1 µM solutions with Milli-Q water.

4.3. Confocal microscopy imaging

Drops of solutions (0.1 µM) of single-stranded **98ON**, **98ON_A^{NH2}U^{NMe}G^{NMe2}C^{NMe3}**, **98ON_A^{NH2}U^{EPh}G^{PA}C^{EAlk}**, **98ON_C^{CA}G^{PA}U^{SA}A^{OP}** and double-stranded **98DNA**, **98DNA_A^{NH2}U^{NMe}G^{NMe2}C^{NMe3}**, **98DNA_A^{NH2}U^{EPh}G^{PA}C^{EAlk}**, and **98DNA_C^{CA}G^{PA}U^{SA}A^{OP}** in water were casted onto glass of a single-chamber glass bottom dish (In Vitro Scientific). Then, the

cover lid was placed onto the dish and the samples were immediately mounted onto a stage of a confocal microscope (ZEISS LSM 980). The results are shown in Figure S20.

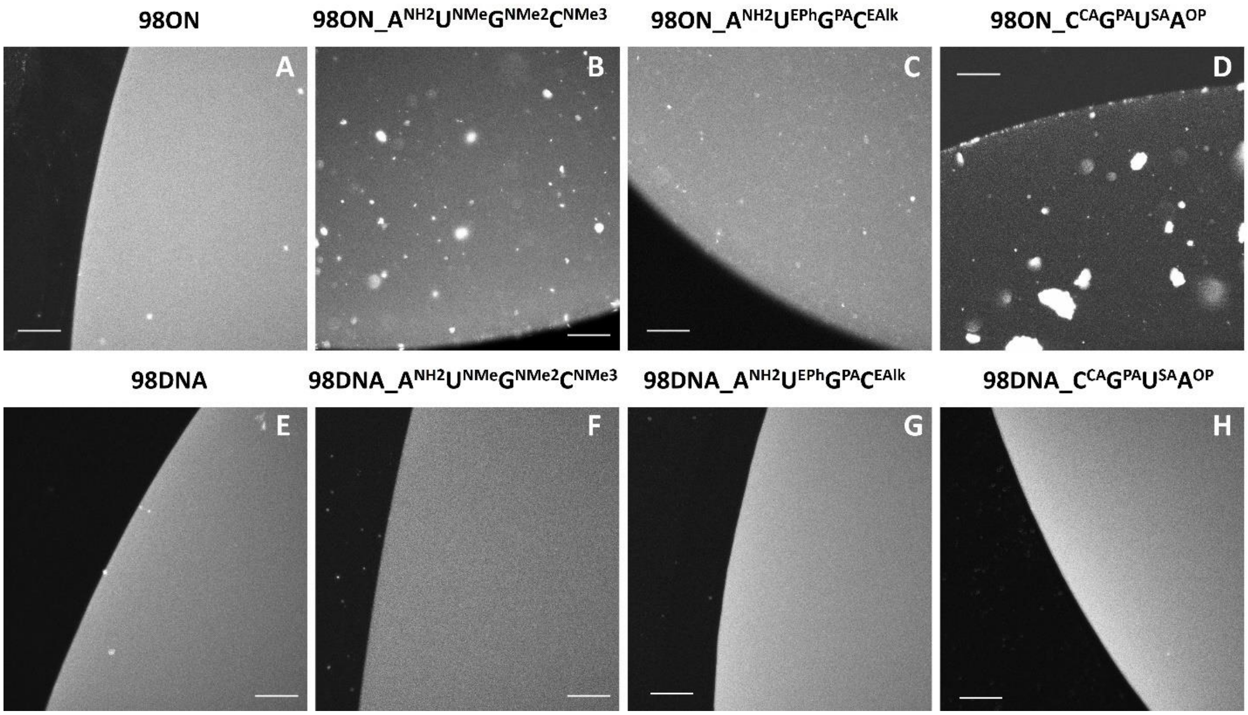

Figure S20. Confocal microscopy images of water solutions of single-stranded samples: (A) **98ON**, (B) **98ON_A^{NH2}U^{NMe}G^{NMe2}C^{NMe3}**, (C) **98ON_A^{NH2}U^{EPh}G^{PA}C^{EAlk}**, (D) **98ON_C^{CA}G^{PA}U^{SA}A^{OP}** and double-stranded samples (E) **98DNA**, (F) **98DNA_A^{NH2}U^{NMe}G^{NMe2}C^{NMe3}**, (G) **98DNA_A^{NH2}U^{EPh}G^{PA}C^{EAlk}**, (H) **98DNA_C^{CA}G^{PA}U^{SA}A^{OP}**. Scale bar is 50 µm.

5. Copies of ESI spectra of modified oligonucleotides

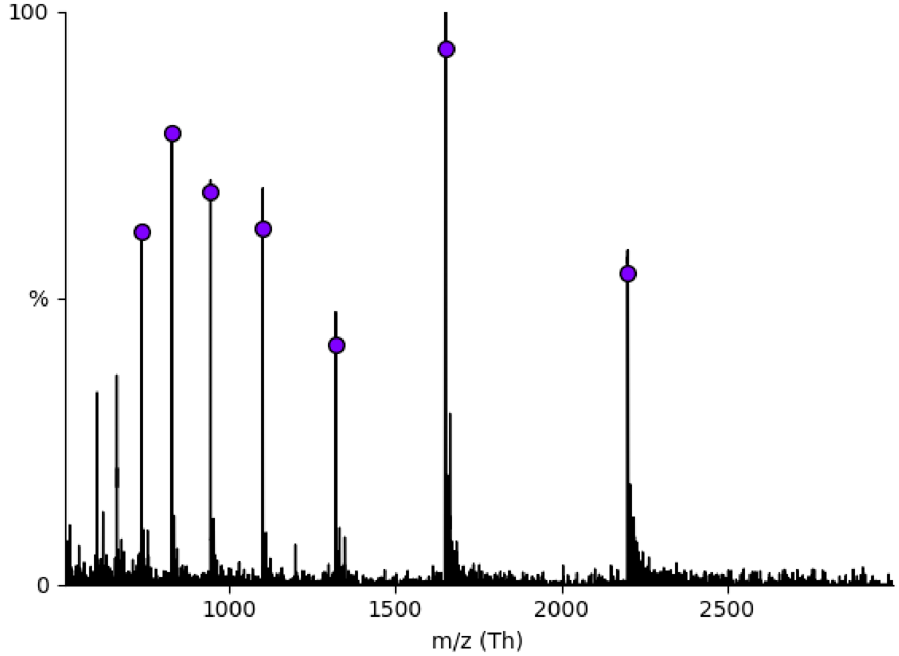

Figure S21. Raw MS spectrum of **19ON_A^{NH2}**

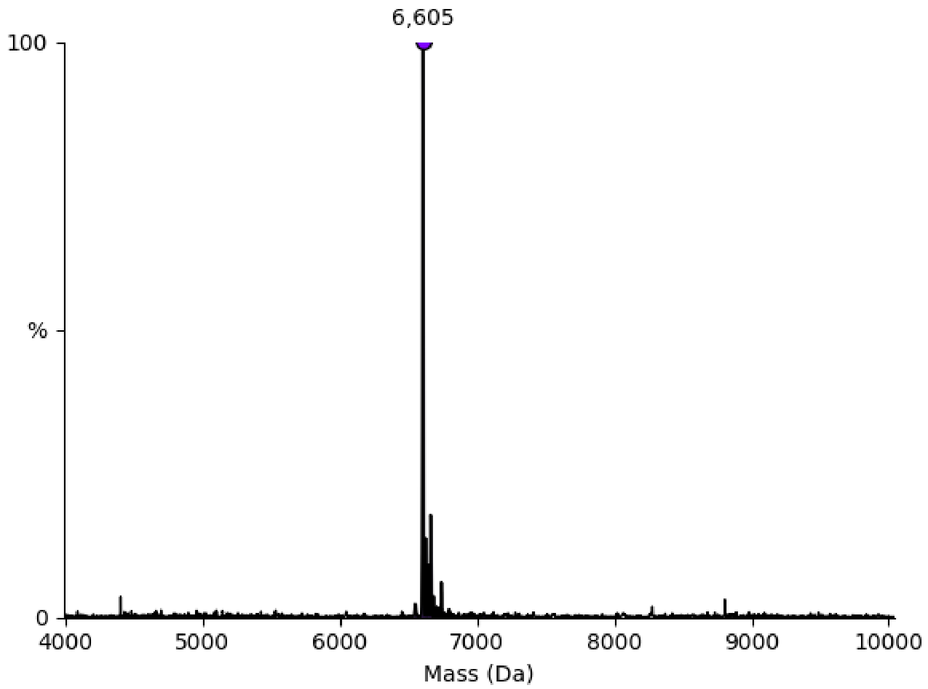

Figure S22. Deconvoluted MS spectrum of **19ON_A^{NH2}**: calculated: 6605.9 Da; found: 6605 Da, Δ = 0.9 Da

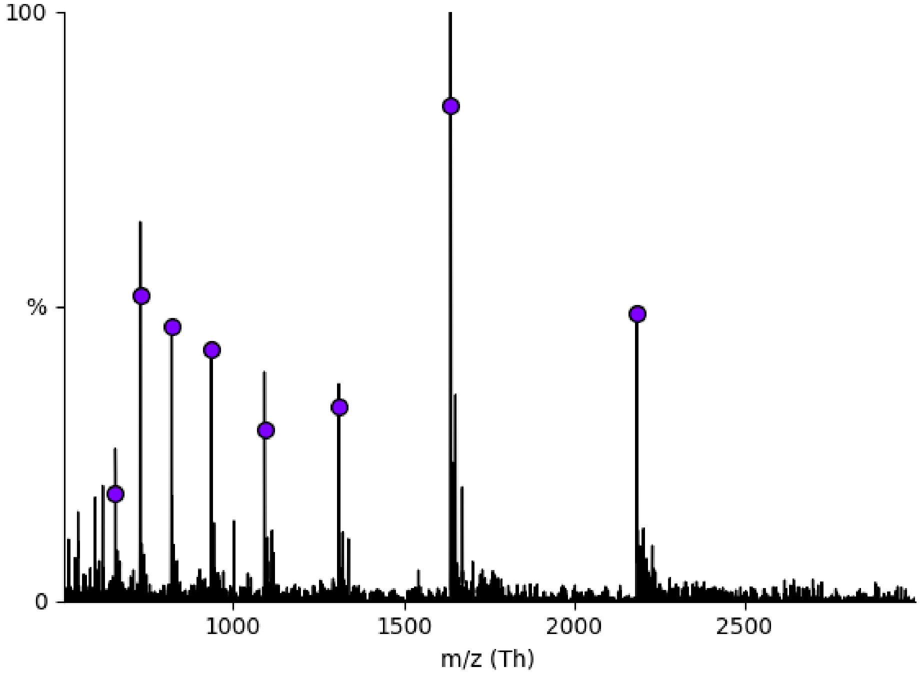

Figure S23. Raw MS spectrum of **19ON_U^{NMe}**

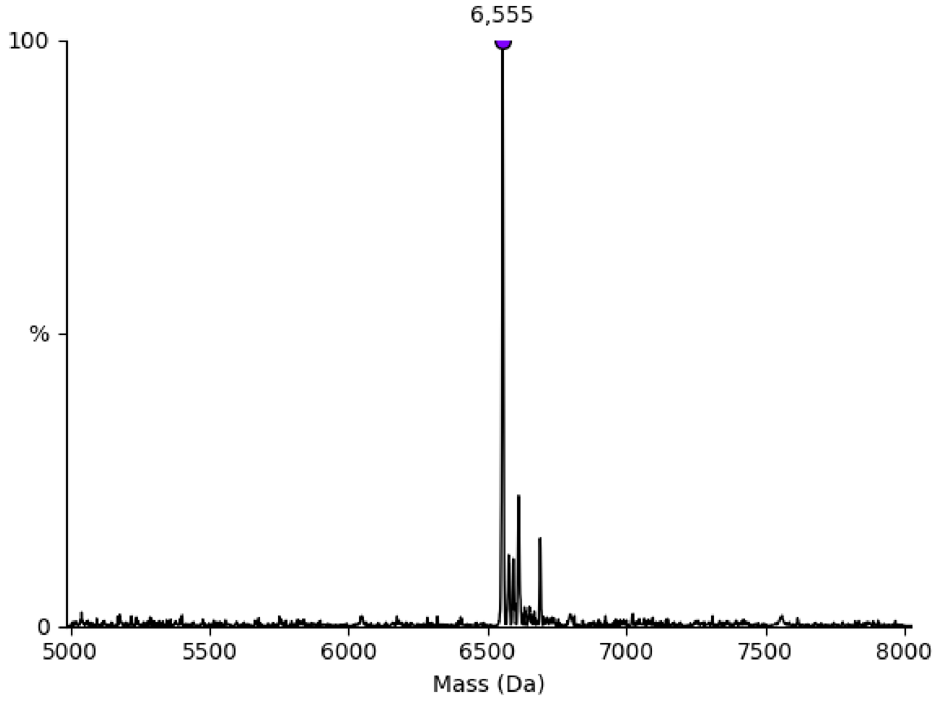

Figure S24. Deconvoluted MS spectrum of **19ON_U^{NMe}**: calculated: 6555.9 Da; found: 6555 Da, Δ = 0.9 Da

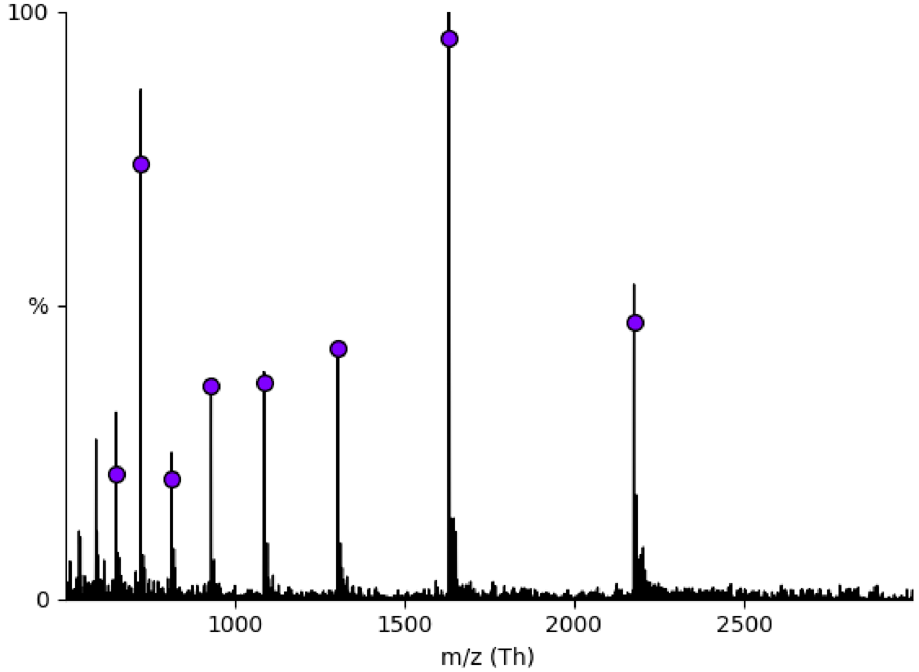

Figure S25. Raw MS spectrum of **19ON_G^{NMe2}**

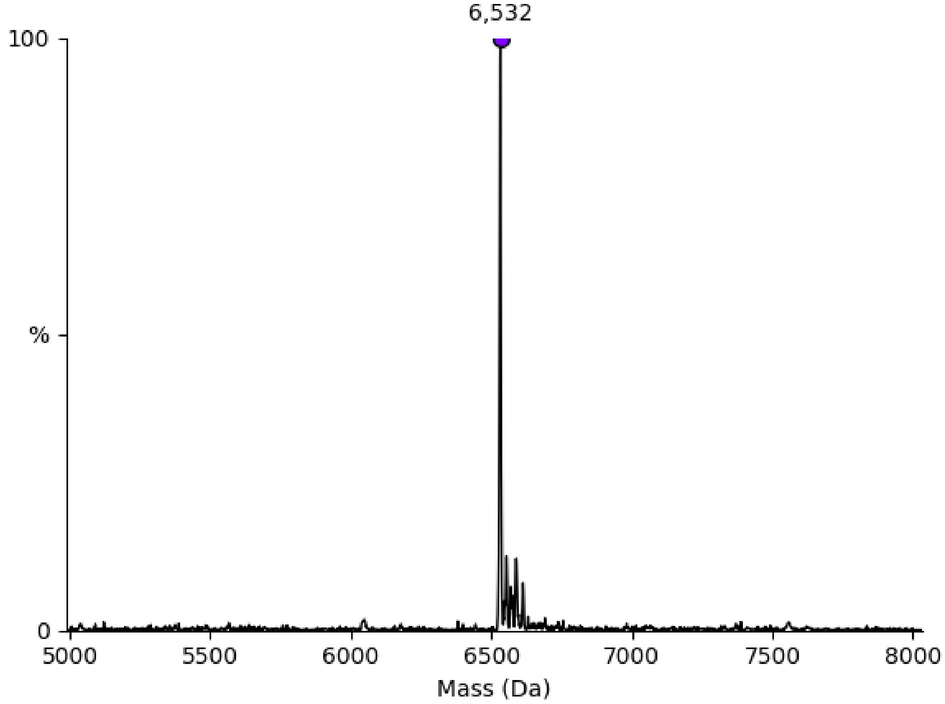

Figure S26. Deconvoluted MS spectrum of **19ON_G^{NMe2}**: calculated: 6532.9 Da; found: 6532 Da, Δ = 0.9 Da

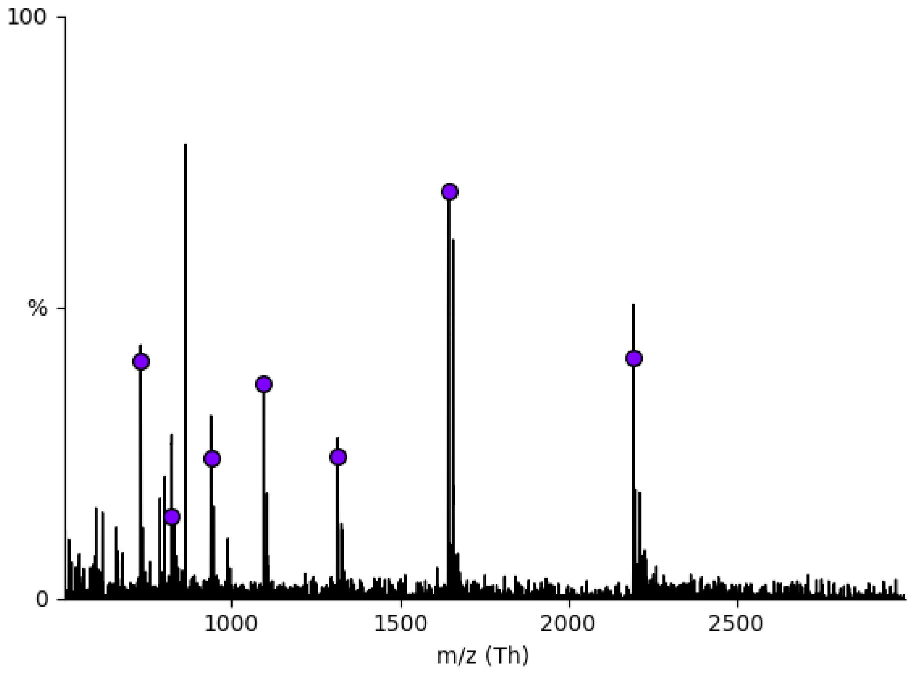

Figure S27. Raw MS spectrum of **19ON_C^{NMe3}**

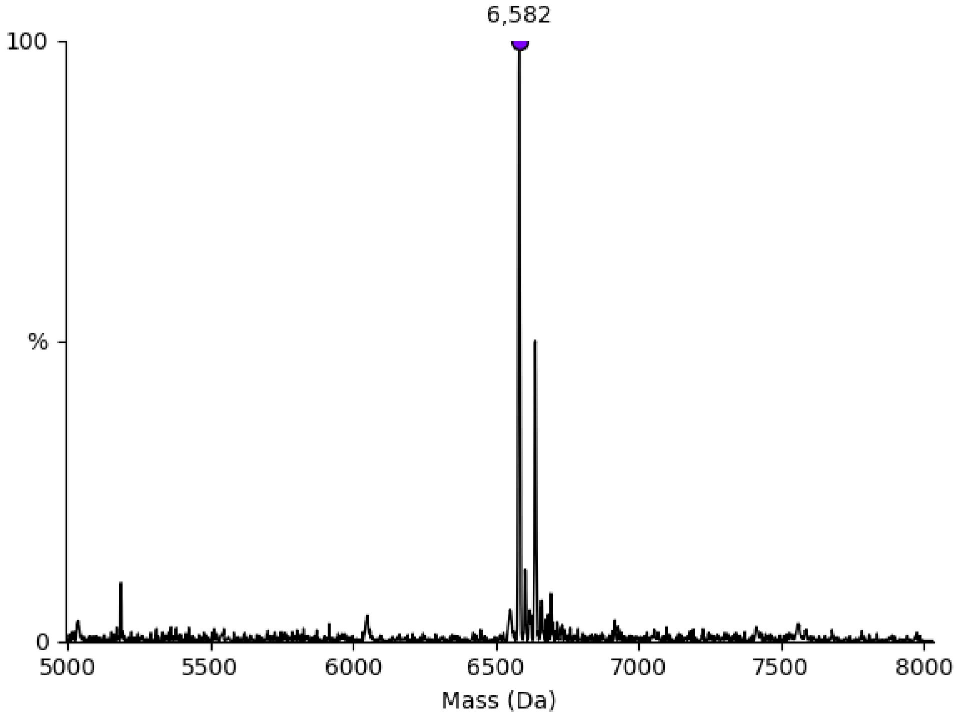

Figure S28. Deconvoluted MS spectrum of **19ON_C^{NMe3}**: calculated: 6583.9 Da; found: 6582 Da, Δ = 1.9 Da

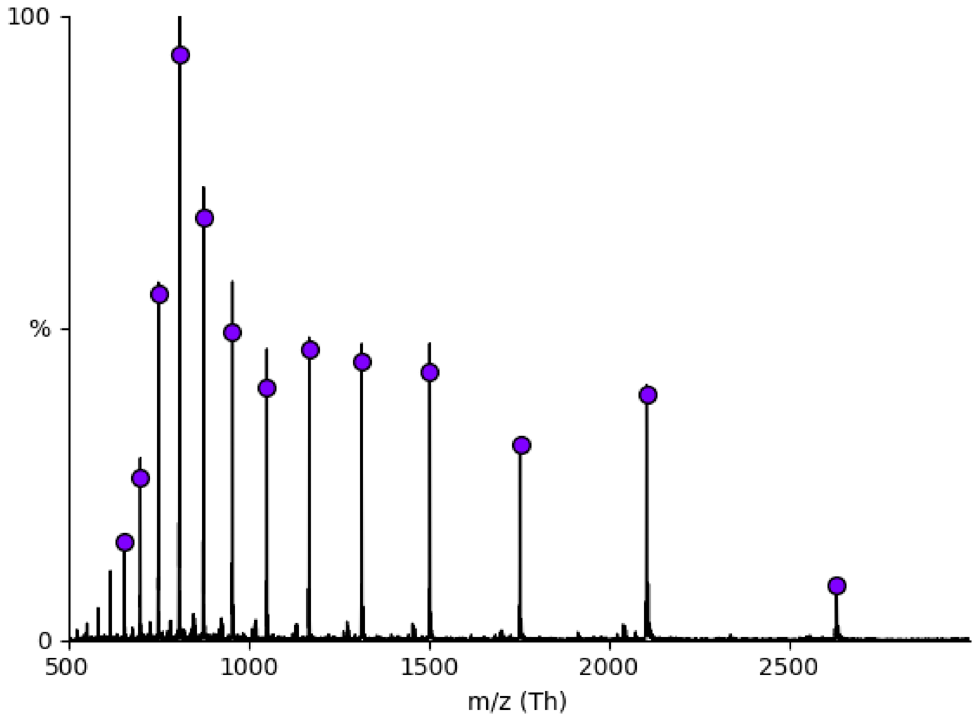

Figure S29. Raw MS spectrum of **31ON_A^{NH2}**

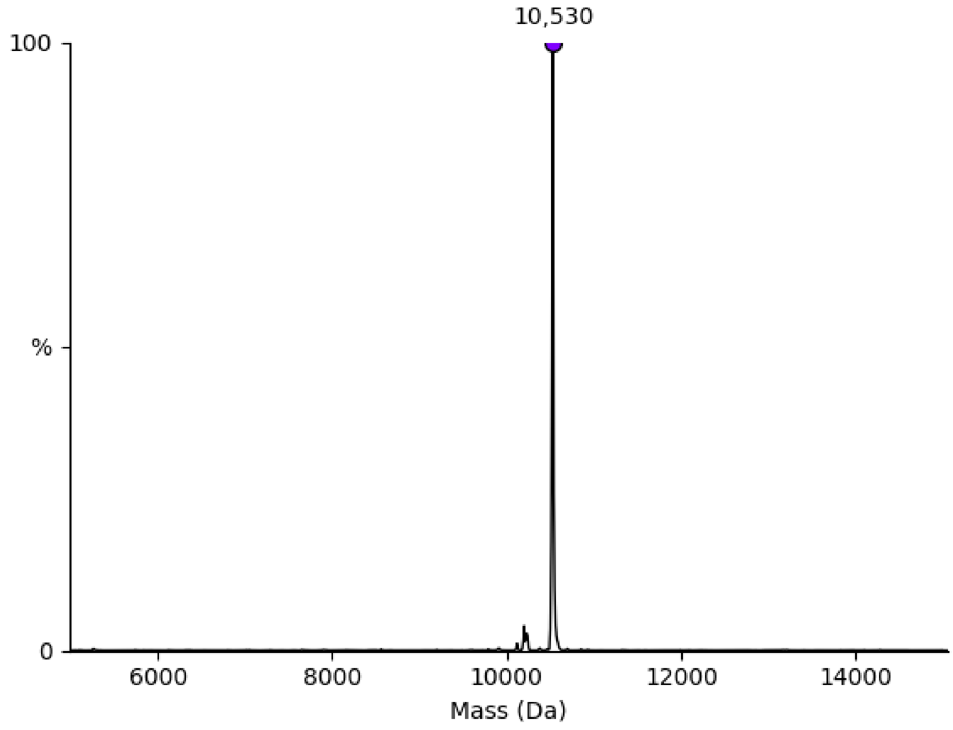

Figure S30. Deconvoluted MS spectrum of **31ON_A^{NH2}**: calculated: 10530.3 Da; found: 10530 Da, Δ = 0.3 Da

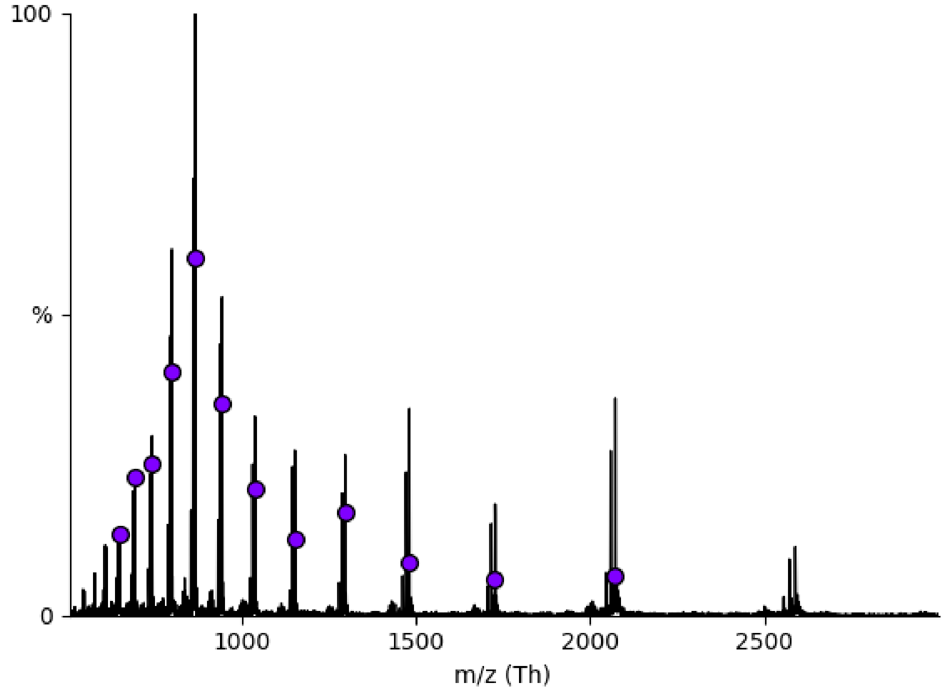

Figure S31. Raw MS spectrum of **31ON_U^{NMe}**

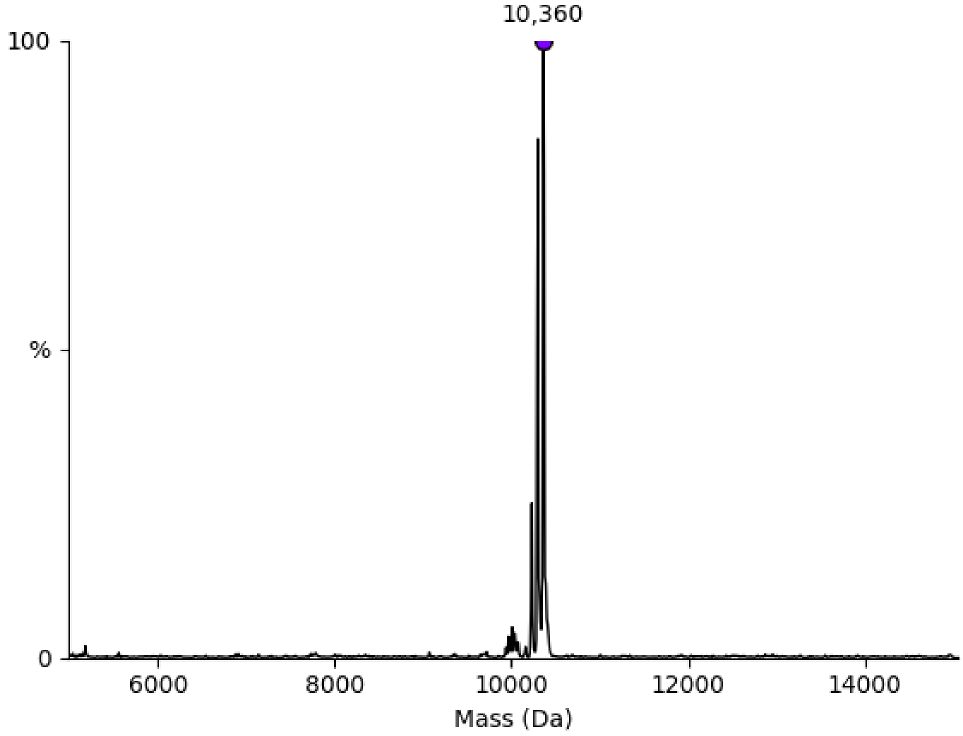

Figure S32. Deconvoluted MS spectrum of **31ON_U^{NMe}**: calculated: 10366.3 Da; found: 10360 Da, Δ = 6.3 Da

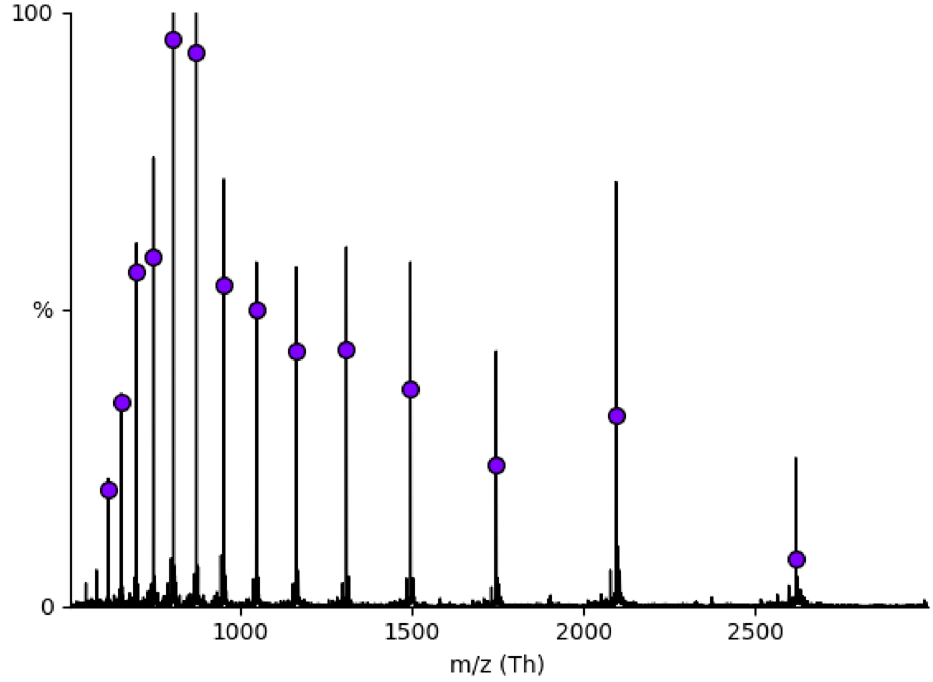

Figure S33. Raw MS spectrum of **31ON_G^{NMe2}**

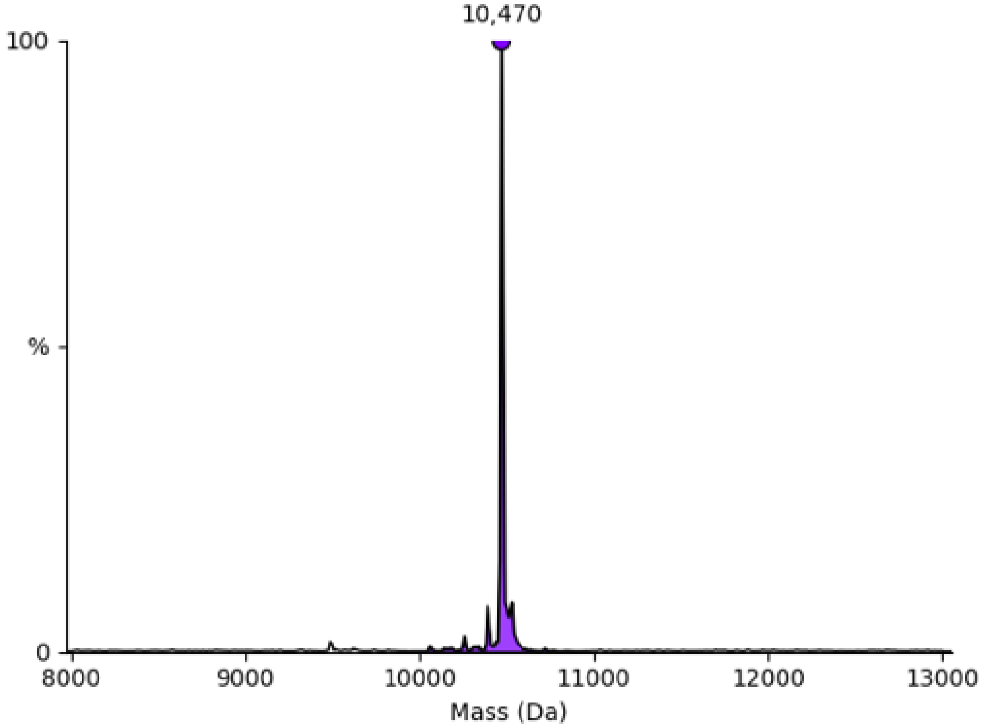

Figure S34. Deconvoluted MS spectrum of **31ON_G^{NMe2}**: calculated: 10474.3 Da; found: 10470 Da, Δ = 4.3 Da

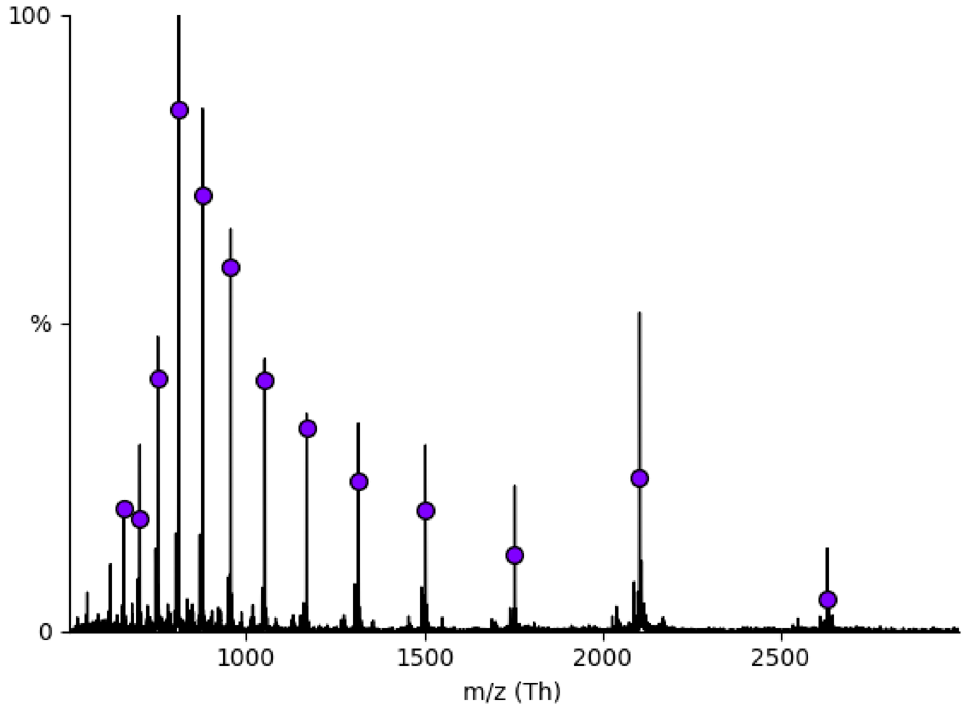

Figure S35. Raw MS spectrum of **31ON_C^{NMe3}**

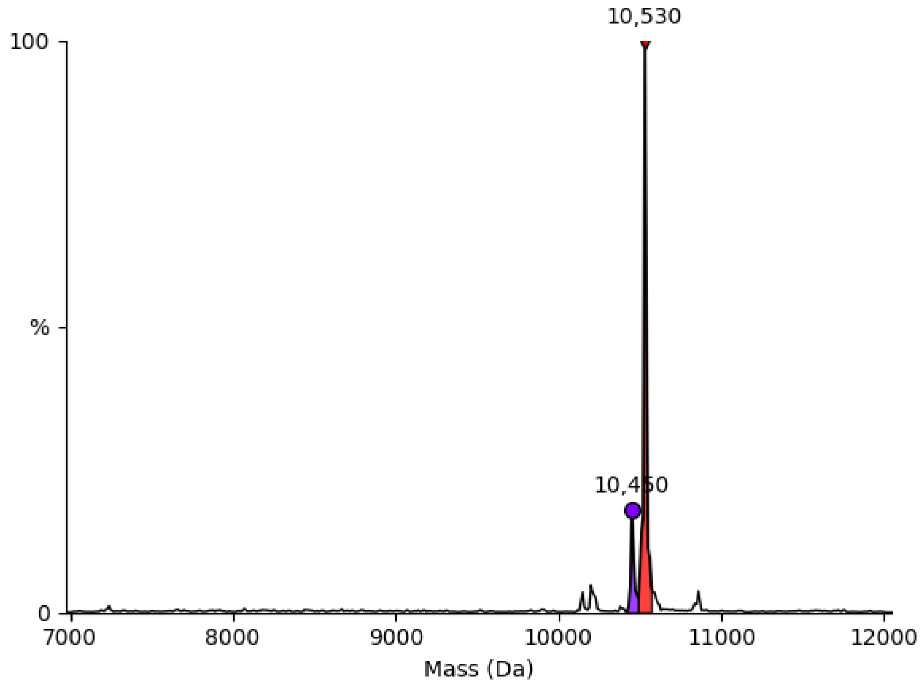

Figure S36. Deconvoluted MS spectrum of **31ON_C^{NMe3}**: calculated: 10538.3 Da; found: 10530 Da, Δ = 8.3 Da; 10450 Da corresponds to a product of dTMP misincorporation instead of **dC^{NMe3}MP** (calculated: 10457 Da).

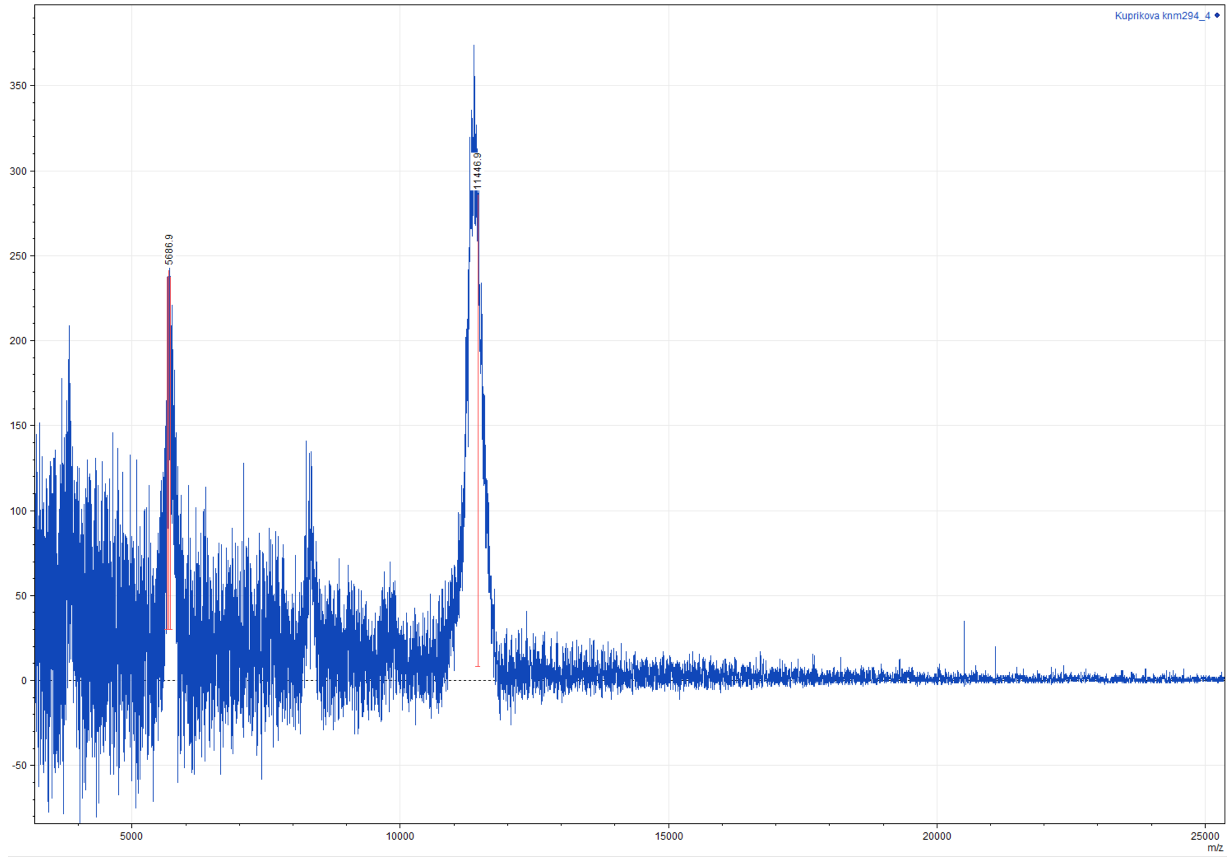

Figure S37. MALDI-TOF spectrum of **31ON_A^{NH2}U^{NMe}G^{NMe2}C^{NMe3}**

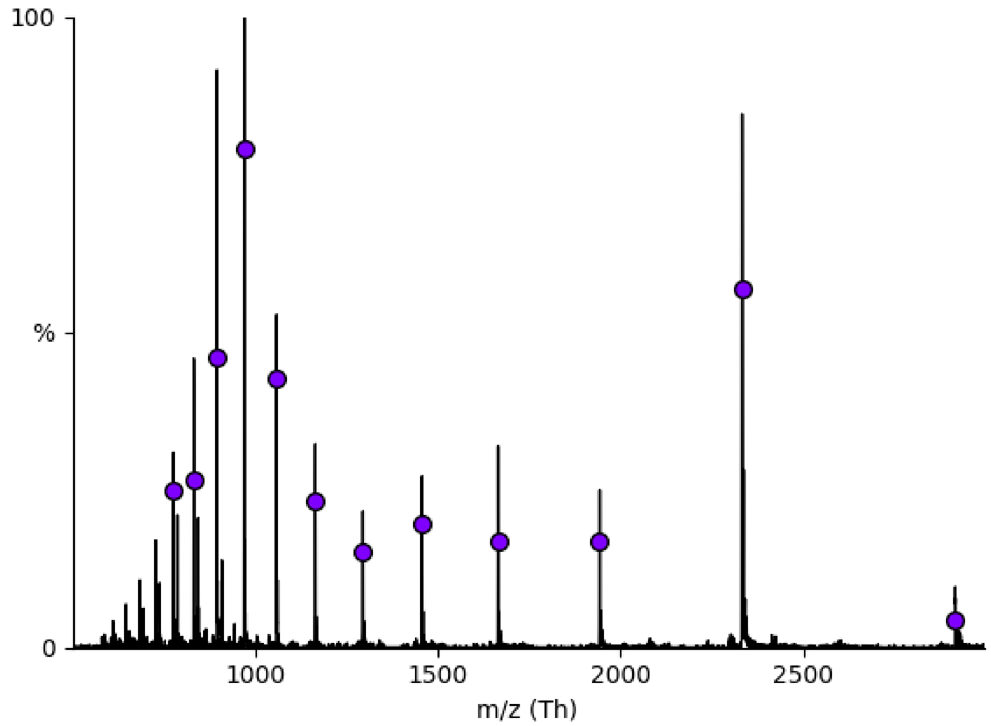

Figure S38. Raw MS spectrum of **31ON_A^{NH2}U^{EPh}G^{PA}C^{EAlk}**

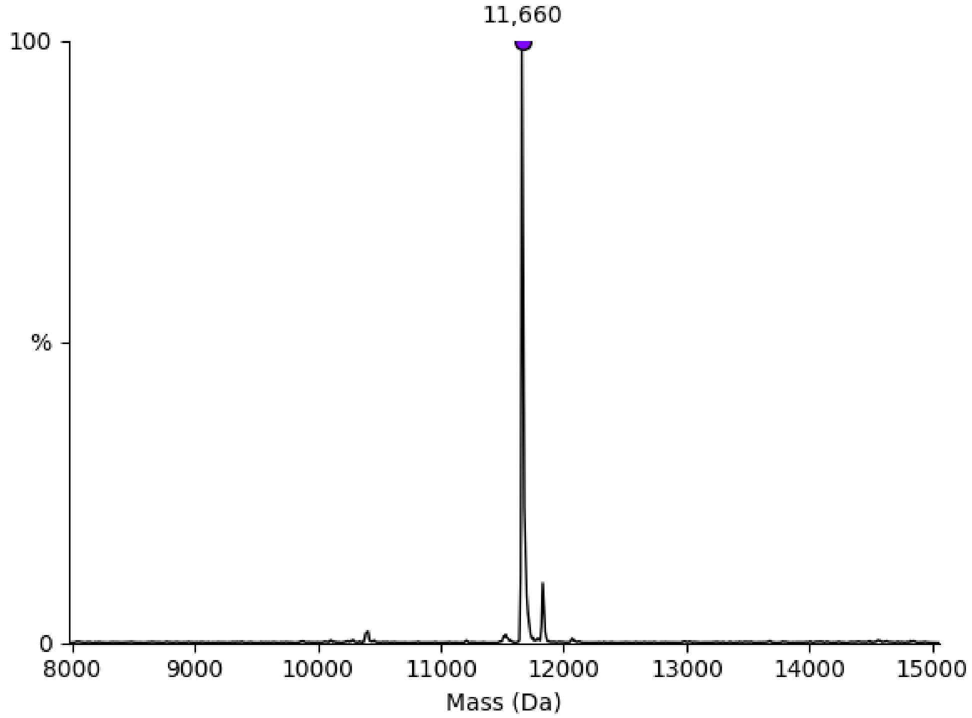

Figure S39. Deconvoluted MS spectrum of **31ON_A^{NH2}U^{EPh}G^{PA}C^{EAlk}**: calculated: 11662.3 Da; found: 11660 Da, Δ = 2.3 Da

6. Copies of NMR spectra

6.1. ^{1}H, ^{13}C and $^{31}P\{^{1}H\}$NMR spectra of dA^{NH2}TP

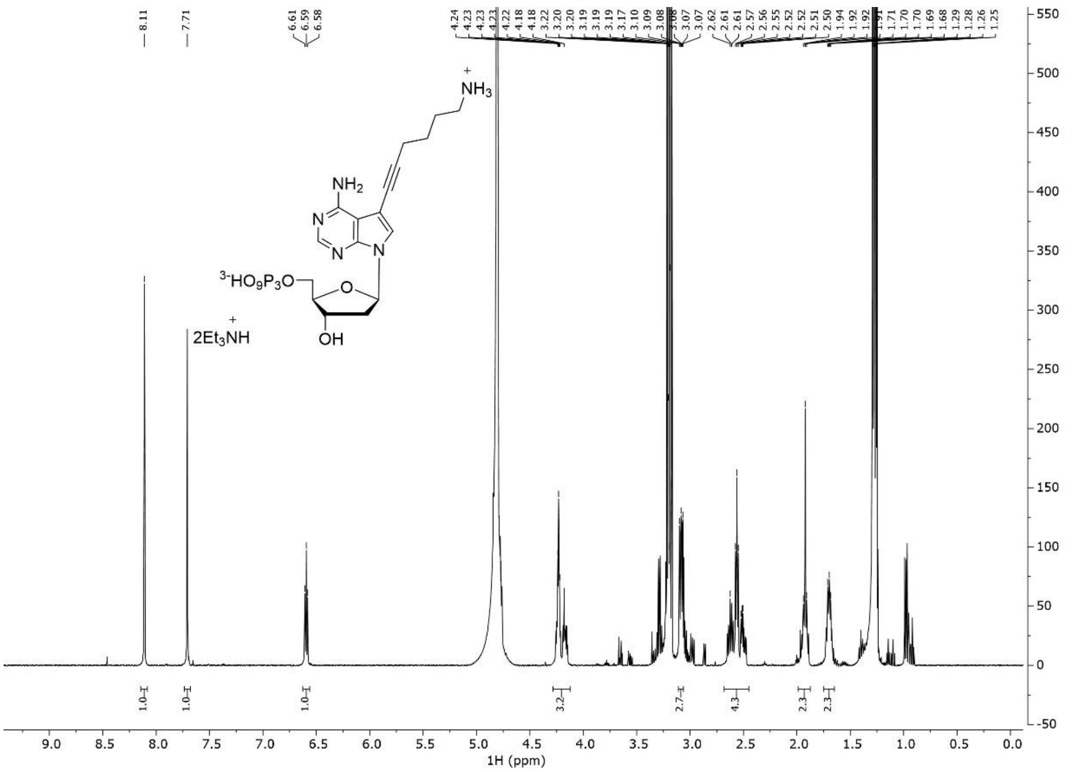

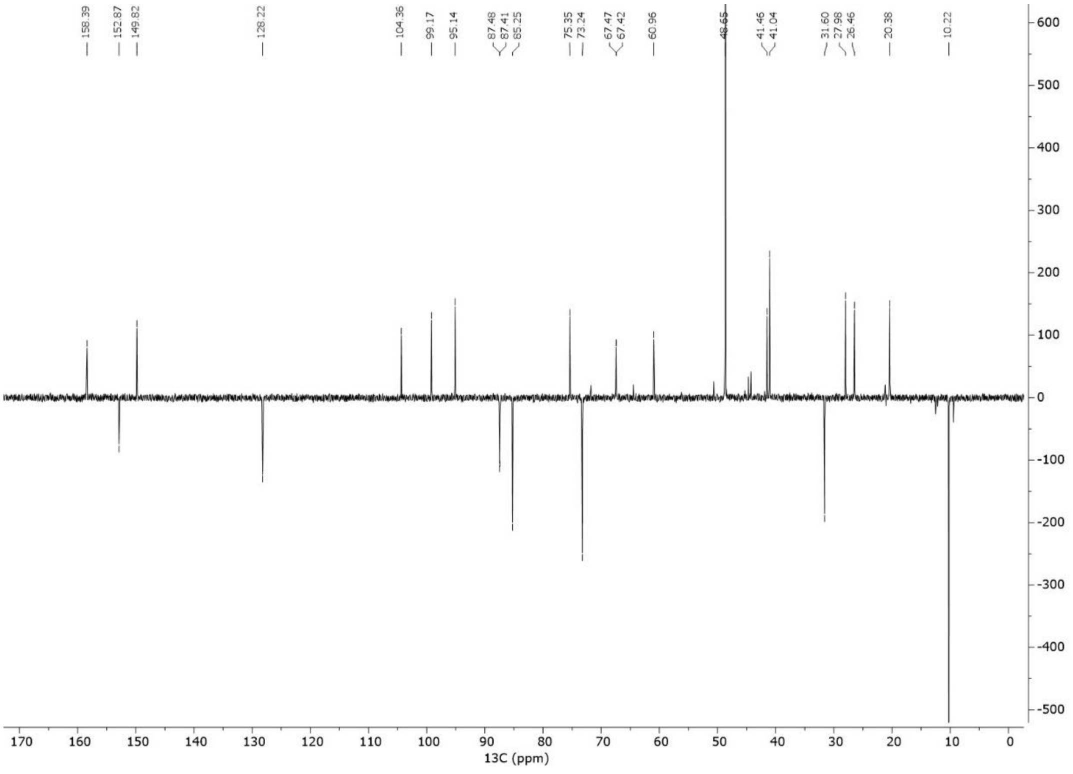

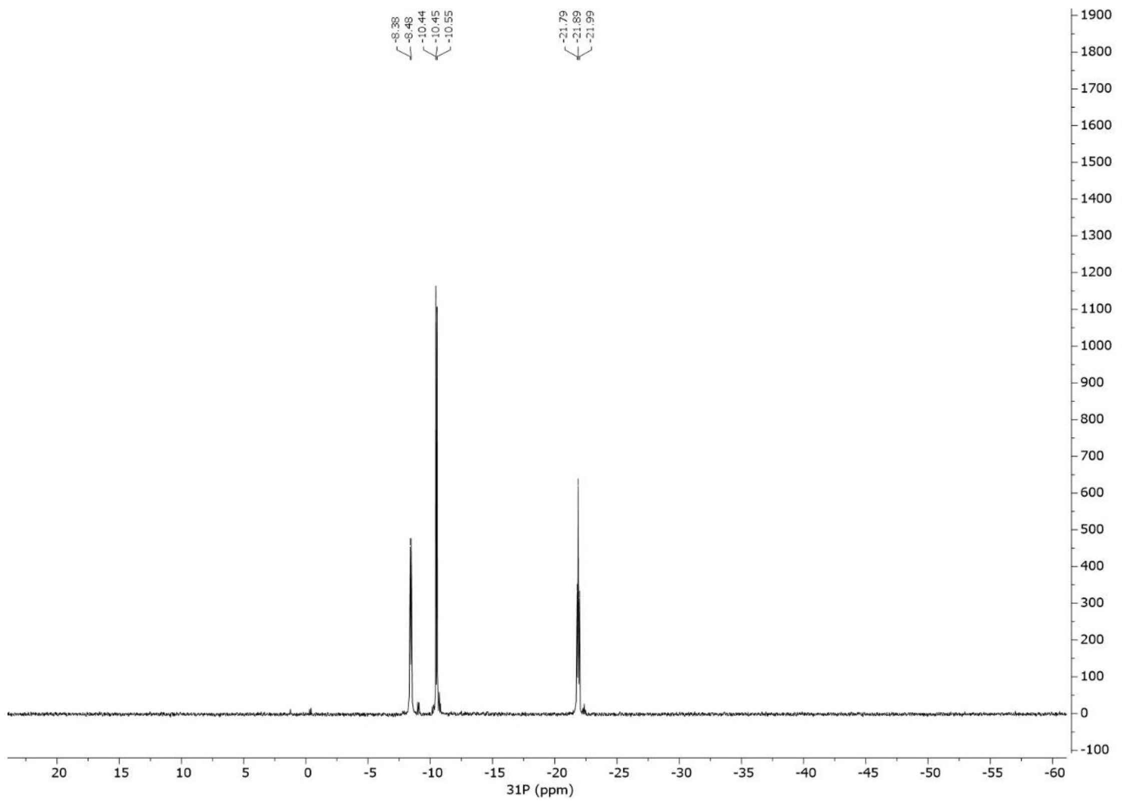

6.2. ^{1}H, ^{13}C and $^{31}P\{^{1}H\}$NMR spectra of dU^{NMe}TP

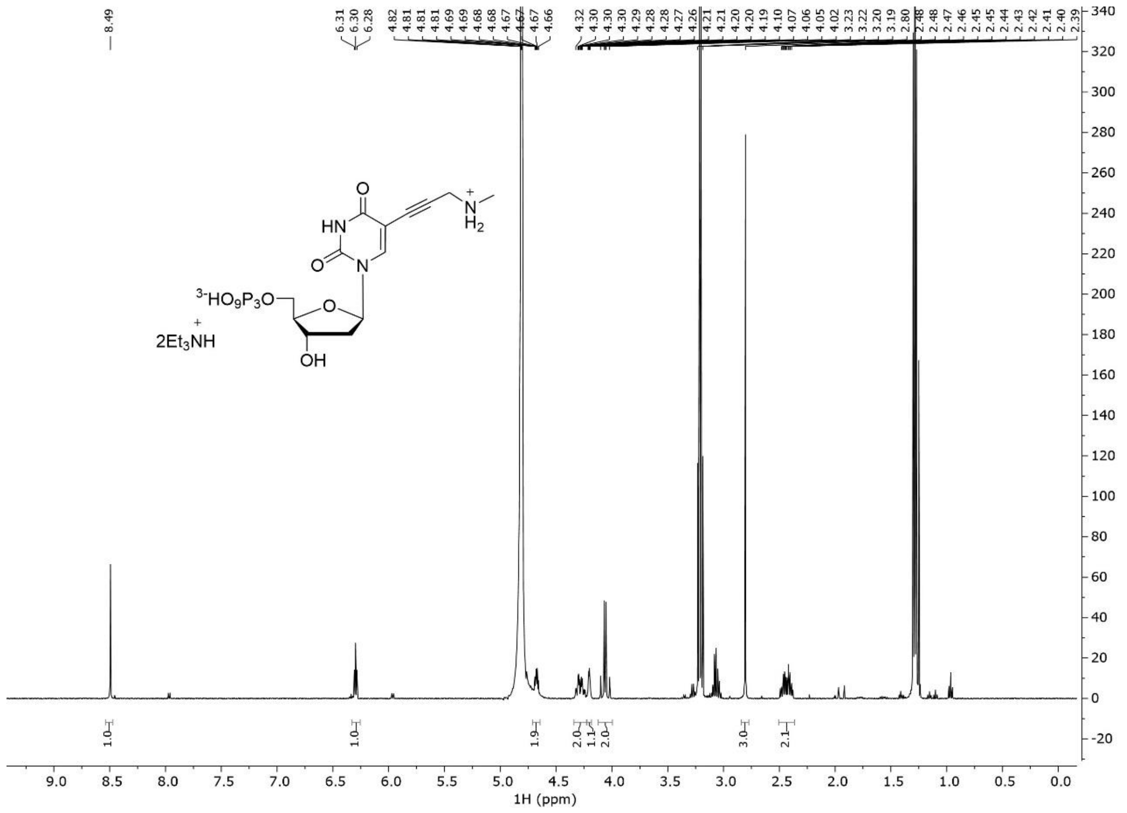

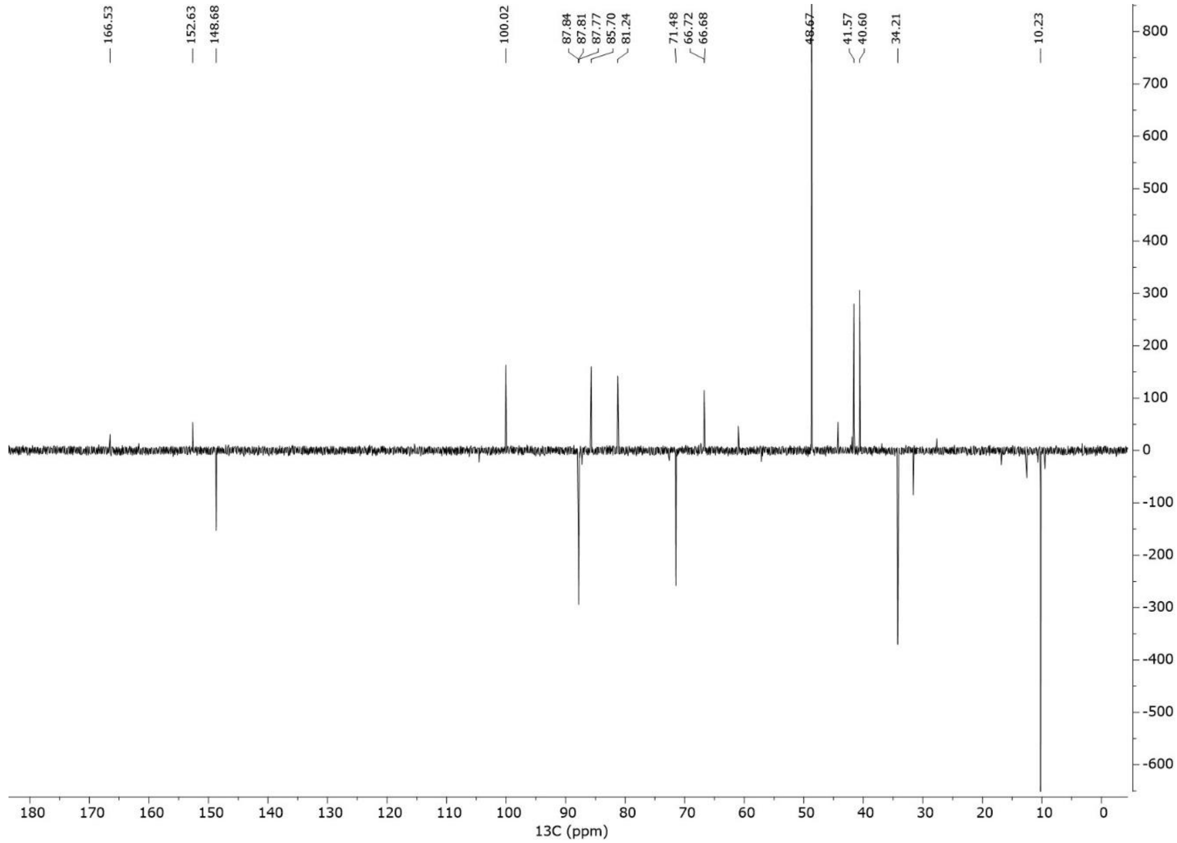
166.53
152.63
148.68
100.02
87.84
87.81
87.77
85.70
81.24
71.48
66.72
66.68
48.67
41.57
40.60
34.21
10.23
13C (ppm)

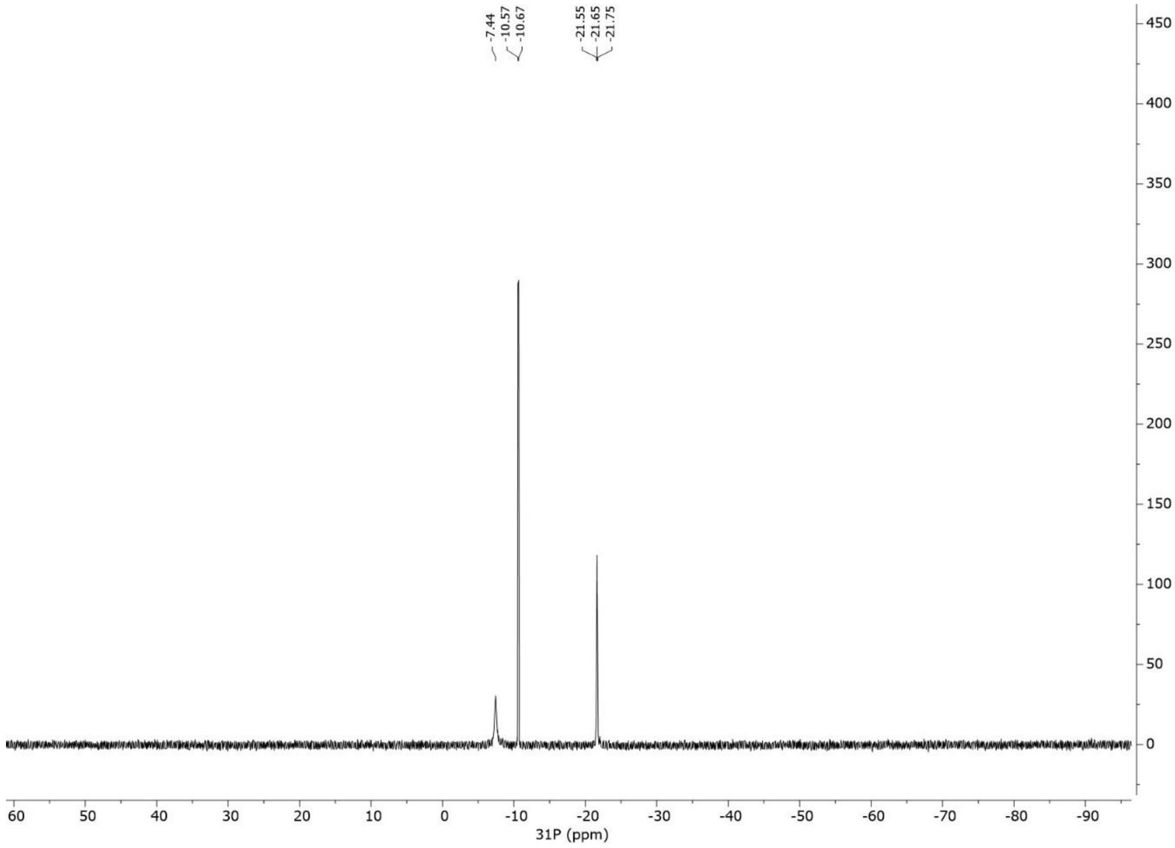
-7.44
-10.57
-10.67
-21.55
-21.65
-21.75
31P (ppm)

6.3. ^{1}H, ^{13}C and $^{31}P\{^{1}H\}$NMR spectra of dG^{NMe2}TP

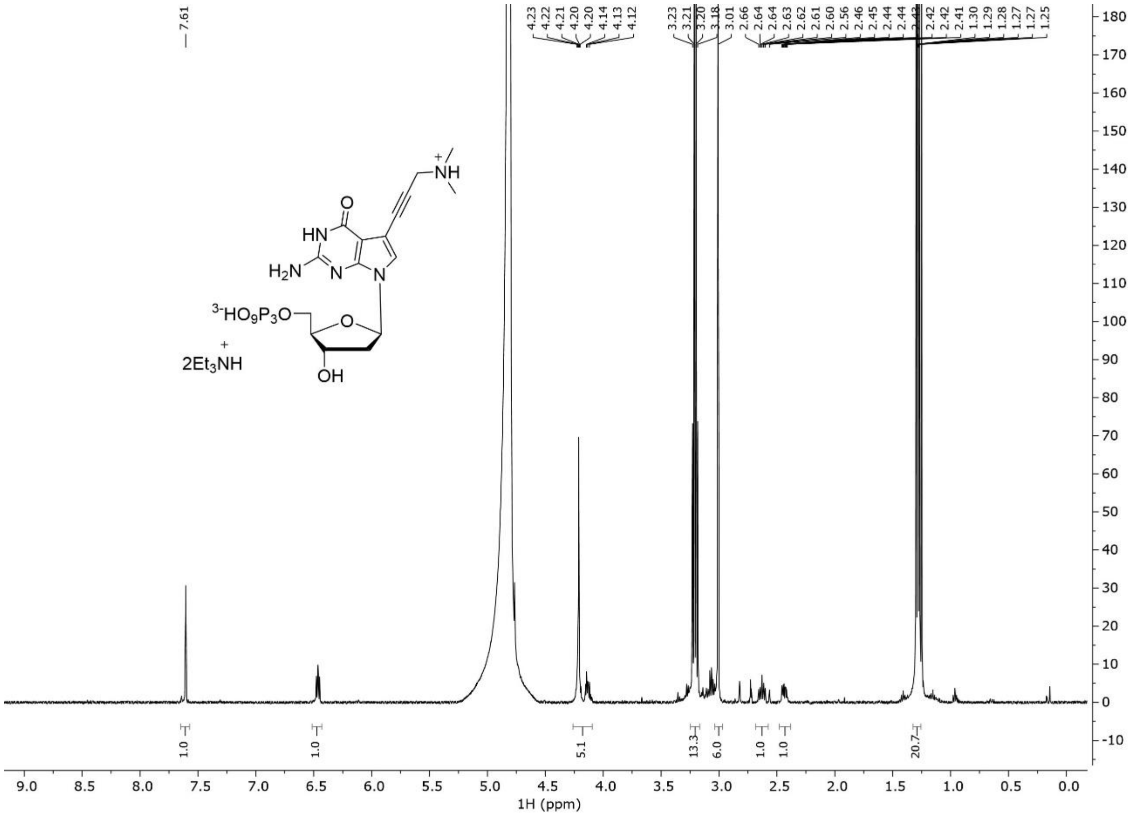

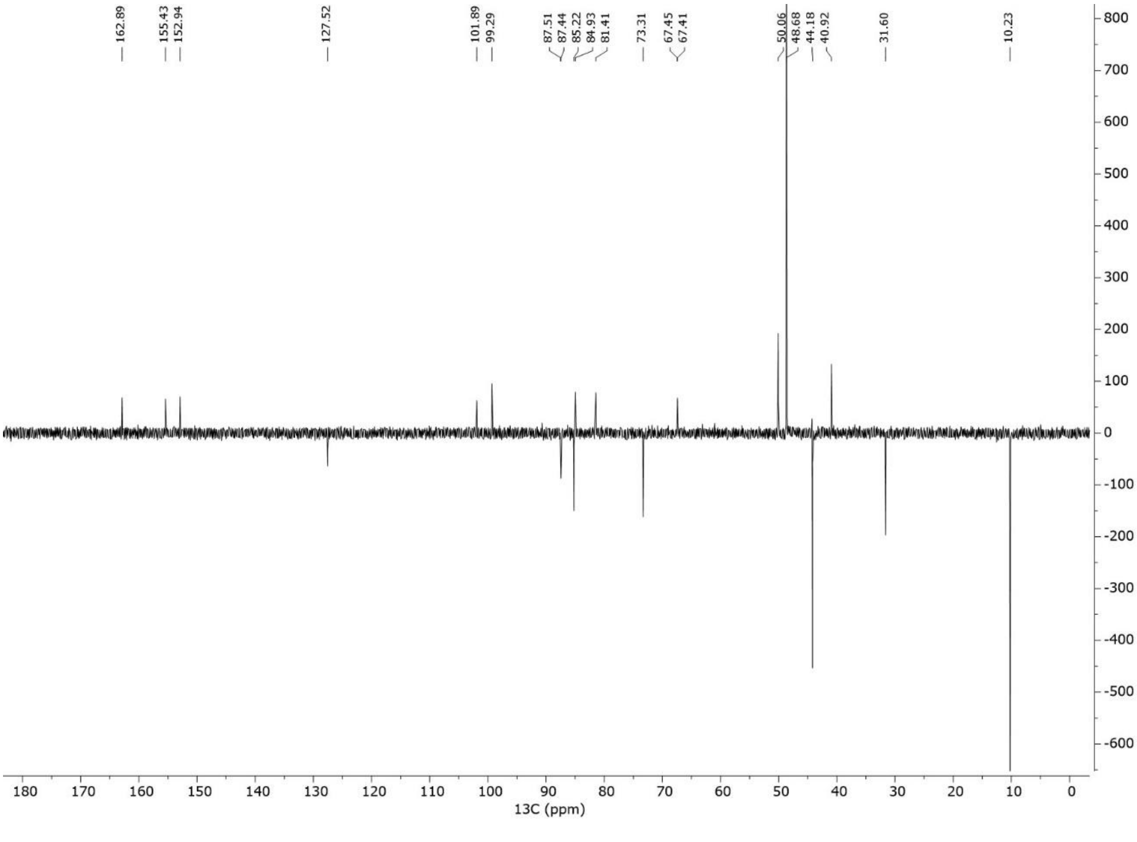

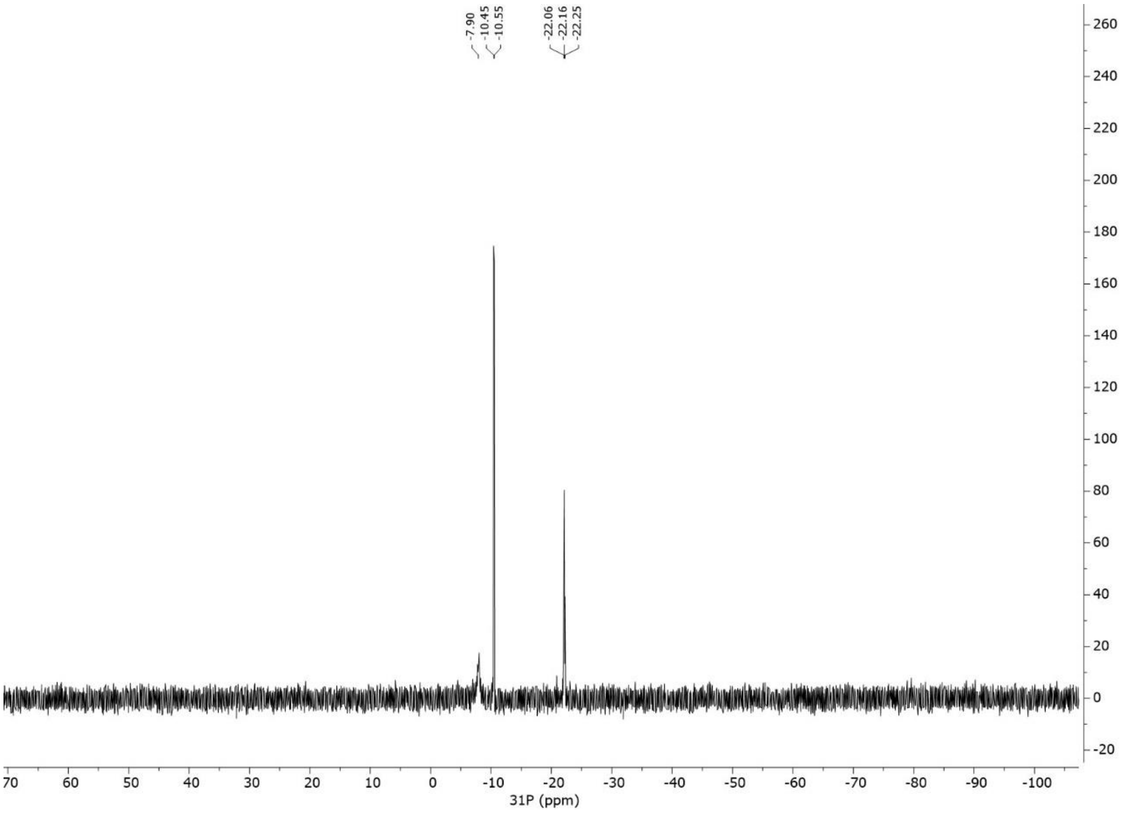

6.4. ^{1}H, ^{13}C and $^{31}P\{^{1}H\}$NMR spectra of dC^{NMe3}

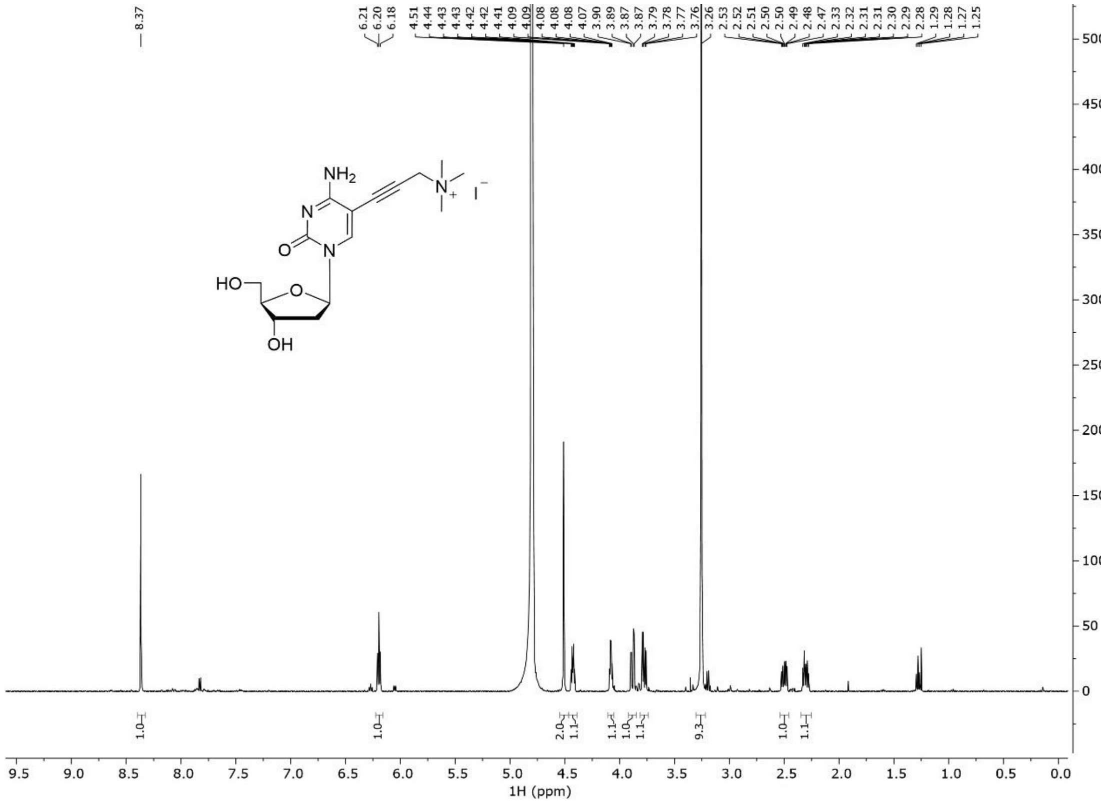

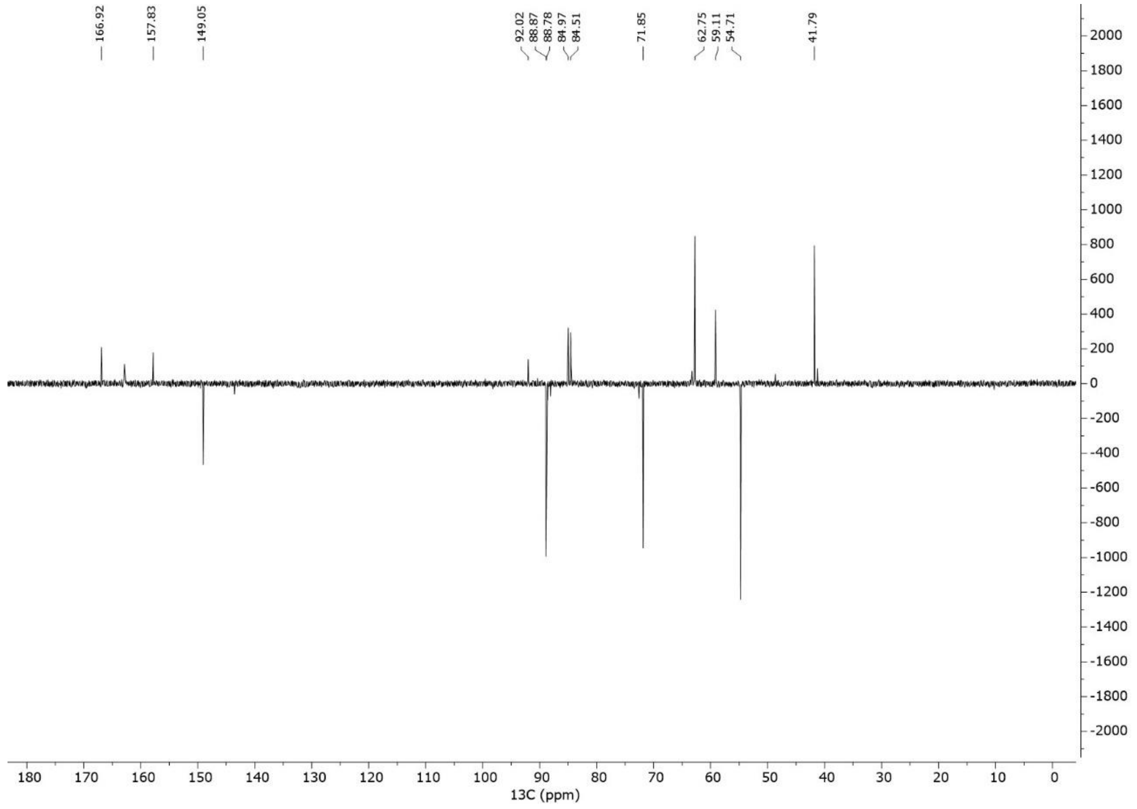

6.5. ^{1}H, ^{13}C and $^{31}P\{^{1}H\}$NMR spectra of dC^{NMe3}TP

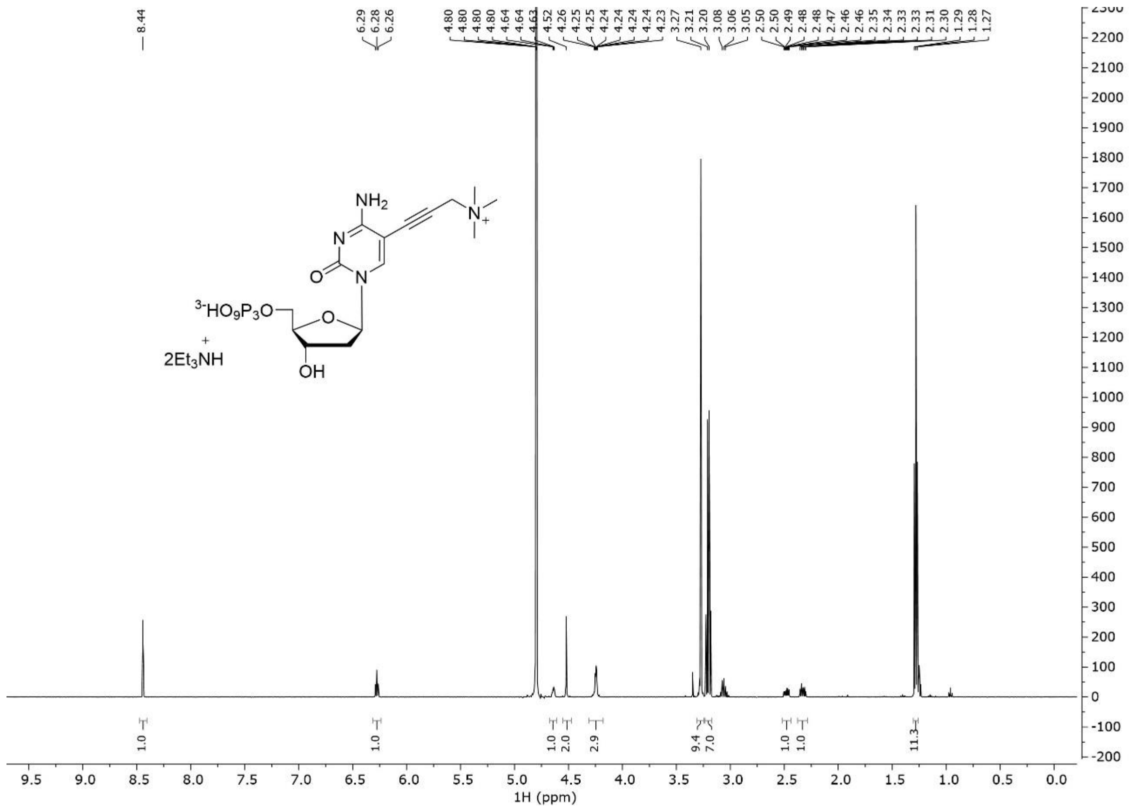

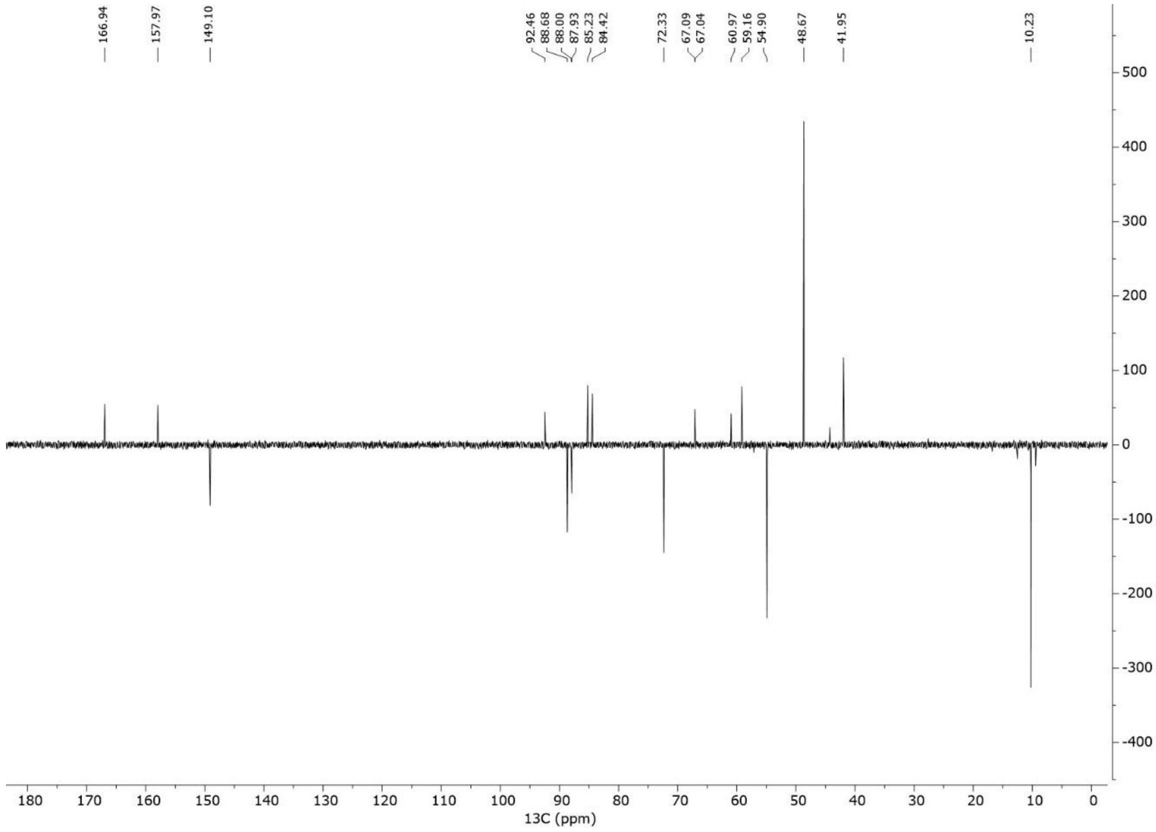
166.94
157.97
149.10
92.46
88.68
88.00
87.93
85.23
84.42
72.33
67.09
67.04
60.97
59.16
54.90
48.67
41.95
10.23
13C (ppm)

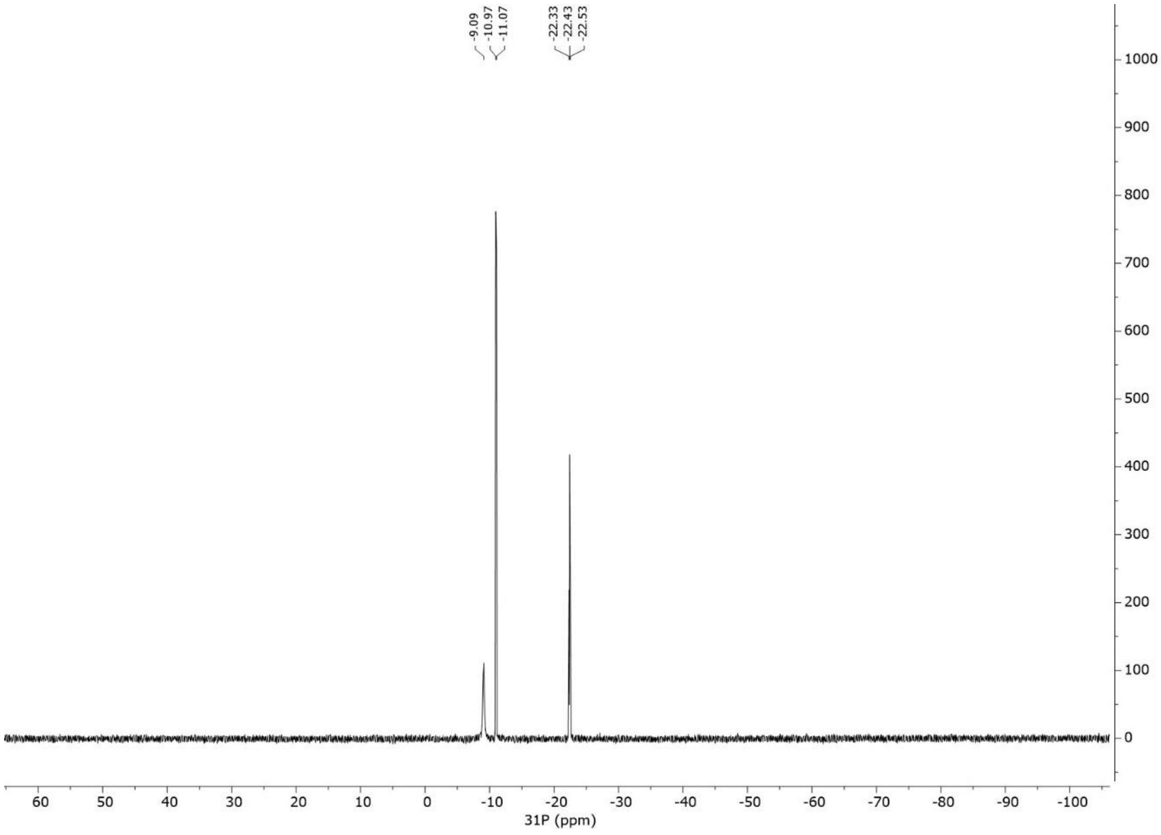
-9.09
-10.97
-11.07
-22.33
-22.43
-22.53
31P (ppm)

7. HPLC traces of modified dNTPs

7.1. HPLC traces of dA^{NH2}TP

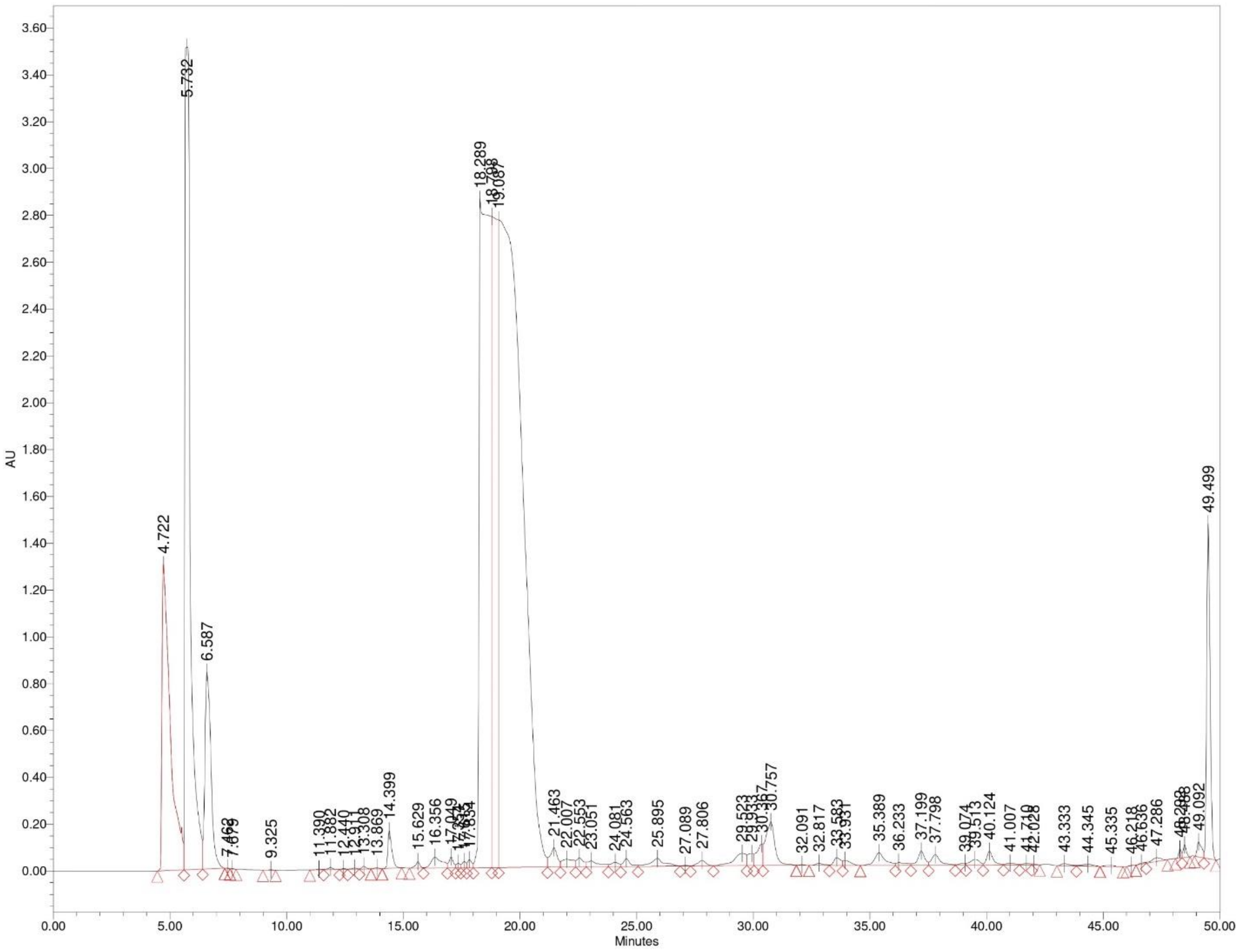

Flow rate 10 ml/min; column – Waters X-Bridge Shield RP18; eluent – 10 to 100% of buffer B in buffer A in 40 min (last 10 min of the run include column wash with 100% MeOH); the product elutes at 18.289 min.

7.2. HPLC traces of dU^{NMe}TP

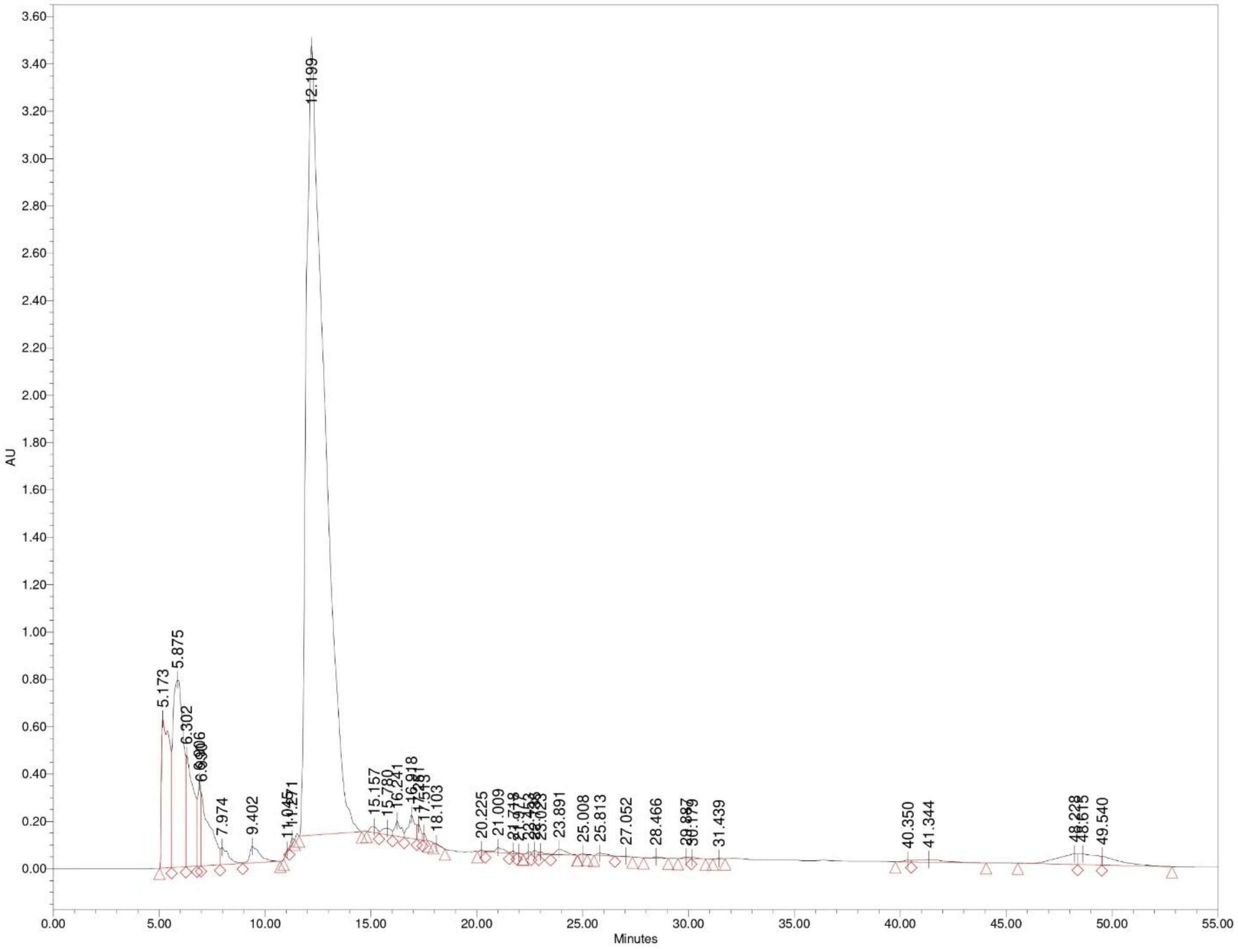

Flow rate 10 ml/min; column – Phenomenex Kinetex EVO C18; eluent – 0 to 100% of buffer B in buffer A in 45 min (last 10 min of the run include column wash with 100% MeOH); the fraction eluted at 12.199 min was further purified on Sepharose DEAE Fast Flow.

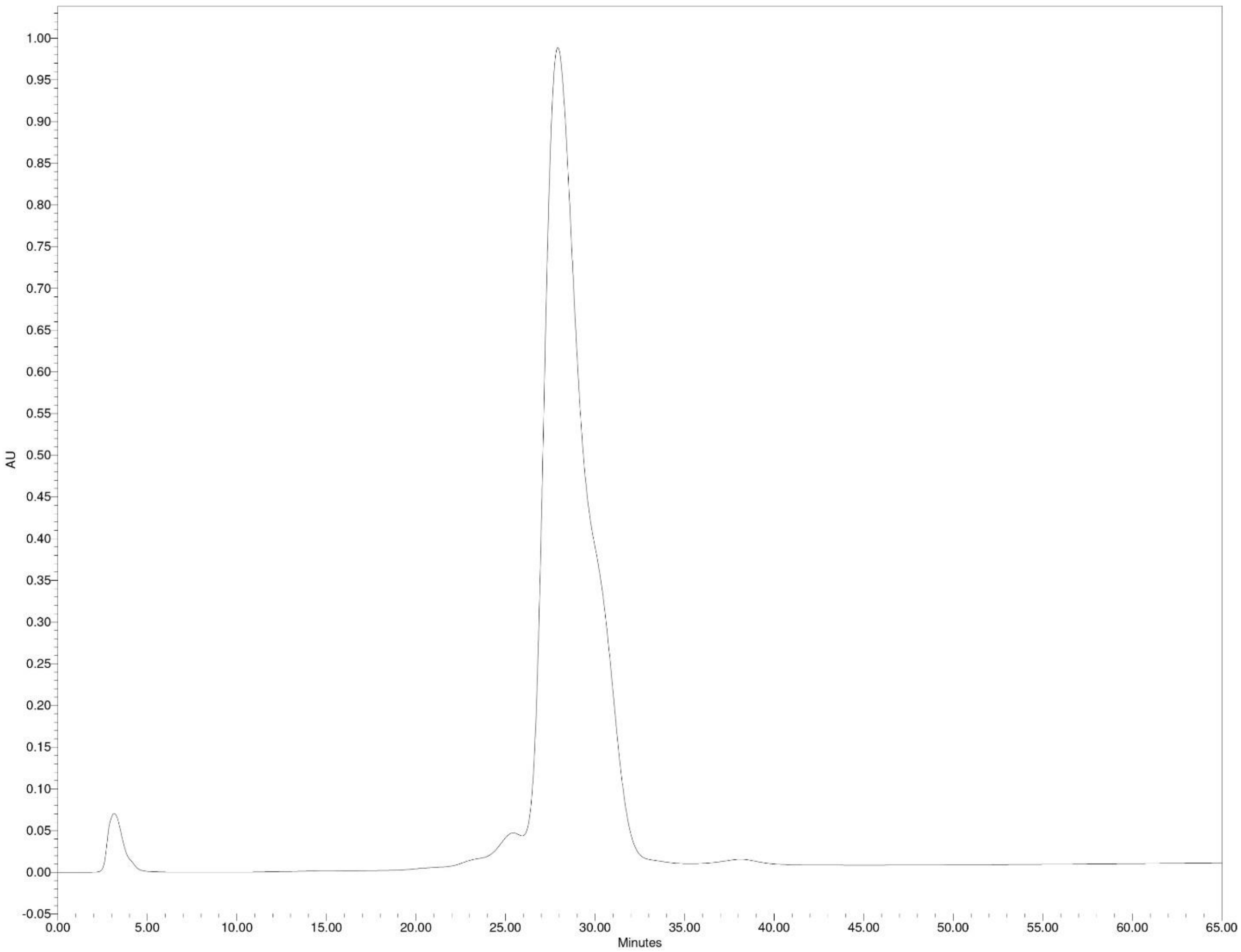

Flow rate 12 ml/min; column – Sepharose DEAE Fast Flow; eluent – 0 to 100% of 0.8 M TEAB buffer in water in 100 min (the run was terminated earlier); the product elutes at ~28 min.

7.3. HPLC traces of dG^{NMe2}TP

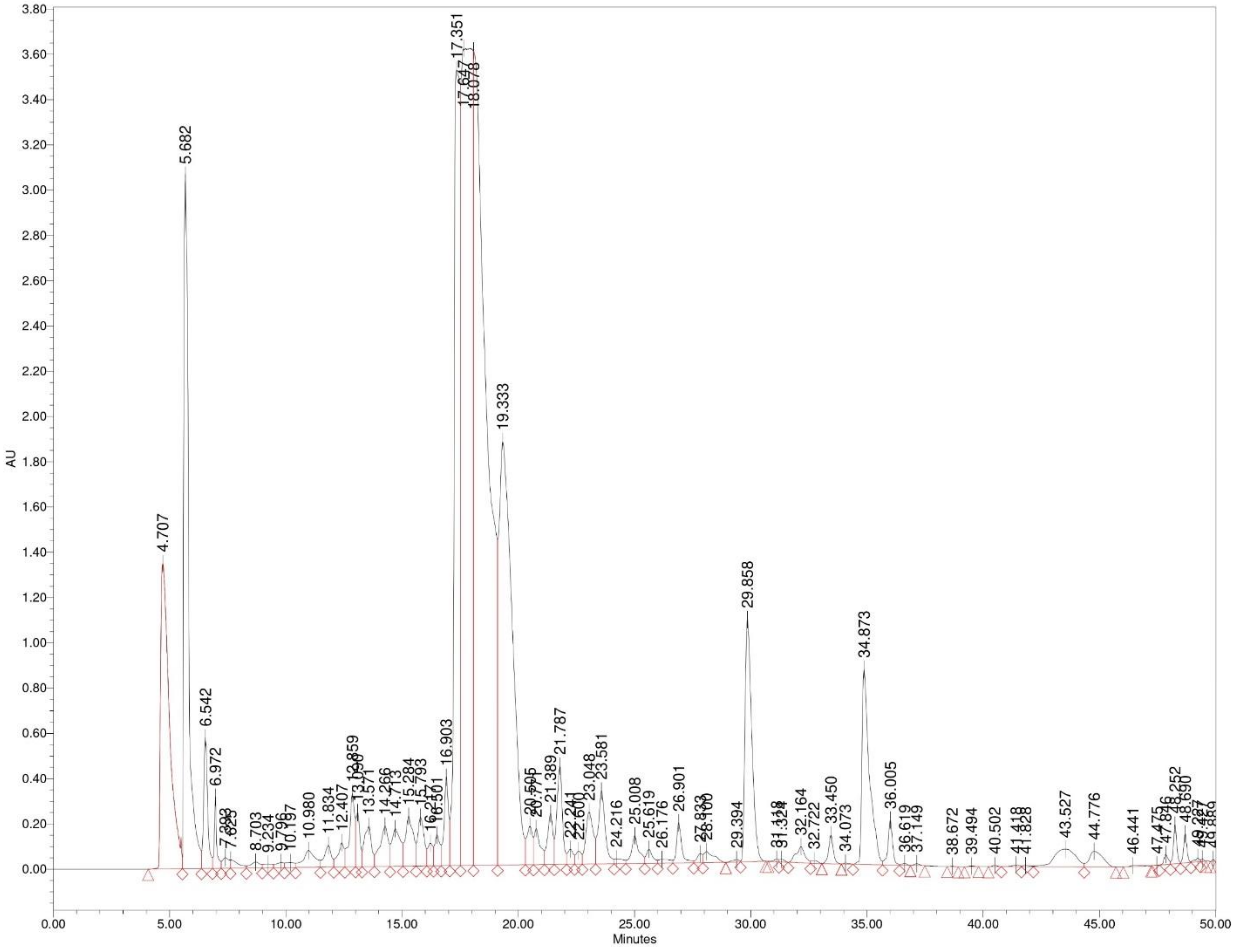

Flow rate 10 ml/min; column – Waters X-Bridge Shield RP18; eluent –10 to 100% of buffer B in buffer A in 40 min (last 10 min of the run include column wash with 100% MeOH); the product elutes at 17.351 min.

7.4. HPLC traces of dC^{NMe3}

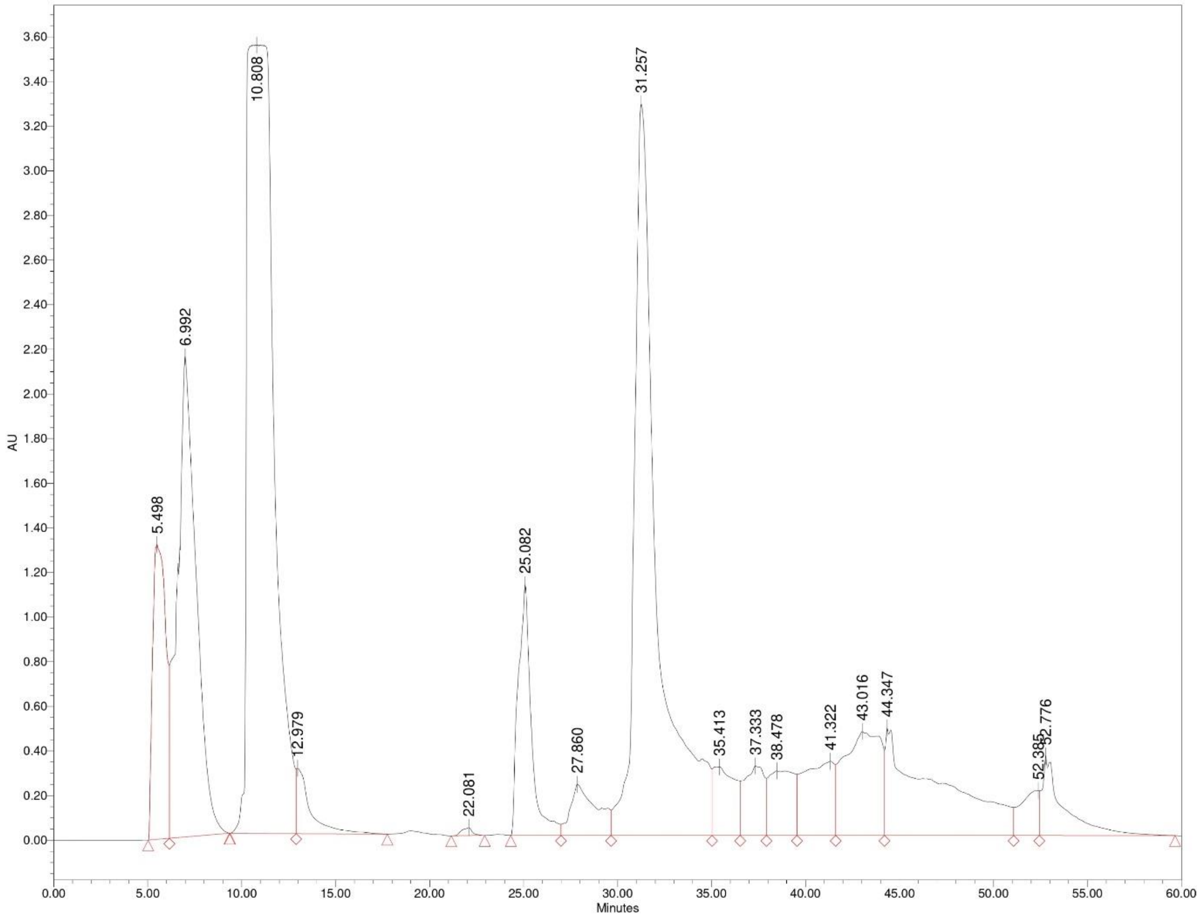

Flow rate 10 ml/min; column – Phenomenex Kinetex EVO C18; eluent – 0 to 50% of MeOH in water in 35 min followed by 50 to 100% of MeOH in water in 15 min (last 10 min of the run include column wash with 100% MeOH); the product elutes at 10.808 min.

7.5. HPLC traces of dC^{NMe3}TP

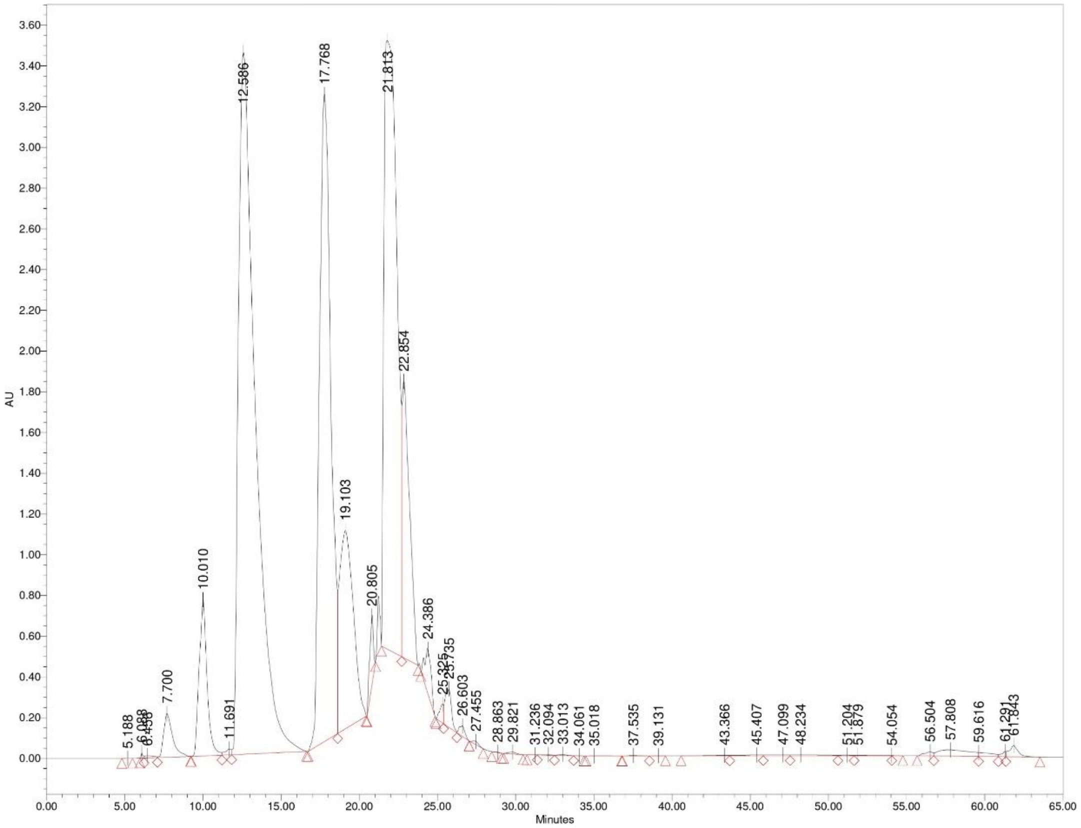

Flow rate 10 ml/min; column – Phenomenex Kinetex EVO C18; eluent – 0 to 100% of buffer B in buffer A in 50 min (last 15 min of the run include column wash with 100% MeOH); the fraction eluted at 17.800 min was further purified on Sepharose DEAE Fast Flow.

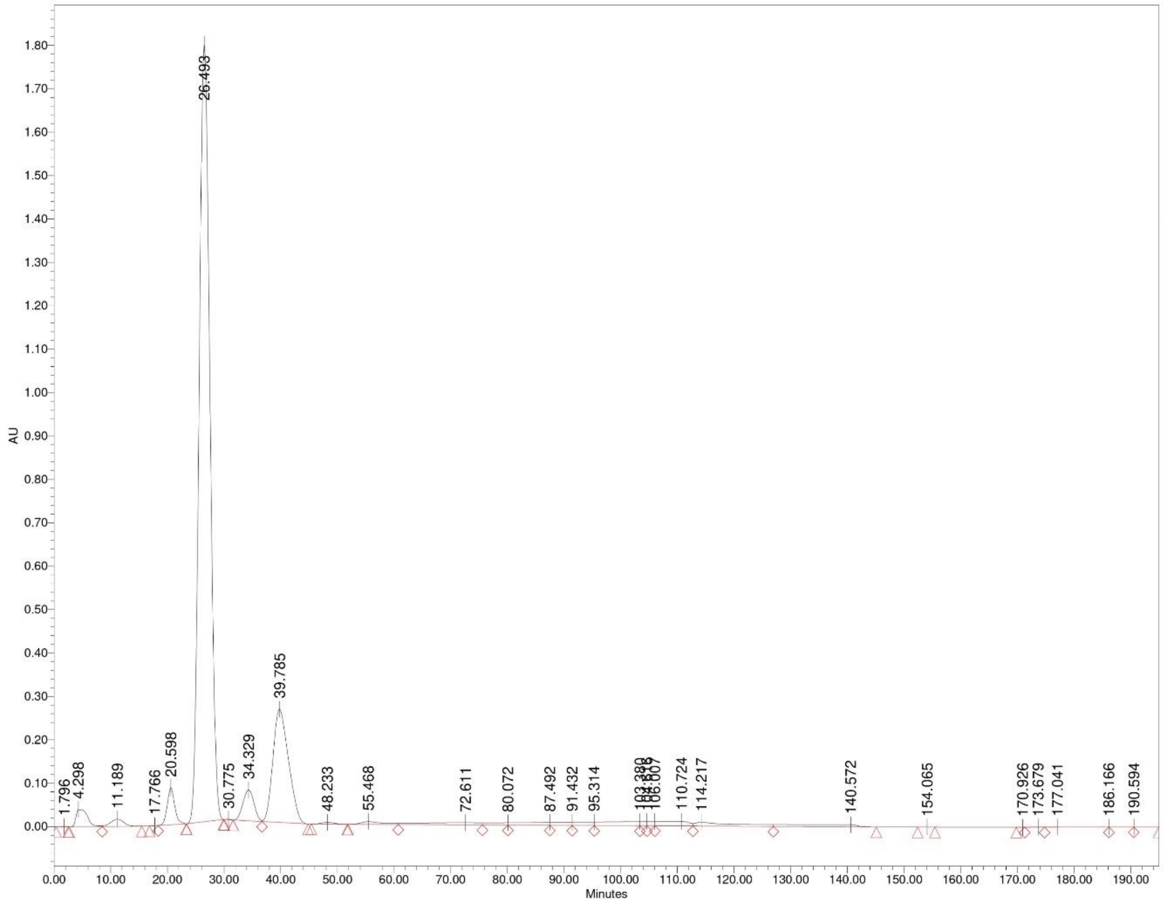

Flow rate 8 ml/min; column – Sepharose DEAE Fast Flow; eluent – 0 to 100% of 0.8 M TEAB buffer in water in 100 min (last 90 min of the run include 30 min column wash with 1 M $NaHCO_3$, 30 min with water, and 30 min with 20% MeOH in water); the product elutes at 26.493 min.

8. References

1. Čapek,P., Cahová,H., Pohl,R., Hocek,M., Gloeckner,C., Marx,A. (2007) An Efficient Method for the Construction of Functionalized DNA Bearing Amino Acid Groups through Cross-Coupling Reactions of Nucleoside Triphosphates Followed by Primer Extension or PCR. *Chem. Eur. J.*, **13**, 6196-6203.
2. Le,B.H., Koo,J.Ch., Joo,H.N., Seo,Y.J. (2017) Diverse size approach to incorporate and extend highly fluorescent unnatural nucleotides into DNA. *Bioorg. Med. Chem.*, **25**, 3591-3596.
3. Sýkorová,V., Tichý,M., Hocek,M. (2022) Polymerase Synthesis of DNA Containing Iodinated Pyrimidine or 7-Deazapurine Nucleobases and Their Post-synthetic Modifications through the Suzuki-Miyaura Cross-Coupling Reactions. *ChemBioChem*, **23**, e202100608.
4. Kim,Y.J., Lek,M.T., Schramm, M.P. (2011) pH Influenced molecular switching with micelle bound cavitands. *Chem. Commun.*, **47**, 9636-9638.
5. Ondruš,M., Sýkorová,V., Bednárová,L., Pohl,R., Hocek,M. (2020) Enzymatic synthesis of hypermodified DNA polymers for sequence-specific display of four different hydrophobic groups. *Nucleic Acids Res.*, **48**, 11982-11993.
6. Kuprikova,N., Ondruš,M., Bednárová,L., Riopedre-Fernandez,M., Poštová Slavětínská,L., Sýkorová,V., Hocek,M. (2023) Superanionic DNA. Enzymatic Synthesis of Hypermodified DNA Bearing Four Different Anionic Substituents at all Four Nucleobases. *Nucleic Acids Res.*, **51**, 11428–11438.
7. Marty,M.T., Baldwin,A.J., Marklund,E.J., Hochberg,G.K.A., Benesch,J.L.P., Robinson,C.V. (2015) Bayesian Deconvolution of Mass and Ion Mobility Spectra: From Binary Interactions to Polydisperse Ensembles. *Anal. Chem.*, **87**, 4370−4376.
